# Supplementary material for: AAV-based gene therapy with modified HEXB confers lasting therapeutic benefits in GM2 gangliosidosis models
Source: Cell Rep Med. 2026 Apr 22;7(5):102762. doi: 10.1016/j.xcrm.2026.102762 (PMC13198282; doi:10.1016/j.xcrm.2026.102762)
Supplement: Document S1. Figures S1–S6 and Tables S1, S3, S5, S6–S12, and S14 [file mmc1.pdf]

Cell Reports Medicine, Volume *7*

## Supplemental information

# AAV-based gene therapy with modified *HEXB* confers lasting therapeutic benefits in GM2 gangliosidosis models

**Keisuke Kitakaze, Yukiya Ohnishi, Daisuke Tsuji, Ryosuke Watanabe, Nijiho Kamori, Yuko Katakai, Hiroaki Shibata, Sota Yoshizawa, Mika Ito, Naomi Takino, Shin-ichi Muramatsu, and Kohji Itoh**

## Supplementary information

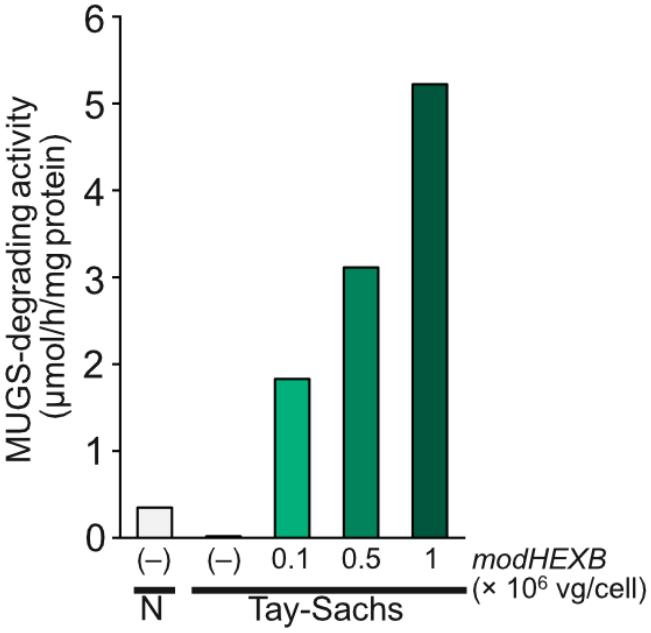


Fig. S1. Restoration of β-Hex activity in cultured human neuronal cells differentiated from iPS cells derived from patients with Tay-Sachs disease. Intracellular MUGS-degrading β-Hex activity after transduction with AAV9/3-*modHEXB* (n =1). N: normal.

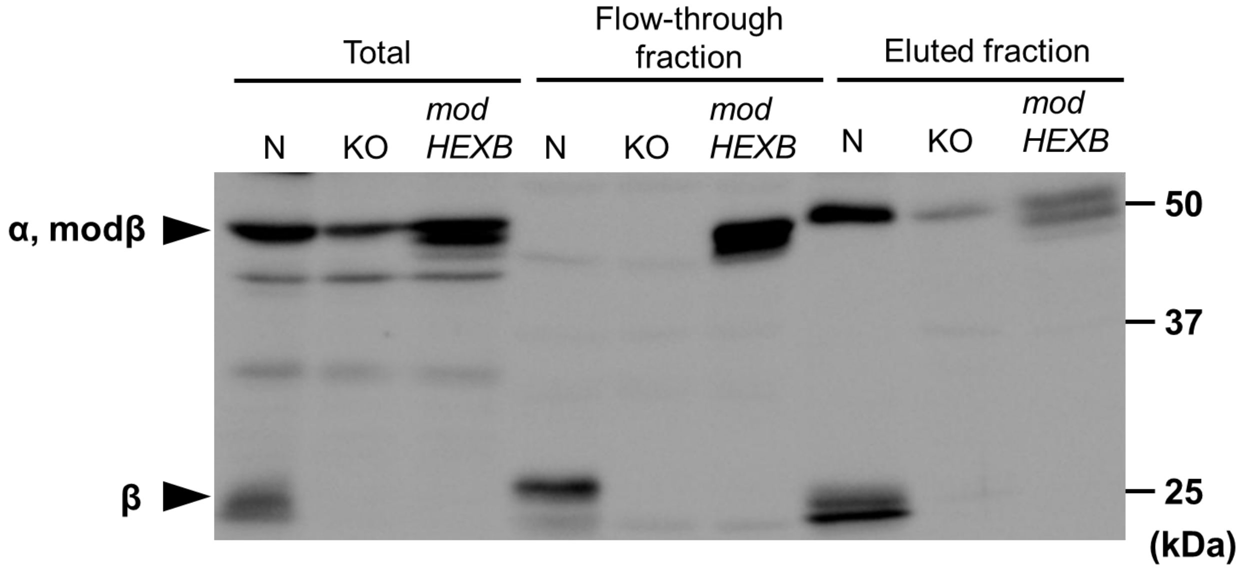


Fig. S2. Isoenzyme composition of β-hexosaminidase analyzed by Q-column chromatography in SH-SY5Y cells. Cell extracts from normal SH-SY5Y cells (N), *HEXB* knockout (KO) SH-SY5Y cells, and *HEXB* KO SH-SY5Y cells transduced with AAV9/3-*modHEXB* (*modHEXB*) were subjected to Q-column anion-exchange chromatography. The flow-through fractions contained endogenous HexB (β/β) and/or transgene-derived modHexB (modβ/modβ), whereas the eluted fractions contained HexA (α/β) and HexS (α/α). Fractions were analyzed by SDS–PAGE followed by immunoblotting using an anti-HexA antibody.

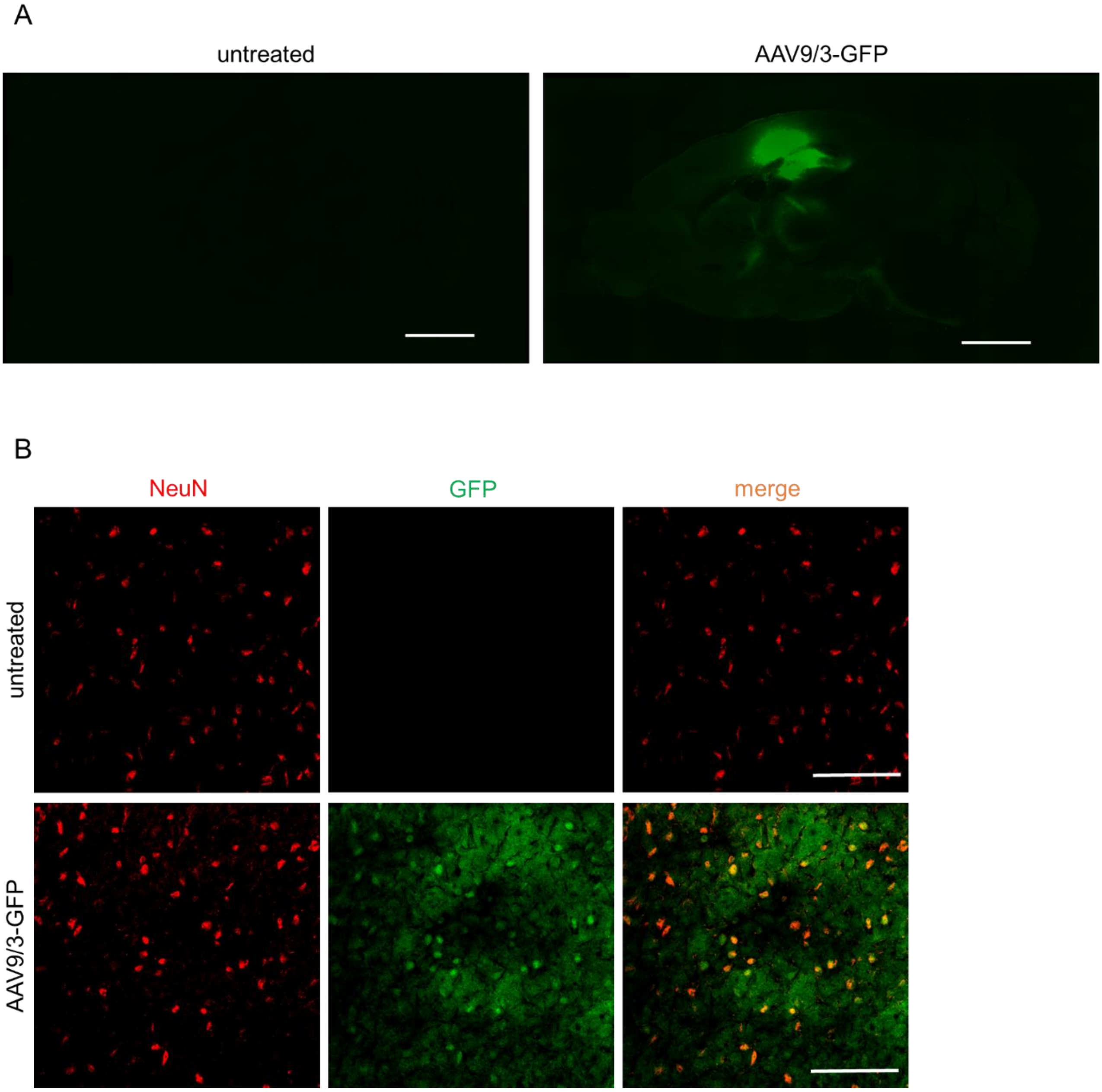


Fig. S3. Immunohistochemical analyses of the brain from wild-type mice intracerebroventricularly administered AAV9/3-GFP. (**A**) GFP in sagittal sections of the whole brain. Green: GFP. Scale bars: 2 mm. (**B**) Co-localization of NeuN and GFP in cerebral. Red: NeuN, green: GFP. Scale bars: 2 mm.

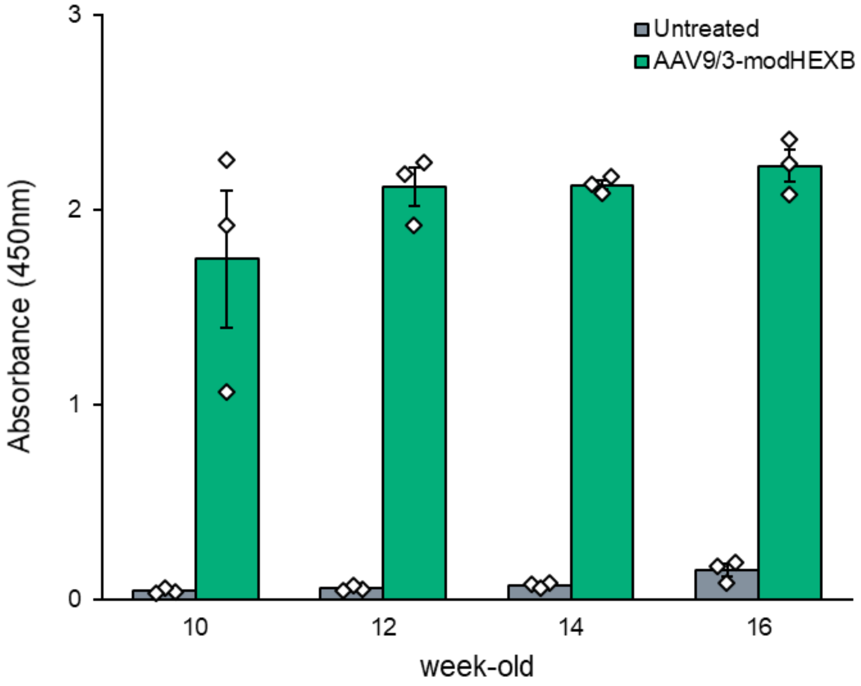


Fig. S4. Detection of anti-modHexB antibodies in SD mice by ELISA. Serum anti-modHexB antibody levels were measured by ELISA using purified recombinant modHexB as the coating antigen. Sera were collected from untreated SD mice and from SD mice treated with AAV9/3-*modHEXB* at 6 weeks of age ($5.8 \times 10^{12}$ vg/kg BW), diluted 1:1,000. Bound antibodies were detected using an HRP-conjugated anti-mouse Ig secondary antibody and quantified by absorbance at 450 nm. Data are shown as mean ± SEM (n = 3).

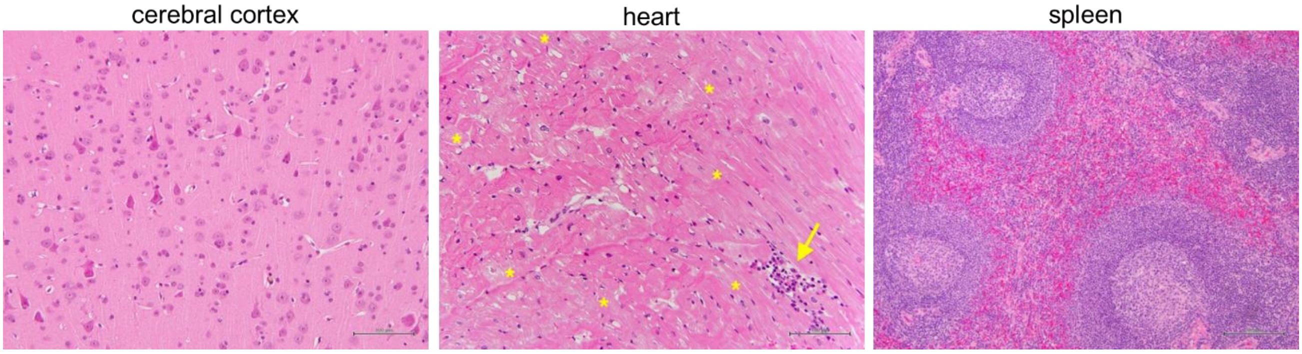


Fig. S5. Representative histopathological findings in non-human primates following *i.t.* administration of AAV9/3-*modHEXB* ($2.0 \times 10^{12}$ vg/kg). Left panel: Cerebral cortex (parietal lobe) from animal #1 showing mild gliosis with increased microglial cells, accompanied by a diffuse increase in neuronophagia and eosinophilic neurons. Middle panel: Heart from animal #3 demonstrating focal contraction band necrosis of cardiomyocytes in the left ventricular wall (outlined area), without progression to liquefactive necrosis. Multifocal, minimal mononuclear cell infiltration associated with localized cardiomyocyte degeneration is also observed (yellow arrows). Right panel: Spleen from animal #3 showing diffuse lymphoid follicular hyperplasia. Scale bars, 100 µm.

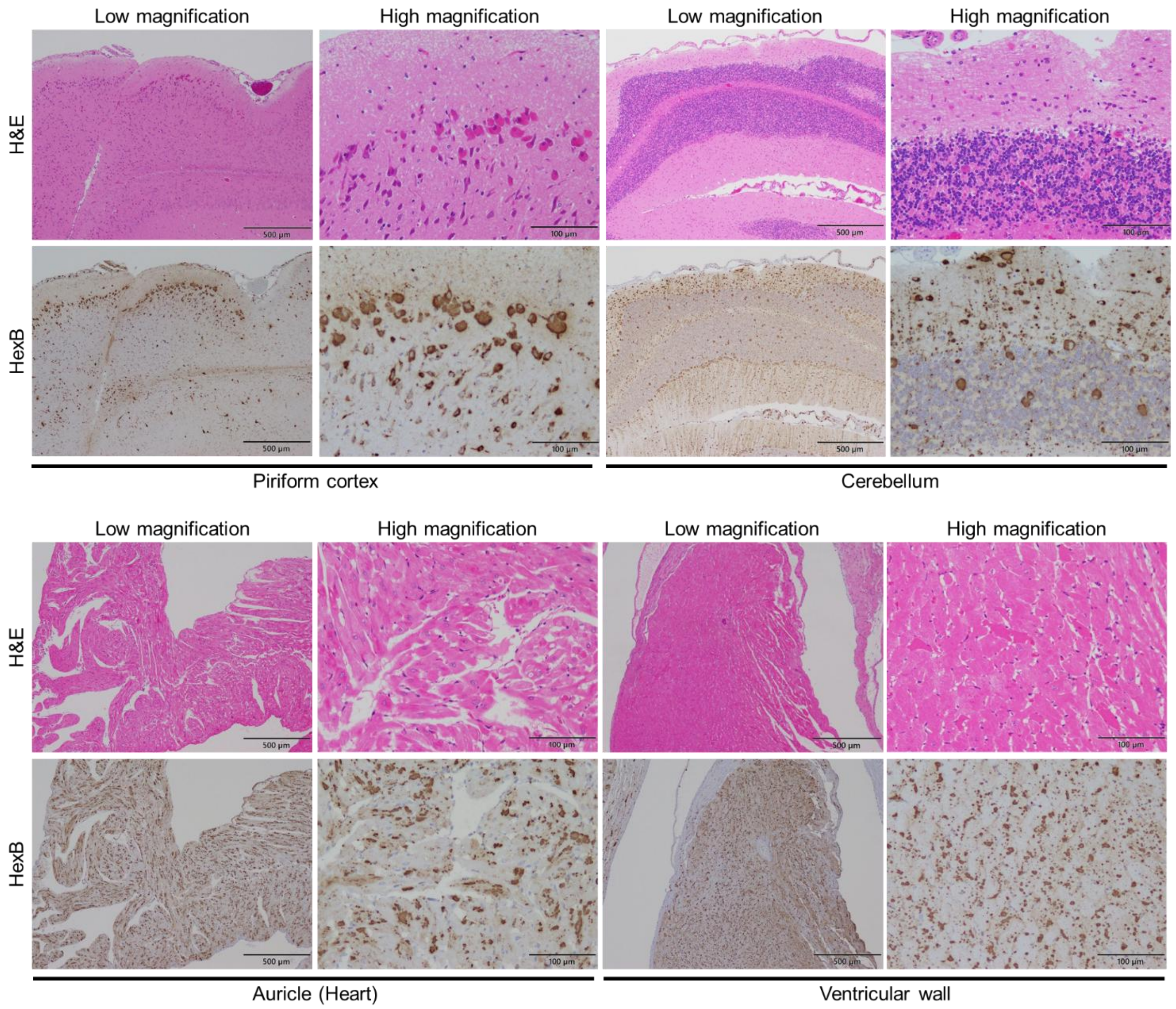


Fig. S6. H&E staining and immunohistochemical analysis of wild-type rat tissues following *i.t.* administration of the AAV9/3-*modHEXB*. Representative images of the high-dose group ($9.9 \times 10^{11}$ vg/g brain) are shown. Scale bars indicate 500 μm (low magnification) and 100 μm (high magnification), respectively.

Table S1. Quantification of fluorescent signal intensity

| | | WT | SD | SD + *modHEXB* |
|---|---|---|---|---|
| CD68/Nuclei | Cerebrum | 1.87 | 16.03 | 3.59 |
| | Hippocampus | 0.18 | 5.52 | 4.44 |
| | Cerebellum | 0.10 | 5.73 | 0.78 |
| NeuN/TUNEL/Nuclei | Cerebrum | 0.39 | 13.79 | 0.61 |
| | Hippocampus | 0.10 | 4.56 | 1.18 |
| | Cerebellum | 0.99 | 6.44 | 0.04 |

CD68-positive and NeuN/TUNEL-positive areas were quantified and normalized to the nuclear area, and the resulting values (%) are shown.

Table S3. Hematology and blood chemistry of non-human primates

| | Day0<br>n = 5 | Day28<br>n = 5 | Day84<br>n = 5 | Ref data<br>(Lab Anim Res 2019) | Ref data<br>(Animals 2023) |
|---|---|---|---|---|---|
| RBC ($10^4$/μL) | 594 ± 15 | 595 ± 16 | 592 ± 14 | 416–732 | 459–660 |
| WBC ($10^2$/μL) | 113.8 ± 19.1 | 132.0 ± 16.6 | 118.4 ± 18.0 | 30.4–184.0 | 62.7–254.8 |
| Ht (%) | 40.0 ± 1.1 | 40.8 ± 1.2 | 41.2 ± 1.0 | 31.6–57.2 | 33–42 |
| Hb (g/dL) | 11.7 ± 0.4 | 11.7 ± 0.3 | 11.5 ± 0.3 | 9.1–17.0 | |
| MCH (pg) | 19.6 ± 0.3 | 19.6 ± 0.3 | 19.4 ± 0.5 | 17.1–24.2 | |
| MCV (fL) | 67.3 ± 0.6 | 68.7 ± 1.0 | 69.6 ± 0.9 * | 63.8–82.9 | 59.2–76.4 |
| MCHC (g/dL) | 29.2 ± 0.2 | 28.6 ± 0.1 * | 27.8 ± 0.4 ** | 23.6–33.9 | |
| Plt ($10^4$/μL) | 32.3 ± 4.6 | 29.4 ± 2.7 | 27.7 ± 2.1 | 28.1–67.2 | 20.8–58.4 |
| W-SCR (%) | 39.5 ± 7.8 | 44.3 ± 6.6 | 44.2 ± 7.7 | | |
| W-LCR (%) | 60.5 ± 7.8 | 55.7± 6.6 | 55.8 ± 7.7 | | |
| W-SCC ($10^2$/μL) | 41.0 ± 9.0 | 58.4 ± 11.5 ** | 51.6 ± 12.1 | | |
| W-LCC ($10^2$/μL) | 72.8 ± 22.2 | 73.6 ± 12.6 | 66.8 ± 14.5 | | |
| RDW-SD (fL) | 24.0 ± 0.5 | 26.0 ± 0.8 * | 25.7 ± 0.7 * | | |
| PDW (fL) | 11.4 ± 0.9 | 11.1 ± 0.6 | 11.2 ± 0.6 | | 10.7–18.0 |
| MPV (fL) | 10.4 ± 0.5 | 10.2 ± 0.3 | 10.1 ± 0.4 | 7.6–16.3 | 9.6–13.4 |
| P-LCR (%) | 28.0 ± 4.3 | 26.9 ± 3.0 | 25.9 ± 3.2 | | 22.4–50.2 |
| AST (U/L) | 59.8 ± 2.3 | 74.4 ± 14.8 | 72.0 ± 26.2 | 13–106 | 23.9–91.4 |
| ALT (U/L) | 62.6 ± 6.1 | 112.6 ± 36.5 | 100.0 ± 37.7 | 1.0–56.0 | 11.8–77.8 |
| ALP (U/L) | 1809 ± 191 | 1903 ± 190 | 2110 ± 264 | 158–741 | 185–1327 |
| CPK (U/L) | 248 ± 32 | 395 ± 227 | 574 ± 202 | | |
| LDH (U/L) | 1285 ± 153 | 1123 ± 122 | 714 ± 211 | 31–467 | 327–1173 |
| γ-GTP (U/L) | 98 ± 20 | 102 ± 23 | 103 ± 24 | 1–199 | 38–157 |
| Glu. (mg/dL) | 36 ± 7 | 44 ± 9 | 48 ± 8 | 26–130 | |
| T.Cho. (mg/dL) | 105 ± 13 | 108 ± 12 | 102 ± 10 | 37–203 | 48–217 |
| F.Cho. (mg/dL) | 23.6 ± 2.9 | 24.6 ± 2.5 | 24.6 ± 2.4 | | |
| TG (mg/dL) | 36 ± 5 | 46 ± 6 * | 66 ± 11 ** | 9–213 | |
| TP (g/dL) | 6.9 ± 0.1 | 7.0 ± 0.1 | 7.1 ± 0.1 | 4.6–8.1 | 5.5–7.1 |
| Alb. (g/dL) | 4.4 ± 0.1 | 4.5 ± 0.1 | 4.4 ± 0.2 | 2.2–4.8 | 2.9–4.7 |
| A/G | 1.80 ± 0.08 | 1.75 ± 0.12 | 1.64 ± 0.12 | | |
| BUN (mg/dL) | 15.9 ± 1.6 | 18.1 ± 0.9 | 16.3 ± 0.8 | 7.5–28.0 | 13.7–30.8 |
| Fe (μg/dL) | 201 ± 12 | 211 ± 9 | 176 ± 29 | | 50–198 |
| CRP (mg/dL) | 0.0 ± 0.0 | 0.1 ± 0.0 ** | 0.3 ± 0.2 | | |
| Crea. (mg/dL) | 0.58 ± 0.02 | 0.56 ± 0.05 | 0.50 ± 0.00 * | 0.10–1.10 | 0.41–0.94 |
| T.Bil. (mg/dL) | 0.12 ± 0.02 | 0.14 ± 0.02 | 0.14 ± 0.04 | 0.10–2.30 | 0.01–0.22 |
| D.Bil. (mg/dL) | 0.00 ± 0.00 | 0.00 ± 0.00 | 0.00 ± 0.00 | 0.10–0.80 | |
| I.Bil. (mg/dL) | 0.12 ± 0.02 | 0.12 ± 0.02 | 0.14 ± 0.04 | | |
| Na (mEq/L) | 146.6 ± 1.5 | 146.6 ± 0.4 | 146.0 ± 1.6 | | 138.5–148.6 |
| K (mEq/L) | 4.7 ± 0.3 | 3.7 ± 0.1 * | 3.7 ± 0.1 * | | 3.1–4.4 |
| Cl (mEq/L) | 108.2 ± 0.7 | 107.0 ± 0.5 | 112.0 ± 1.4 | | 100.9–110.9 |
| P (mg/dL) | 5.1 ± 0.7 | 5.1 ± 0.5 | 4.9 ± 0.4 | 1.1–6.6 | 2.6–7.6 |
| Ca (mg/dL) | 9.5 ± 0.1 | 9.5 ± 0.1 | 9.7 ± 0.1 | 6.2–9.9 | 8.6–10.8 |

Values are the mean ± S.E.M.,

Paired t-test was performed to compare day 0 with day 28 and 84 [* $p$ <0.05, ** $p$ <0.01, *** $p$ <0.001].

Table S5. Histopathological findings in non-human primates

| Organ / Tissue | Subject No. | | | | |
|---|---|---|---|---|---|
| | **#1** | **#2** | **#3** | **#4** | **#5** |
| Cerebrum | +[a] | – | +[a] | – | – |
| Brainstem | – | – | – | – | – |
| Cerebellum | – | – | – | – | – |
| Cervical spinal cord | – | – | – | – | – |
| Thoracic spinal cord | – | – | – | – | – |
| Lumbar spinal cord | – | – | – | – | – |
| Heart | – | +[b] | +[c], [d] | – | +[d] |
| Lung | +[e] | +[f] | – | – | +[g], [f] |
| Liver | – | – | – | – | – |
| Gallbladder | – | – | – | – | Not examined |
| Kidney | – | – | – | – | – |
| Urinary bladder | – | – | – | – | – |
| Spleen | +[h] | – | +[h] | – | +[h] |
| Pancreas | – | – | – | – | – |
| Adrenal gland | – | – | – | – | – |
| Stomach | +[i] | – | – | +[i] | – |
| Small intestine | – | – | – | – | – |
| Large intestine | +[i] | – | – | +[i] | +[i] |
| Submandibular lymph node | – | +[h] | +[h], [j] | +[h] | – |
| Tonsil | – | – | – | – | – |
| Sciatic nerve | – | – | – | – | – |
| Uterus | – | – | – | – | – |
| Ovary | +[k] | +[k] | – | +[k] | – |
| Skin (dorsal) | – | – | – | – | – |
| Skeletal muscle (biceps brachii) | – | – | – | – | – |
| Skeletal muscle (femoral) | – | – | – | – | – |
| Eyeball | – | – | – | – | – |
| Inner ear | – | – | – | – | – |

－: no abnormality observed, +: mild, ++: moderate, +++: severe

Not examined: tissue not present on the histological section

(a) Gliosis, characterized by diffuse cortical microgliosis, accompanied by an increased frequency of neuronophagia and an increased number of eosinophilic neurons.

(b) Cardiomyocyte contraction band necrosis, focal, located in the interventricular septum and right ventricle adjacent to the coronary artery, without progression to cardiomyocyte liquefactive necrosis.

(c) Cardiomyocyte contraction band necrosis, focal and localized in the left ventricular wall, without progression to cardiomyocyte liquefactive necrosis.

(d) Mononuclear cell infiltration, consisting of multifocal minute lesions with focal cardiomyocyte degeneration/fragmentation.

(e) Foamy macrophage aggregation, multifocal and intra-alveolar.

(f) Mononuclear cell infiltration, multifocal, minute, and interstitial.

(g) Foamy macrophage aggregation, intra-alveolar.

(h) Lymphoid follicular hyperplasia, diffuse in distribution.

(i) Lymphoid follicular hyperplasia, localized within the mucosa.

(j) Extramedullary hematopoiesis, of granulocytic lineage.

(k) Mineral deposition, multifocal and focally distributed, scattered in the ovarian cortex.

Table S7. Organ weights

| | Male | | | Female | | |
|---|---|---|---|---|---|---|
| | Saline<br>n = 10 | AAV9/3-*modHEXB*, Low-dose<br>n = 10 | AAV9/3-*modHEXB*, High-dose<br>n = 9 | Saline<br>n = 9 | AAV9/3-*modHEXB*, Low-dose<br>n = 10 | AAV9/3-*modHEXB*, High-dose<br>n = 10 |
| Absolute weight | | | | | | |
| Brain (g) | 2.17±0.10 | 2.12 ± 0.09 | 2.23 ± 0.09 | 1.96 ± 0.09 | 1.98 ± 0.08 | 1.95 ± 0.14 |
| Pituitary gland (mg) | 12.4 ± 1.1 | 11.2 ± 1.5 | 12.2 ± 2.4 | 17.6 ± 5.1 | 17.0 ± 4.1 | 16.4 ± 3.8 |
| Thyroid glands (mg) 1) | 27.1 ± 3.7 | 23.7 ± 3.7 | 26.1 ± 2.3 | 20.2 ± 4.0 | 21.3 ± 5.0 | 20.5 ± 4.7 |
| Adrenal glands (mg) | 57 ± 8 | 51 ± 5 * | 65 ± 13 | 62 ± 8 | 69 ± 14 | 62 ± 8 |
| Heart (mg) | 1670 ± 165 | 1611 ± 259 | 1688 ± 233 | 956 ± 120 | 1023 ± 61 | 998 ± 131 |
| Lung (g) 2) | 1.66 ± 0.12 | 1.66 ± 0.15 | 1.72 ± 0.16 | 1.14 ± 0.11 | 1.21 ± 0.09 | 1.18 ± 0.11 |
| Thymus (mg) | 169 ± 38 | 193 ± 40 | 170 ± 49 | 143 ± 46 | 171 ± 28 | 165 ± 32 |
| Spleen (mg) | 917 ± 195 | 937 ± 194 | 964 ± 226 | 604 ± 94 | 627 ± 123 | 533 ± 132 |
| Submandibular glands (mg) 3) | 591 ± 76 | 642 ± 93 | 658 ± 137 | 392 ± 43 | 448 ± 70 | 380 ± 72 |
| Liver (g) | 18.12 ± 3.15 | 15.35 ± 4.21 | 16.14 ± 2.49 | 7.68 ± 1.06 | 8.43 ± 1.34 | 8.30 ± 1.40 |
| Kidneys (g) | 3.67 ± 0.40 | 3.34 ± 0.55 | 3.66 ± 0.43 | 1.94 ± 0.18 | 2.15 ± 0.28 | 2.05 ± 0.26 |
| Testes (g) | 3.60 ± 0.37 | 3.46 ± 0.22 | 3.53 ± 0.20 | - | - | - |
| Epididymides (mg) | 1409 ± 124 | 1412 ± 157 | 1331 ± 127 | - | - | - |
| Seminal vesicle (mg) | 1942 ± 357 | 1994 ± 230 | 2060 ± 273 | - | - | - |
| Prostate (mg) | 958 ± 176 | 888 ± 289 | 1114 ± 164 | - | - | - |
| Ovary (mg) | - | - | - | 105 ± 22 | 120 ± 15 | 127 ± 26 |
| Uterus (mg) | - | - | - | 694 ± 162 | 748 ± 164 | 690 ± 228 |
| Relative weight | | | | | | |
| Brain (g%) | 0.308 ± 0.027 | 0.338 ± 0.042 | 0.331 ± 0.039 | 0.611 ± 0.074 | 0.588 ± 0.080 | 0.575 ± 0.056 |
| Pituitary gland (mg%) | 1.74 ± 0.15 | 1.77 ± 0.18 | 1.81 ± 0.37 | 5.42 ± 1.48 | 5.04 ± 1.29 | 4.78 ± 0.91 |
| Thyroid glands (mg%) 1) | 3.82 ± 0.55 | 3.75 ± 0.67 | 3.87 ± 0.54 | 6.31 ± 1.62 | 6.30 ± 1.64 | 5.95 ± 1.07 |
| Adrenal glands (mg%) | 8.02 ± 1.11 | 8.06 ± 1.42 | 9.56 ± 1.92 | 19.26 ± 3.55 | 20.34 ± 4.55 | 18.26 ± 2.40 |
| Heart (mg%) | 235 ± 13 | 253 ± 20 | 248 ± 23 | 296 ± 34 | 303 ± 39 | 292 ± 28 |
| Lung (g%) 2) | 0.235 ± 0.025 | 0.262 ± 0.018 | 0.255 ± 0.028 | 0.356 ± 0.047 | 0.358 ± 0.055 | 0.347 ± 0.026 |
| Thymus (mg%) | 23.6 ± 4.2 | 30.6 ± 6.6 * | 24.9 ± 5.7 | 45.6 ± 18.4 | 51.0 ± 11.2 | 48.5 ± 9.8 |
| Spleen (mg%) | 129 ± 25 | 146 ± 14 | 143 ± 35 | 187 ± 28 | 186 ± 44 | 155 ± 32 |
| Submandibular glands (mg%) 3) | 84 ± 14 | 102 ± 17 * | 97 ± 19 | 122 ± 20 | 132 ± 23 | 111 ± 12 |
| Liver (g%) | 2.54 ± 0.28 | 2.38 ± 0.36 | 2.36 ± 0.15 | 2.37 ± 0.18 | 2.46 ± 0.20 | 2.42 ± 0.23 |
| Kidneys (g%) | 0.516 ± 0.036 | 0.523 ± 0.040 | 0.539 ± 0.045 | 0.603 ± 0.067 | 0.632 ± 0.070 | 0.597 ± 0.035 |
| Testes (g%) | 0.506 ± 0.029 | 0.551 ± 0.073 | 0.522 ± 0.044 | - | - | - |
| Epididymides (mg%) | 199 ± 23 | 223 ± 23 * | 197 ± 23 | - | - | - |
| Seminal vesicle (mg%) | 276 ± 61 | 315 ± 25 | 304 ± 42 | - | - | - |
| Prostate (mg%) | 136 ± 30 | 140 ± 50 | 166 ± 32 | - | - | - |
| Ovary (mg%) | - | - | - | 32.8 ± 8.0 | 35.4 ± 5.5 | 37.2 ± 6.0 |
| Uterus (mg%) | - | - | - | 217 ± 63 | 224 ± 68 | 201 ± 56 |
| Body weight (g%) | 711 ± 61 | 638 ± 93 | 680 ± 71 | 324 ± 37 | 343 ± 52 | 344 ± 50 |

Values are the mean ± S.D.

1) with parathyroid glands, 2) with bronchus, 3) with sublingual glands

Dunnett's/Steel's test was performed to compare the Control (Saline) group with each AAV9/3-*modHEXB* group [* $p$ <0.05].

Table S8. Ophthalmologic examination

| | Male | | | Female | | |
|---|---|---|---|---|---|---|
| | Saline<br>n = 5 | AAV9/3-*modHEXB*, Low-dose<br>n = 5 | AAV9/3-*modHEXB*, High-dose<br>n = 5 | Saline<br>n = 5 | AAV9/3-*modHEXB*, Low-dose<br>n = 5 | AAV9/3-*modHEXB*, High-dose<br>n = 5 |
| Appearance | 0 | 0 | 0 | 0 | 0 | 0 |
| Eyelid | 0 | 0 | 0 | 0 | 0 | 0 |
| Conjunctiva | 0 | 0 | 0 | 0 | 0 | 0 |
| Cornea | 0 | 0 | 0 | 0 | 0 | 0 |
| Sclera | 0 | 0 | 0 | 0 | 0 | 0 |
| Anterior chamber | 0 | 0 | 0 | 0 | 0 | 0 |
| Iris | 0 | 0 | 0 | 0 | 0 | 0 |
| Lens | 0 | 0 | 0 | 0 | 0 | 0 |
| Vitreous | 0 | 0 | 0 | 0 | 0 | 0 |
| Fundus: optic disc, retina, choroid | 0 | 0 | 0 | 0 | 0 | 0 |

Numerals represent the number of animals with the findings.

Table S9. Hematology

| | Male | | | Female | | |
|---|---|---|---|---|---|---|
| | Saline<br>n = 10 | AAV9/3-*modHEXB*, Low-dose<br>n = 10 | AAV9/3-*modHEXB*, High-dose<br>n = 9 | Saline<br>n = 9 | AAV9/3-*modHEXB*, Low-dose<br>n = 10 | AAV9/3-*modHEXB*, High-dose<br>n = 10 |
| RBC ($10^4$/μL) | 899 ± 22 | 908 ± 56 | 871 ± 77 | 794 ± 80 | 808 ± 67 | 807 ± 32 |
| WBC ($10^2$/μL) | 90.1 ± 21.7 | 118.2 ± 37.2 | 91.4 ± 36.1 | 66.8 ± 34.4 | 61.0 ± 28.4 | 56.8 ± 16.1 |
| Ht (%) | 42.6 ± 1.8 | 41.8 ± 2.0 | 41.5 ± 3.5 | 41.7 ± 2.9 | 40.9 ± 1.5 | 41.5 ± 1.4 |
| Hb (g/dL) | 15.7 ± 0.7 | 15.6 ± 0.8 | 15.3 ± 1.7 | 15.2 ± 1.4 | 15.1 ± 0.7 | 15.4 ± 0.5 |
| MCH (pg) | 17.5 ± 0.7 | 17.2 ± 0.7 | 17.5 ± 0.6 | 19.2 ± 0.6 | 18.7 ± 0.9 | 19.1 ± 0.4 |
| MCV (fL) | 47.4 ± 1.8 | 46.2 ± 2.6 | 47.7 ± 1.2 | 52.7 ± 2.5 | 50.9 ± 3.6 | 51.4 ± 1.6 |
| MCHC (g/dL) | 36.9 ± 0.5 | 37.3 ± 0.7 | 36.6 ± 1.6 | 36.4 ± 1.0 | 36.8 ± 0.9 | 37.1 ± 0.5 |
| Ret (%) | 3.79 ± 0.55 | 3.41 ± 1.13 | 4.96 ± 3.79 | 4.62 ± 4.12 | 3.93 ± 1.54 | 3.29 ± 0.51 |
| Ret ($10^4$/μL) | 34.01 ± 4.46 | 30.58 ± 8.36 | 40.73 ± 23.09 | 34.12 ± 23.25 | 30.96 ± 9.18 | 26.46 ± 3.54 |
| Plt ($10^4$/μL) | 126.0 ± 10.9 | 144.0 ± 16.1 * | 138.5 ± 14.3 | 113.0 ± 15.1 | 116.1 ± 6.3 | 111.9 ± 13.6 |
| PT (s) | 14.7 ± 1.3 | 16.1 ± 1.7 | 14.7 ± 1.5 | 12.2 ± 0.6 | 12.5 ± 0.6 | 12.2 ± 0.3 |
| APTT (s) | 20.4 ± 2.2 | 21.8 ± 2.3 | 20.6 ± 1.9 | 17.1 ± 1.0 | 17.1 ± 0.6 | 17.0 ± 0.6 |
| Differential leukocyte counts | | | | | | |
| Baso (%) | 0.0 ± 0.1 | 0.1 ± 0.0 | 0.1 ± 0.1 | 0.0 ± 0.0 | 0.0 ± 0.1 | 0.0 ± 0.0 |
| Eosi (%) | 1.8 ± 0.5 | 1.8 ± 0.6 | 1.9 ± 0.5 | 1.6 ± 0.4 | 1.7 ± 1.3 | 1.8 ± 0.7 |
| Neut (%) | 26.5 ± 10.3 | 29.8 ± 12.8 | 28.4 ± 4.8 | 26.0 ± 9.5 | 25.3 ± 10.9 | 22.7 ± 4.3 |
| Lymp (%) | 66.1 ± 10.5 | 63.6 ± 12.9 | 63.8 ± 5.5 | 68.4 ± 10.3 | 68.6 ± 11.2 | 71.2 ± 4.0 |
| Mono (%) | 5.6 ± 0.8 | 4.8 ± 1.4 | 5.8 ± 1.3 | 3.9 ± 1.3 | 4.3 ± 1.5 | 4.4 ± 0.9 |
| Baso ($10^2$/μL) | 0.0 ± 0.1 | 0.1 ± 0.0 | 0.1 ± 0.1 | 0.0 ± 0.0 | 0.0 ± 0.1 | 0.0 ± 0.0 |
| Eosi ($10^2$/μL) | 1.6 ± 0.5 | 2.1 ± 0.6 | 1.8 ± 1.0 | 1.0 ± 0.3 | 1.0 ± 0.6 | 1.0 ± 0.5 |
| Neut ($10^2$/μL) | 25.2 ± 15.7 | 37.8 ± 27.8 | 27.1 ± 15.9 | 19.7 ± 17.4 | 16.3 ± 10.5 | 12.9 ± 4.6 |
| Lymp ($10^2$/μL) | 58.3 ± 11.7 | 72.7 ± 18.4 | 57.1 ± 18.0 | 43.3 ± 16.3 | 41.1 ± 20.0 | 40.4 ± 11.6 |
| Mono ($10^2$/μL) | 5.0 ± 1.3 | 5.6 ± 2.1 | 5.3 ± 2.4 | 2.7 ± 1.7 | 2.6 ± 1.1 | 2.4 ± 0.5 |

Values are the mean ± S.D.

Dunnett's/Steel's test was performed to compare the Control (Saline) group with each AAV9/3-*modHEXB* group [* $p$ <0.05].

Table S10. Blood chemistry

| | Male | | | Female | | |
|---|---|---|---|---|---|---|
| | Saline | AAV9/3-*modHEXB*, Low-dose | AAV9/3-*modHEXB*, High-dose | Saline | AAV9/3-*modHEXB*, Low-dose | AAV9/3-*modHEXB*, High-dose |
| | n = 10 | n = 10 | n = 9 | n = 9 | n = 10 | n = 10 |
| AST (U/L) | 70.8 ± 16.2 | 71.3 ± 7.0 | 83.2 ± 9.7 | 171.4 ± 288.7 | 102.7 ± 26.3 | 138.6 ± 86.7 |
| ALT (U/L) | 27.9 ± 5.7 | 25.1 ± 4.5 | 26.8 ± 4.5 | 73.8 ± 137.4 | 30.6 ± 7.5 | 56.4 ± 47.8 |
| ALP (U/L) | 71 ± 16 | 67 ± 14 | 63 ± 16 | 28 ± 9 | 30 ± 13 | 25 ± 6 |
| LDH (U/L) | 76 ± 22 | 63 ± 14 | 70 ± 9 | 90 ± 73 | 88 ± 32 | 102 ± 61 |
| γ-GTP (U/L) | 0.5 ± 0.2 | 0.3 ± 0.1 | 0.4 ± 0.2 | 0.5 ± 0.2 | 0.6 ± 0.3 | 0.6 ± 0.3 |
| Glu. (mg/dL) | 179 ± 30 | 158 ± 27 | 153 ± 18 | 135 ± 16 | 138 ± 21 | 146 ± 14 |
| T.Cho. (mg/dL) | 81 ± 15 | 75 ± 21 | 76 ± 17 | 89 ± 22 | 79 ± 19 | 85 ± 9 |
| TG (mg/dL) | 74 ± 26 | 72 ± 44 | 73 ± 52 | 33 ± 25 | 32 ± 20 | 34 ± 20 |
| PL (mg/dL) | 114 ± 17 | 111 ± 27 | 112 ± 24 | 168 ± 43 | 148 ± 35 | 161 ± 14 |
| TP (g/dL) | 6.3 ± 0.4 | 6.3 ± 0.4 | 6.3 ± 0.3 | 6.8 ± 0.4 | 6.7 ± 0.5 | 6.8 ± 0.3 |
| Alb. (g/dL) | 2.2 ± 0.1 | 2.2 ± 0.2 | 2.1 ± 0.2 | 2.8 ± 0.3 | 2.5 ± 0.5 | 2.8 ± 0.2 |
| A/G | 0.55 ± 0.07 | 0.53 ± 0.06 | 0.51 ± 0.06 | 0.73 ± 0.08 | 0.61 ± 0.13 * | 0.72 ± 0.07 |
| BUN (mg/dL) | 12 ± 2 | 13 ± 2 | 15 ± 3 | 14 ± 1 | 15 ± 3 | 14 ± 3 |
| Crea. (mg/dL) | 0.28 ± 0.04 | 0.29 ± 0.04 | 0.29 ± 0.05 | 0.32 ± 0.04 | 0.31 ± 0.04 | 0.32 ± 0.06 |
| T.Bil. (mg/dL) | 0.07 ± 0.01 | 0.06 ± 0.01 | 0.06 ± 0.02 | 0.09 ± 0.05 | 0.07 ± 0.02 | 0.09 ± 0.02 |
| Na (mEq/L) | 143 ± 1 | 144 ± 1 | 144 ± 1 | 142 ± 1 | 142 ± 1 | 142 ± 1 |
| K (mEq/L) | 4.6 ± 0.3 | 4.5 ± 0.3 | 4.7 ± 0.3 | 3.9 ± 0.3 | 3.9 ± 0.3 | 3.8 ± 0.3 |
| Cl (mEq/L) | 108 ± 1 | 108 ± 2 | 109 ± 2 | 110 ± 1 | 109 ± 3 | 109 ± 1 |
| P (mg/dL) | 5.6 ± 0.5 | 5.5 ± 0.7 | 5.7 ± 0.6 | 4.4 ± 0.7 | 4.9 ± 0.6 | 4.6 ± 0.7 |
| Ca (mg/dL) | 10.1 ± 0.4 | 9.8 ± 0.4 | 9.8 ± 0.6 | 9.9 ± 0.4 | 10.0 ± 0.4 | 10.1 ± 0.2 |

Values are the mean ± S.D.

Dunnett's/Steel's test was performed to compare the Control (Saline) group with each AAV9/3-*modHEXB* group [* $p$ <0.05].

Table S11. Urinalysis

| | | Male | | | Female | | |
|---|---|---|---|---|---|---|---|
| | | Saline n = 5 | AAV9/3-*modHEXB*, Low-dose n = 5 | AAV9/3-*modHEXB*, High-dose n = 5 | Saline n = 5 | AAV9/3-*modHEXB*, Low-dose n = 5 | AAV9/3-*modHEXB*, High-dose n = 5 |
| Color | Yellow | 0 | 2 | 0 | 2 | 0 | 0 |
| | Pale yellow | 5 | 3 | 3 | 3 | 5 | 5 |
| | Achromatic urine | 0 | 0 | 2 | 0 | 0 | 0 |
| pH | 6.5 | 0 | 0 | 0 | 0 | 1 | 0 |
| | 7.5 | 0 | 0 | 0 | 0 | 0 | 1 |
| | 8.0 | 2 | 1 | 1 | 1 | 2 | 1 |
| | 8.5 | 3 | 4 | 4 | 4 | 2 | 3 |
| Protein | Negative | 0 | 2 | 1 | 3 | 4 | 4 |
| | Trace | 4 | 1 | 2 | 1 | 1 | 1 |
| | 30 mg/dL | 1 | 2 | 1 | 1 | 0 | 0 |
| | 100 mg/dL | 0 | 0 | 1 | 0 | 0 | 0 |
| Glucose | Negative | 5 | 5 | 5 | 5 | 5 | 5 |
| Ketone | Negative | 2 | 2 | 2 | 5 | 4 | 5 |
| | 5 mg/dL | 2 | 2 | 2 | 0 | 1 | 0 |
| | 15 mg/dL | 1 | 1 | 1 | 0 | 0 | 0 |
| Urobilinogen | 0.1–1 Ehrlich/dL | 5 | 5 | 5 | 5 | 5 | 5 |
| Bilirubin | Negative | 5 | 5 | 5 | 5 | 5 | 5 |
| Occult blood | Negative | 5 | 5 | 3 | 4 | 4 | 4 |
| | Trace | 0 | 0 | 2 | 0 | 1 | 1 |
| | Medium | 0 | 0 | 0 | 1 | 0 | 0 |
| Urinary sediments | | | | | | | |
| RBC | 0 cell/field | 5 | 5 | 5 | 4 | 5 | 5 |
| | <5 cells/field | 0 | 0 | 0 | 1 | 0 | 0 |
| WBC | 0 cell/field | 2 | 3 | 3 | 2 | 5 | 3 |
| | <6 cells/field | 3 | 2 | 2 | 3 | 0 | 2 |
| Epithelial cell | <6 (>0) cells/field | 5 | 5 | 5 | 5 | 5 | 5 |
| Cast | Negative | 5 | 5 | 5 | 5 | 5 | 5 |
| Crystal | Negative | 3 | 3 | 3 | 3 | 3 | 5 |
| | AMP | 2 | 2 | 2 | 2 | 2 | 0 |
| Others | Negative | 5 | 5 | 5 | 5 | 5 | 5 |
| Volume (mL) | | 15.8 ± 5.9 | 13.4 ± 4.7 | 28.4 ± 17.5 | 14.1 ± 4.9 | 20.2 ± 5.9 | 17.1 ± 5.2 |
| Specific gravity | | 1.047 ± 0.010 | 1.051 ± 0.013 | 1.042 ± 0.022 | 1.040 ± 0.007 | 1.033 ± 0.008 | 1.039 ± 0.012 |
| Electrolyte concentration | | | | | | | |
| Na (mEq/L) | | 68 ± 26 | 81 ± 34 | 80 ± 52 | 60 ± 23 | 63 ± 11 | 75 ± 28 |
| K (mEq/L) | | 202.3 ± 44.3 | 218.9 ± 60.5 | 198.2 ± 111.0 | 179.9 ± 44.7 | 151.6 ± 34.7 | 187.2 ± 60.5 |
| Cl (mEq/L) | | 109± 35 | 121± 39 | 120± 80 | 108 ± 33 | 92 ± 16 | 111 ± 38 |
| Total excretion | | | | | | | |
| Na (mg/day) | | 25 ± 13 | 25 ± 12 | 36 ± 11 | 19 ± 7 | 29 ± 6 * | 27 ± 3 |
| K (mg/day) | | 121 ± 43 | 108 ± 29 | 160 ± 36 | 93 ± 18 | 114 ± 11 | 116 ± 10 * |
| Cl (mg/day) | | 61 ± 28 | 55 ± 21 | 82 ± 25 | 51 ± 14 | 64 ± 11 | 62 ± 3 |

Numerals represent the number of animals with the findings. Values are the mean ± S.D.

AMP: Ammonium magnesium phosphate

Dunnett's/Steel's test was performed to compare the Control (Saline) group with each AAV9/3-*modHEXB* group [* $p$ <0.05].

Table S12. Gross pathological findings

| | Male | | | Female | | |
|---|---|---|---|---|---|---|
| | Saline<br>n = 10 | AAV9/3-*modHEXB*, Low-dose<br>n = 10 | AAV9/3-*modHEXB*, High-dose<br>n = 9 | Saline<br>n = 9 | AAV9/3-*modHEXB*, Low-dose<br>n = 10 | AAV9/3-*modHEXB*, High-dose<br>n = 10 |
| Brain (Ventricular dilatation) | 0 | 1 | 0 | 0 | 0 | 0 |
| Lung (Mottled grayish-white color, scattered, all lobules) | 1 | 0 | 0 | 0 | 0 | 0 |
| Hind limb (Lost, left, all fingers) | 0 | 0 | 1 | 0 | 0 | 0 |
| Spleen (Large size) | 0 | 0 | 1 | 0 | 0 | 0 |

Numerals represent the number of animals with the findings.

Table S14. Anti-AAV9/3 antibody analysis

| Group | Sex | Absorbance | Judgement |
|---|---|---|---|
| Saline (n = 5) | Male | 0.032 | Negative |
| | | 0.030 | Negative |
| | | 0.028 | Negative |
| | | 0.040 | Negative |
| | | 0.029 | Negative |
| | Female | 0.049 | Negative |
| | | 0.025 | Negative |
| | | 0.027 | Negative |
| | | 0.043 | Negative |
| | | 0.027 | Negative |
| AAV9/3-*modHEXB*, Low-dose (n = 5) | Male | 3.754 | Positive |
| | | 3.741 | Positive |
| | | 3.803 | Positive |
| | | 3.864 | Positive |
| | | 3.728 | Positive |
| | Female | 3.152 | Positive |
| | | 1.450 | Positive |
| | | 3.775 | Positive |
| | | 3.777 | Positive |
| | | 3.790 | Positive |
| AAV9/3-*modHEXB*, High-dose (n = 5) | Male | 2.793 | Positive |
| | | 3.808 | Positive |
| | | 3.657 | Positive |
| | | 3.072 | Positive |
| | | 3.763 | Positive |
| | Female | 3.781 | Positive |
| | | 3.671 | Positive |
| | | 3.749 | Positive |
| | | 3.753 | Positive |
| | | 3.806 | Positive |

Absorbance of Negative Control sample: 0.025
Absorbance of Positive Control sample: 0.499
Cut point: 0.058

## Supplemental references


[S1] Koo BS, Lee DH, Kang P, Jeong KJ, Lee S, Kim K, Lee Y, Huh JW, Kim YH, Park SJ, Jin YB, Kim SU, Kim JS, Son Y, Lee SR. Reference values of hematological and biochemical parameters in young-adult cynomolgus monkey (*Macaca fascicularis*) and rhesus monkey (*Macaca mulatta*) anesthetized with ketamine hydrochloride. *Lab. Anim. Res.* **35**, 7 (2019). https://doi.org/10.1186/s42826-019-0006-0

[S2] Bakker J, Maaskant A, Wegman M, Zijlmans DGM, Hage P, Langermans JAM, Remarque EJ. Reference intervals and percentiles for hematologic and serum biochemical values in captive bred rhesus (*Macaca mulatta*) and cynomolgus macaques (*Macaca fascicularis*). *Animals (Basel)*. **13**, 445 (2023). https://doi.org/10.3390/ani13030445
