# Supplementary material for: AAV-based gene therapy with modified HEXB confers lasting therapeutic benefits in GM2 gangliosidosis models
Source: Cell Rep Med. 2026 Apr 22;7(5):102762. doi: 10.1016/j.xcrm.2026.102762 (PMC13198282; doi:10.1016/j.xcrm.2026.102762)
Supplement: Document S6. Article plus supplemental information [file mmc6.pdf]

Cell Reports
Medicine

Article

AAV-based gene therapy with modified *HEXB* confers lasting therapeutic benefits in GM2 gangliosidosis models

Graphical abstract

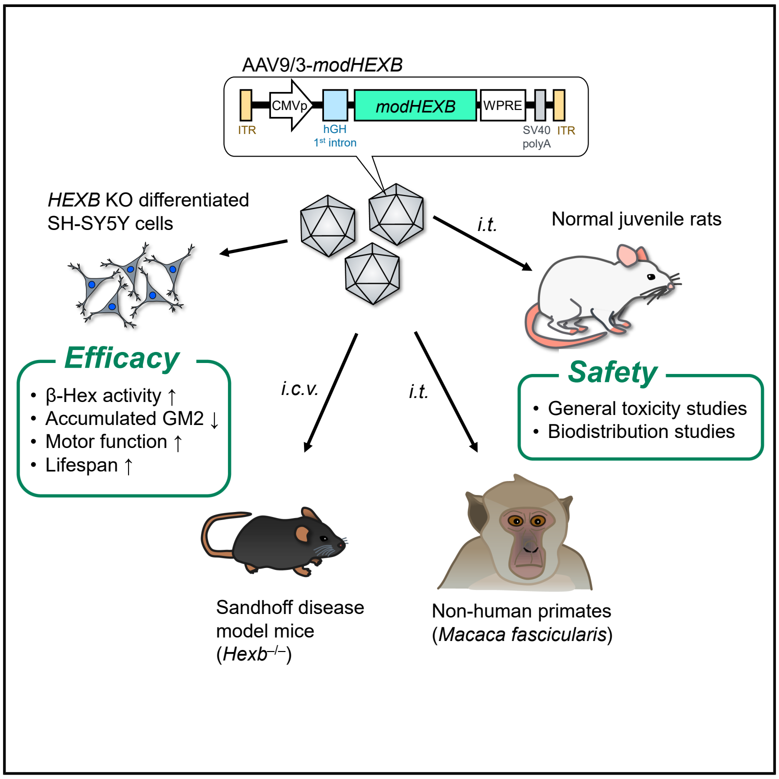

Highlights

- A modified *HEXB* gene was delivered using an adeno-associated virus vector
- Gene transfer reduced substrate accumulation and neuroinflammation in disease models
- Gene therapy markedly improved motor function and survival in affected mice
- Intrathecal delivery showed broad distribution and safety in preclinical species

Authors

Keisuke Kitakaze, Yukiya Ohnishi, Daisuke Tsuji, ..., Naomi Takino, Shin-ichi Muramatsu, Kohji Itoh

Correspondence

kitoh@tokushima-u.ac.jp

In brief

Kitakaze et al. establish proof of concept for treating GM2 gangliosidosis using a single gene therapy encoding a modified lysosomal enzyme. By simplifying gene design and demonstrating efficacy and safety across species, the study provides a foundation for clinical translation in neurodegenerative lysosomal disorders.

Kitakaze et al., 2026, Cell Reports Medicine *7*, 102762
May 19, 2026 © 2026 The Author(s). Published by Elsevier Inc.
https://doi.org/10.1016/j.xcrm.2026.102762

CellPress

CellPress
OPEN ACCESS

Article

AAV-based gene therapy with modified *HEXB* confers lasting therapeutic benefits in GM2 gangliosidosis models

Keisuke Kitakaze,[1,9] Yukiya Ohnishi,[2,9] Daisuke Tsuji,[2,3,8] Ryosuke Watanabe,[2] Nijiho Kamori,[3] Yuko Katakai,[4] Hiroaki Shibata,[4] Sota Yoshizawa,[5] Mika Ito,[6] Naomi Takino,[6] Shin-ichi Muramatsu,[5,6] and Kohji Itoh[2,3,7,10,*]
[1]Department of Pharmacology, Kawasaki Medical School, 577 Matsushima, Kurashiki, Okayama 701-0192, Japan
[2]Department of Medicinal Biotechnology, Graduate School of Pharmaceutical Sciences, Tokushima University, 1-78-1 Sho-machi, Tokushima 770-8505, Japan
[3]Department of Medicinal Biotechnology, Faculty of Pharmaceutical Sciences, Tokushima University, 1-78-1 Sho-machi, Tokushima 770-8505, Japan
[4]The Corporation for Production and Research of Laboratory Primates, 1-16-2 Sakura, Tsukuba, Ibaraki 305-0003, Japan
[5]ONODERA GT Pharma, Inc., 3-25-22 Tonomachi, Kawasaki, Kanagawa 210-0821, Japan
[6]Division of Neurological Gene Therapy, Center for Open Innovation, Jichi Medical University, 3311-1 Yakushiji, Shimotsuke, Tochigi 329-0498, Japan
[7]Division of Pediatrics, Jichi Medical University, 3311-1 Yakushiji, Shimotsuke, Tochigi 329-0498, Japan
[8]Present address: Department of Pharmacy, Faculty of Pharmacy, Yasuda Women's University, 6-13-1 Yasuhigashi, Asaminami-ku, Hiroshima 731-0153, Japan
[9]These authors contributed equally
[10]Lead contact
*Correspondence: kitoh@tokushima-u.ac.jp
https://doi.org/10.1016/j.xcrm.2026.102762

SUMMARY

GM2 gangliosidoses, including Tay-Sachs (TSD) and Sandhoff (SD) diseases, are lysosomal storage disorders with neurological manifestations caused by the excessive accumulation of GM2 ganglioside due to the deficiency of the β-hexosaminidase A (HexA). Although gene therapy approaches are underway, concerns regarding efficacy and safety remain. Here, we evaluate a tyrosine-mutant adeno-associated virus serotype 9 (AAV9/3) vector encoding modified *HEXB* (*modHEXB*) wherein nine amino acid residues are substituted from *HEXA*. The intracerebroventricular administration of AAV9/3-*modHEXB* in SD mice results in modHexB expression in the brain, reduces GM2 accumulation, and attenuates neuroinflammation. Furthermore, AAV9/3-*modHEXB* rescues motor function, and longer lifespan in SD mice. In addition, intrathecal administration in non-human primates and rats demonstrates broad biodistribution and an overall favorable safety profile. These findings support the translational potential of AAV9/3-*modHEXB* as a gene therapy approach for TSD and SD.

INTRODUCTION

The physiological degradation of GM2 ganglioside (GM2) is concurrently regulated by the lysosomal enzyme β-hexosaminidase A (HexA) and its cofactor, the GM2-activating protein (GM2A).[1] HexA is a heterodimer composed of α and β subunits, encoded by *HEXA* (located on 15q23) and *HEXB* (located on 5q13.3), respectively. GM2 gangliosidoses, including Tay-Sachs disease (TSD) and Sandhoff disease (SD), are lysosomal storage disorders (LSDs) classified as GM2 gangliosidoses with neurological manifestations caused by recessive mutations in *HEXA* and *HEXB*, respectively, resulting in HexA deficiency and excessive accumulation of GM2 in diverse organs, particularly in the brain.[2] Currently, there are no approved therapies for these diseases.

Enzyme replacement therapy (ERT) has been clinically applied to treat several LSDs.[3] In ERT, the recombinant human lysosomal enzymes carrying mannose 6-phosphate (M6P)-containing *N*-glycans are incorporated into the cell via the cation-independent M6P receptor (CI-M6PR).[4,5] However, intravenously administered recombinant enzymes cannot cross the blood-brain barrier and have little efficacy against neurological symptoms. Therefore, recombinant enzymes have been administered intracerebroventricularly (i.c.v.) to patients with specific neurological LSDs, such as neuroceroid lipofuscinosis type 2[6] and mucopolysaccharidosis type II.[7] Gene therapy through i.c.v. or intrathecal (i.t.) administration of adeno-associated virus (AAV) vectors has been developed for neurodegenerative LSDs and has been well tolerated in clinical trials.[8,9] AAV vectors can be widely distributed to neuronal cells through a single administration into the cerebrospinal fluid. The therapeutic gene is delivered to the nucleus, where it is episomally expressed over an extended period. The recombinant lysosomal enzymes overexpressed in neuronal cells are expected to have cross-correction;

 © 2026 The Author(s). Published by Elsevier Inc.
This is an open access article under the CC BY license (http://creativecommons.org/licenses/by/4.0/).

they are extracellularly secreted and taken up into surrounding cells via CI-M6PR.[10] Since a HexA activity of approximately 10%–15% of normal levels has been suggested as a minimal threshold to prevent GM2 accumulation under steady-state conditions,[11] higher levels of enzyme activity are likely required to reduce pre-existing substrate storage. Therefore, *in vivo* gene therapy using AAV vectors achieving robust Hex expression is believed to be therapeutically effective.

Currently, introducing *HEXA* and *HEXB* into the same cell for expressing the heterodimer HexA as gene therapy for TSD and SD is challenging. Applying a 1:1 ratio of two monocistronic AAVrh8 vectors carrying *HEXA* or *HEXB* has been evaluated in a single intrathalamic/i.t. co-administration to patients with infantile TSD.[12] While the safety of this approach was proven, HexA activity in the patient cerebrospinal fluid recovered from 0.5% to only 1.44% of that in healthy controls, possibly due to the difficulty of co-introducing the two types of AAV vectors in the same cells. Another problem is that two different vectors must be produced, which increases costs. To overcome these challenges, a bicistronic expression vector carrying *HEXB-P2A-HEXA* was developed,[13] and a clinical trial (phase 1/2) of a single i.t. administration of AAV9-*HEXB-P2A-HEXA* for patients with TSD or SD started in 2021 (https://clinicaltrials.gov/study/NCT04798235). While showing safety and efficacy, this clinical trial was early terminated primarily for financial reasons. Recently, a bidirectional promoter-driven AAV9 vector encoding both *HEXA* and *HEXB* has been developed.[14–16] Although these approaches are promising, alternative approaches are still needed because the efficiency of heterodimer formation is not high; the isozymes without GM2-degrading activity HexS (αα) and HexB (ββ) are also gene products of *HEXA* and *HEXB*.[13,14]

We previously reported the construction of a modified *HEXB* gene (*modHEXB*) wherein the amino acid residues in the human β-subunit were substituted for those in the α-subunit responsible for the anionic substrate recognition (DL 452–453 NR) and interaction with GM2A protein (RQNKLDS 312–318 GSEPSGT).[17] The i.c.v. administration of purified recombinant modHexB (β′β′ homodimer) decreased accumulated GM2, improved motor function, and prolonged lifespan in SD mice. In that study, two variants (modB and mod2B) were described; the construct used in the present study corresponds to mod2B, which showed a survival benefit *in vivo*, and is referred to here as modHexB. Furthermore, we previously developed an AAV9/3 vector in which two tyrosine residues in the capsid protein of AAV9 were replaced with phenylalanine to avoid ubiquitination-based degradation after infection in the host cell, as well as using the inverted repeat of AAV3 to improve transgene expression efficiency.[18]

In this study, we combined these previous advancements to construct the GT0005X (AAV9/3-*modHEXB*), which carries a cytomegalovirus (CMV) promoter and *modHEXB*, and investigated its therapeutic efficacy and safety in SD mice. Furthermore, we performed a single i.t. administration of non-Good Manufacturing Practice (GMP)- and GMP-grade AAV9/3-*modHEXB* to non-human primates and juvenile normal rats, respectively. Our findings provide a proof of concept for the pharmacodynamics and safety of AAV9/3-*modHEXB*. We proposed the *in vivo* gene therapy using *modHEXB* as an alternative approach to the use of two therapeutic genes, *HEXA* and *HEXB*.

RESULTS

AAV9/3-*modHEXB* restores β-Hex activity in cultured human neuronal cells

We constructed an AAV9/3 vector encoding *modHEXB* regulated by the ubiquitous CMV promoter of the AAV9/3 vector, with inclusion of the human growth hormone (hGH) intron to enhance transgene expression (Figure 1A). We examined modHexB expression in *HEXB*-knockout human neuroblastoma SH-SY5Y and neurons differentiated from induced pluripotent stem cells derived from patients with TSD. These cells lack HexA activity and accumulate GM2 in lysosomes.[19] The *modHEXB* transgene significantly restored β-Hex activity in cell extracts in a dose-dependent manner (Figures 1B and S1) and reduced the accumulation of GM2 (Figure 1C). We also observed β-Hex activity in the conditioned medium of *HEXB*-knockout SH-SY5Y following AAV9/3-*modHEXB* infection (Figure 1B). Consistent with these observations, we detected modHexB precursor and mature proteins in the conditioned medium (Figure 1D). Although the presence of extracellular modHexB does not by itself demonstrate functional cross-correction, it is compatible with the possibility that secreted modHexB precursor may be available for uptake by surrounding cells in a CI-M6PR-dependent manner, which is expected due to the cross-correction (Figure 1E). To further characterize the Hex isozymes produced following AAV9/3-*modHEXB* transduction, cell lysates from SH-SY5Y cells were subjected to anion-exchange chromatography. Immunoblot analysis revealed that the majority of modHexB was detected in the flow-through fractions, consistent with the formation of β′-β′ homodimers, whereas only trace amounts of α-β′ heterodimers were detected in the bound fractions (Figure S2). Furthermore, we observed significantly higher recovery of β-Hex activity with AAV9/3-*modHEXB* compared to that of AAV9/3-*HEXB-P2A-HEXA* bicistronic vector (Figure 1F).

i.c.v. administration of AAV9/3-*modHEXB* restores β-Hex activity in SD mice

SD mice (*Hexb*$^{-/-}$) exhibit progressive accumulation of GM2 in the brain[20,21] and serve as a useful model for developing therapeutic strategies, including *in vivo* gene therapy.[13,22,23] Dose selection for the mouse studies was guided by previously published AAV-based gene therapy studies using SD mice, in which therapeutic effects were reported at doses of approximately 2×10^{13} vector genome (vg)/kg[13] and 1.5–5 $\times 10^{13}$ vg/kg.[14] Based on these reports, we designed our dosing strategy within this range or below. We conducted the i.c.v. administration of AAV9/3-*modHEXB* to SD mice and measured the recovery of β-Hex activity (Figures 2A and 2B) across diverse cerebrospinal regions, including the cortex, hippocampus, thalamus, cerebellum, and spinal cord. The expression of modHexB was confirmed using anti-human HexA antibody (Figure 2C). The accumulation of GM2 in those regions decreased with the degree of recovery of β-Hex activity (Figures 2D–2F). Consistent with previous reports,[24] i.c.v. administration of AAV9/3-green fluorescent protein (GFP) to wild-type mice resulted in high GFP expression mainly in the parietal lobe, co-localizing with NeuN-positive neurons, reflecting the regional pattern of direct

CellPress
OPEN ACCESS

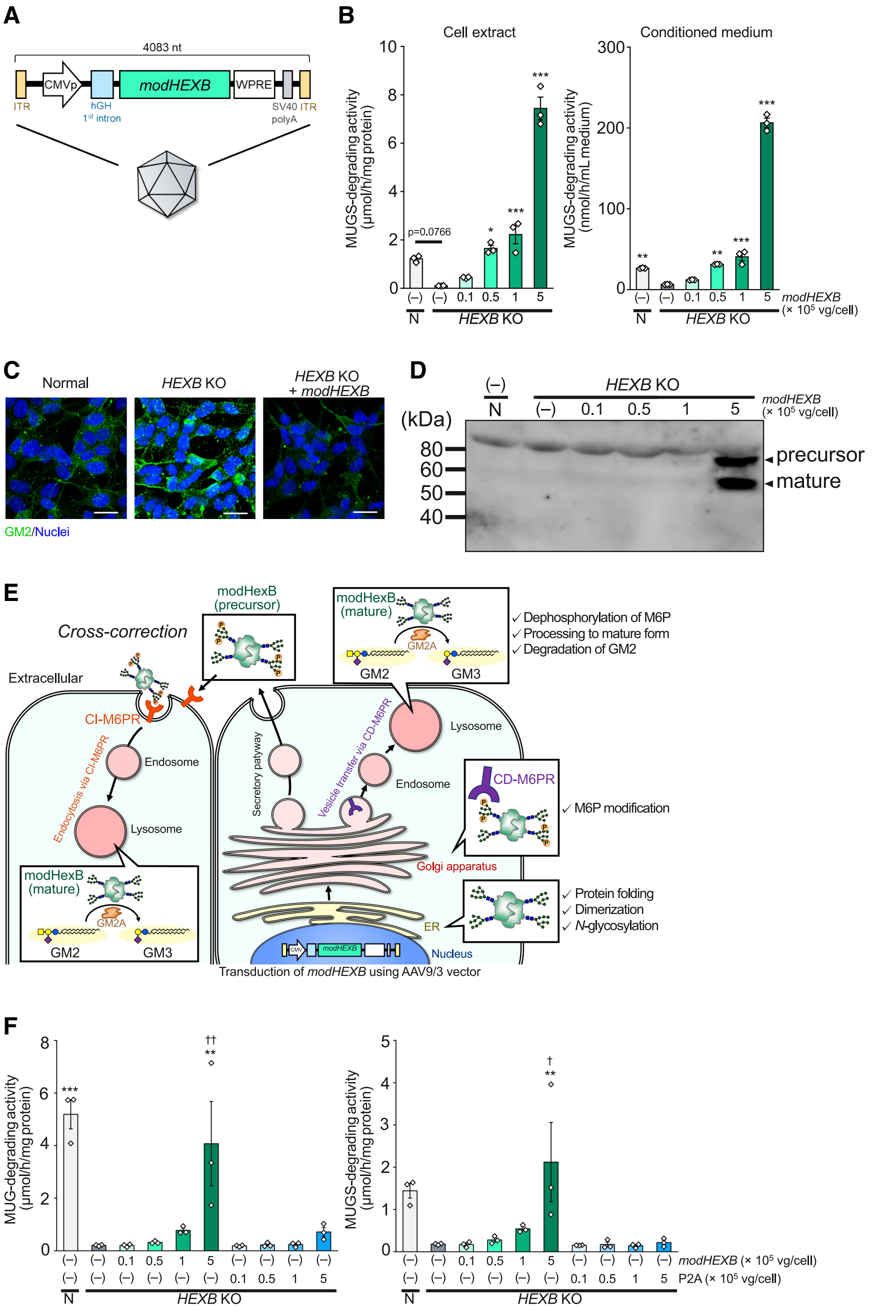

Figure 1. Restoration of β-Hex activity using AAV9/3-*modHEXB* in cultured human neuronal cells

(A) Schematic illustration of the AAV9/3-*modHEXB*. ITR: inverted terminal repeat, CMVp: cytomegalovirus promoter, WPRE: woodchuck hepatitis virus post-transcriptional regulatory element.

(B) Intracellular and extracellular MUGS-degrading β-Hex activity in *HEXB* KO SH-SY5Y cells after transduction with 0.1–5 × 10^5 vg/cell AAV9/3-*modHEXB*. Error bars show mean ± SEM (n = 3 biological replicates). One-way ANOVA with Tukey's test was conducted. *$p < 0.05$, **$p < 0.01$, ***$p < 0.001$ (vs. untreated *HEXB* KO cells). N: normal.

(C) Immunofluorescence staining of accumulated GM2 in *HEXB* KO SH-SY5Y cells after transduction with 5 × 10^5 vg/cell AAV9/3-*modHEXB*. Green: GM2, blue: nuclei. Scale bars, 20 μm.

(D) Conditioned medium was separated using SDS-PAGE, followed by immunoblotting with anti-HexA antibody. Each lane contained 10 μg of protein. N: normal.

(E) Schematic illustration of a proposed model for modHexB trafficking and potential cross-correction. Biosynthesis and post-translational modifications of modHexB proceed in the endoplasmic reticulum (ER) and Golgi apparatus in AAV9/3-*modHEXB*-transduced cells. The modHexB carrying M6P-containing *N*-glycans is transported to lysosomes via CD-M6PR, processed to the mature form, and degrades GM2 cooperatively with GM2A. In addition, overexpressed modHexB precursors are secreted, endocytosed via CI-M6PR to surrounding cells, and transported to lysosomes to degrade GM2.

(F) Intracellular MUG- and MUGS-degrading β-Hex activity in *HEXB* KO SH-SY5Y cells after transduction with 0.1–5 × 10^5 vg/cell AAV9/3-*modHEXB* (*modHEXB*) or *HEXB-P2A-HEXA* (P2A). Error bars show mean ± SEM (n = 3). One-way ANOVA with Tukey's test was conducted for each analysis. **$p < 0.01$, ***$p < 0.001$ (vs. untreated *HEXB* KO cells). $^{\dagger}p < 0.05$, $^{\dagger\dagger}p < 0.01$ (vs. P2A at the corresponding vector dose).

AAV transduction (Figure S3). In the SD mouse brain, neuronal inflammation is induced by the expression of macrophage inflammatory protein-1α (MIP-1α)[25] and microglial activation,[26] which are suggested to influence the pathogenesis of SD. Thus, we investigated whether AAV9/3-*modHEXB* affects these markers in SD mice. We observed that AAV9/3-*modHEXB* administration reduced the expression levels of MIP-1α and TUNEL-positive neuronal cell death, as well as suppressing the CD68-positive activated microglia (Figures 2G and 2H; Table S1). In addition, we evaluated immune responses against the transgene product and detected anti-modHexB antibodies in the plasma of treated SD mice (Figure S4). Although it is unclear whether these antibodies had a neutralizing effect or influenced the therapeutic efficacy, these data provide information on the immunogenicity of modHexB in this disease model.

i.c.v. administration of AAV9/3-*modHEXB* improves neurological signs in SD mice

SD mice develop neurological signs such as tremors, startle response, and motor dysfunction similar to those of patients with TSD and SD,[20,21] dying within approximately 4 months. We compared the therapeutic effects of a single i.c.v. administration of AAV9/3-*HEXB-P2A-HEXA* bicistronic vector or AAV9/3-*modHEXB* on 8-week-old SD mice. They exhibited progressive

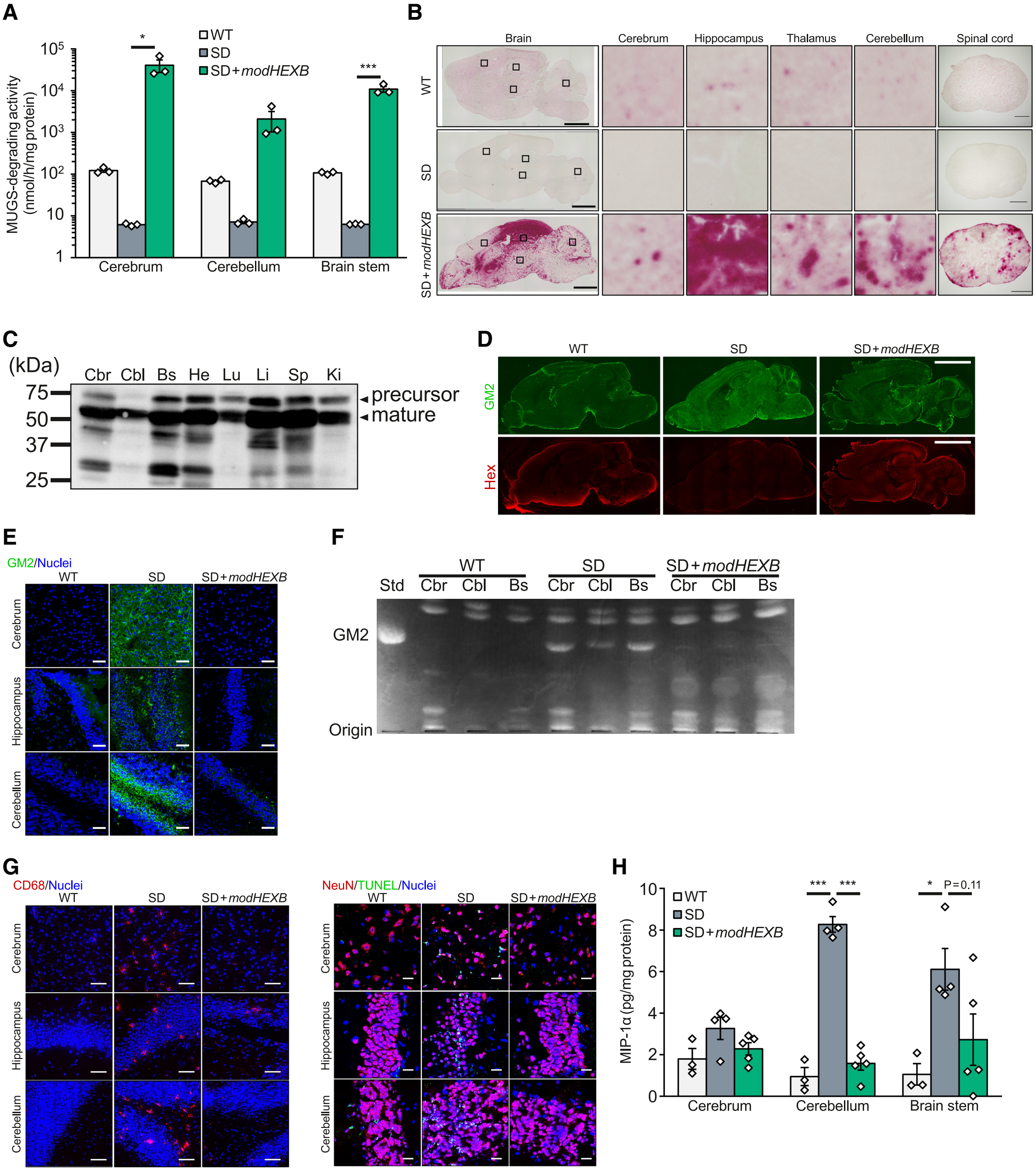

Figure 2. Restoration of β-Hex activity by i.c.v. administration of AAV9/3-*modHEXB* to SD mice

AAV9/3-*modHEXB* was administrated at a dose of 2.9 × 10^{13} vg/kg BW to 8-week-old SD mice and analyzed at age 16 weeks (A, B, and E–H) or 1.6 × 10^{13} vg/kg BW to 14-week-old SD mice and analyzed at age 15 weeks (C and D).

One-way ANOVA with Tukey's test was conducted (A and H). $^{*}p < 0.05$, $^{***}p < 0.001$.

(A) Recovery of MUGS-degrading β-Hex activity. Error bars show means ± SEM. WT, male = 1 and female = 2; SD, male = 1 and female = 2; AAV9/3-*modHEXB*, male = 2 and female = 1.

(B) *In situ* staining of β-Hex activity. Scale bars: 2 mm (whole area) and 0.5 mm (spinal cord).

(C) Tissue extracts from AAV9/3-*modHEXB*-treated SD mice were separated by SDS-PAGE, and modHexB protein was detected by immunoblotting with anti-HexA antibody. Each lane contained 10 μg of protein. The anti-HexA antibody is a polyclonal antibody that can also detect endogenous murine Hex subunits. Cbr: cerebrum, Cbl: cerebellum, Bs: brainstem, He: heart, Lu: lung, Li: liver, Sp: spleen, and Ki: kidney.

(D) Immunohistochemical analyses of GM2 and β-Hex in sagittal sections of the whole brain. Green: GM2, red: β-Hex. Scale bars, 3 mm.

(E) Immunohistochemical analyses of GM2 in the cerebrum, hippocampus, and cerebellum. Blue: nuclei, green: GM2. Scale bars: 50 μm.

(legend continued on next page)

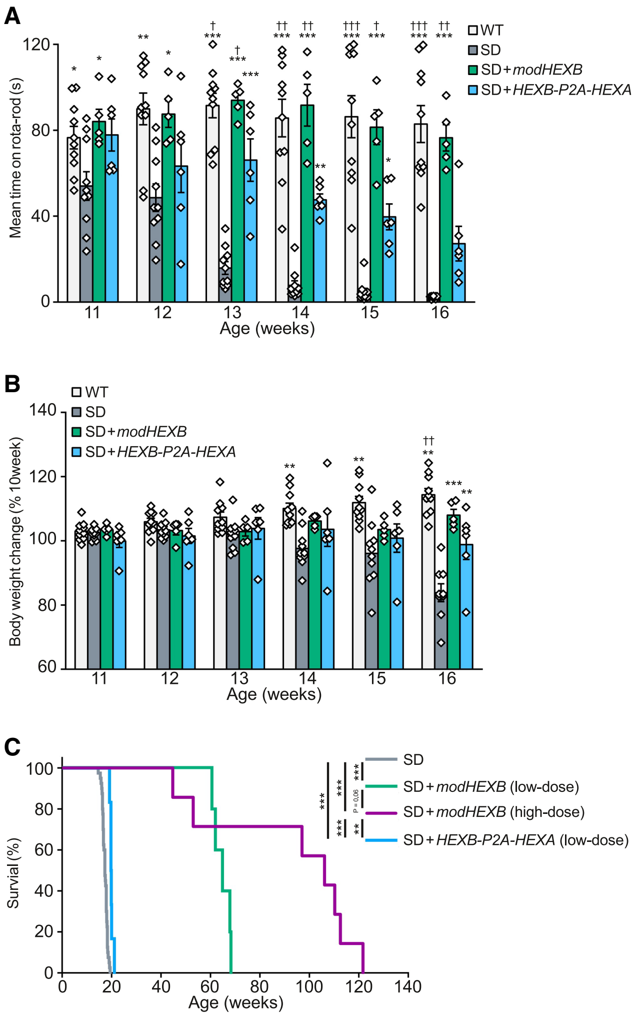

Figure 3. Improvement of neurological signs in SD mice by i.c.v. administration of AAV9/3-*modHEXB*

AAV9/3-*modHEXB* or -*HEXB-P2A-HEXA* was administrated at a dose of 5.8 × 10^{12} vg/kg BW to 8-week-old SD mice

(A) Evaluation of the motor dysfunction. Error bars show means ± SEM.

(B) Body weight change. Error bars show means ± SEM. WT, male = 7 and female = 3; SD, male = 3 and female = 7; AAV9/3-*modHEXB*, female = 5; AAV9/3-*HEXB-P2A-HEXA*, male = 6.

(C) Lifespan analysis. Untreated SD mice, male = 26 and female = 14; AAV9/3-*modHEXB* (5.8 × 10^{12} vg/kg BW), male = 1 and female = 4; AAV9/3-*modHEXB* (2.9 × 10^{13} vg/kg BW), male = 6 and female = 1; AAV9/3-*HEXB-P2A-HEXA* (5.8 × 10^{12} vg/kg BW), male = 6. One-way ANOVA with Tukey's test was conducted. $^{*}p < 0.05$, $^{**}p < 0.01$, $^{***}p < 0.001$ (vs. SD), $^{\dagger}p < 0.05$, $^{\dagger\dagger}p < 0.01$, $^{\dagger\dagger\dagger}p < 0.001$ (vs. P2A) (A and B). Log rank test, $^{**}p < 0.01$, $^{***}p < 0.001$ (C).

motor dysfunction and weight loss after 11 weeks of age, and the administration of *HEXB-P2A-HEXA* encoding bicistronic vector (5.8 × 10^{12} vg/kg body weight [BW]) partially improved motor function from 13 to 15 weeks of age. Conversely, administration of AAV9/3-*modHEXB* at 5.8 × 10^{12} vg/kg BW significantly improved motor function to levels similar to those of wild-type mice of age 11–16 weeks (Figure 3A). Preservation of body weight loss in the AAV9/3-*modHEXB*-treated group was modest and became apparent at later time points, without reaching statistical significance when compared with the AAV9/3-*HEXB-P2A-HEXA*-treated group (Figure 3B). The median lifespan of untreated SD mice was 121 days; herein, that of the bicistronic vector-treated group was modestly extended to 138 days. Thus, although the bicistronic vector conferred some functional benefits, it did not substantially alter overall disease progression or survival. Notably, in the AAV9/3-*modHEXB*-treated group administered at 5.8 × 10^{12} vg/kg BW, the median lifespan of SD mice was 474 days (Figure 3C). Furthermore, a higher dose of AAV9/3-*modHEXB* (2.9 × 10^{13} vg/kg BW) further extended survival, with a median lifespan of 743 days. The limited efficacy of the bicistronic vector may be associated with suboptimal functional availability of HexA activity under the present experimental conditions.

i.t. administration of AAV9/3-*modHEXB* achieves widespread distribution with a favorable safety profile in non-human primates

Since most patients with TSD and SD are children and i.c.v. administration is not clinically feasible in this particular group, we investigated the biodistribution of AAV9/3-*modHEXB* using i.t. administration, which is less invasive and suitable for pediatric patients. The dose for i.t. administration in non-human primates was determined based on previously reported studies using AAV9-based vectors.[27,28] A dose of 2.0 × 10^{12} vg/kg was selected, corresponding to a total dose of approximately 4 × 10^{12} vg per animal, assuming a body weight of 2 kg. We conducted the i.t. administration of AAV9/3-*modHEXB* and AAV9/3-*GFP* at 2.0 × 10^{12} vg/kg BW each, single or combined, to the normal non-human primates cynomolgus macaques (*Macaca fascicularis*) and evaluated their biodistribution after 12 weeks of the administration and safety during the study period (Table 1). We observed no abnormalities in body weight, behavior, feeding, and fecal conditions across the 12 weeks (Figure 4A, Table S2). Statistically significant differences were found in particular hematological and biochemical tests (Table S3); however, they were within the normal range of those of previous studies,[29,30] indicating the safety of i.t. administration of AAV9/3-*modHEXB*. At necropsy, gross anatomical examination revealed no abnormalities in any of the major organs in treated animals (Table S4). To further assess safety at the tissue level, we performed comprehensive histopathological examinations of the central nervous system and major peripheral organs (Table S5). In the cerebral cortex, mild increases in glial cells were observed, accompanied by occasional eosinophilic neurons and neuronophagia. These findings are consistent with clearance processes associated with degenerating neurons and may reflect individual variability or age-related changes; a

(F) Total lipids were separated by thin-layer chromatography. Std: standard, Cbr: cerebrum, Cbl: cerebellum, Bs: brainstem.

(G) Immunohistochemical analyses of CD68 (activated glia marker) and NeuN/TUNEL (neuronal cell death marker). Scale bars: 50 μm (CD68) and 20 μm (NeuN/TUNEL). See also Table S1.

(H) Quantification of MIP-1α levels. Error bars show means ± SEM. WT, male = 3; SD, male = 1 and female = 3; AAV9/3-*modHEXB*, female = 5.

Table 1. Experimental design of safety and biodistribution studies in non-human primates

Subject No.	Age (year, month)	Sex	AAV9/3-*modHEXB* (vg/kg)	AAV9/3-*GFP* (vg/kg)
1	2 years 6 months	female	2.0×10^{12}	none
2	2 years 5 months	female	2.0×10^{12}	none
3	2 years 10 months	female	2.0×10^{12}	none
4	2 years 3 months	female	2.0×10^{12}	2.0×10^{12}
5	3 years 1 month	female	2.0×10^{12}	2.0×10^{12}

vg, vector genome.

direct causal relationship with vector administration could not be established. In the heart, focal contraction band necrosis of cardiomyocytes was detected without accompanying inflammatory or reactive changes and was, therefore, interpreted as an incidental agonal change rather than a treatment-related toxic effect. Mild lymphoid follicular hyperplasia was observed in the spleen, mandibular lymph nodes, and mucosa-associated lymphoid tissue of the stomach and large intestine. Overall, no evidence of treatment-related degenerative, inflammatory, or neoplastic changes was identified in the central nervous system or peripheral organs (Figure S5). The administration of AAV9/3-*GFP* revealed that most of the GFP-positive cells were NeuN-positive (Figure 4B), suggesting that i.t. administration of AAV9/3 vectors enables gene transfer to neurons across various brain regions. Using tissues from normal non-human primates treated with i.t. administration of AAV9/3-*modHEXB*, we assessed the tissue distribution of transgene-derived modHexB and detected β-Hex activity in diverse cerebrospinal regions, including the cerebral cortex, hippocampus, putamen, cerebellum, and brainstem, as well as in the spinal cord, the site of administration (Figure 4C). Similarly, we detected the protein expression of GFP and modHexB in several brain regions and the spinal cord (Figure 4D). To further characterize vector biodistribution in non-human primates, we quantified AAV vector genome copies in the brain and spinal cord by quantitative PCR (qPCR). AAV vector DNA was detected across multiple brain regions and the spinal cord, demonstrating widespread distribution following i.t. administration (Figure 4E). The activity of β-Hex in plasma after 12 weeks of administration was significantly increased compared to that of the pre-treatment levels (Figure 4F), while β-Hex activity in the cerebrospinal fluid increased only in one individual (Figure 4G). This limited elevation may reflect restricted secretion of modHexB from transduced neuronal cells and/or limited distribution or transport of modHexB within the central nervous system.

i.t. administration of AAV9/3-*modHEXB* shows a favorable safety profile in juvenile rats

Next, to evaluate the safety of AAV9/3-*modHEXB* in accordance with Good Laboratory Practice (GLP) standards, we administered saline (physiological saline solution, Japanese Pharmacopoeia) or AAV9/3-*modHEXB* in a single i.t. administration to male and female Crl:CD(SD) rats. To model the clinical application of AAV9/3-*modHEXB* in pediatric patients, we used 4-week-old rats, which is the minimum age for cannula insertion into the medullary cavity. The anticipated maximum clinical dose of 3.3×10^{11} vg/g brain was set as the low dose. The high dose of 9.9×10^{11} vg/g brain corresponds to 3-fold of the anticipated maximum clinical dose. On the assumption that the brain weight is 1.6 g in 4-week-old rats, doses of 5.3×10^{11} and 1.6×10^{12} vg/rat were administered for the low- and high-dose groups, respectively. Based on body weights at dosing (approximately 120 g for females and 130 g for males), these doses correspond to approximately 4.1–4.4 $\times 10^{12}$ vg/kg for the low-dose group and approximately 1.2–1.3 $\times 10^{13}$ vg/kg for the high-dose group in females and males, respectively. We performed general toxicity and biodistribution studies 26 weeks after administration. No clinical signs related to the test substance—including the effects on the central nervous, cardiovascular, and respiratory systems on the day of administration—were found in all groups (Table S6). One female in the control group died on day 1 and one male in the high-dose group died on day 11, presumably due to suppurative inflammation by misinsertion of the catheter. Erosion in the neck, wound, and soiled fur in the perineal region were observed in one rat each; however, these findings were not considered related to the test substance because they were seen only in one animal in each dose group. No abnormal body weight gains, food consumption, organ weights, or ophthalmologic, hematologic, blood chemistry, or urinalysis abnormalities related to the test substance were found in all groups (Figures 5A and 5B, Tables S7–S11). There were statistically significant differences in specific parameters; however, these were not considered related to the test substance because they were sporadic and not dose-related observations. The gross pathological changes were not considered to be test substance related because they were found sporadically in one male or female and included ventricular enlargement in the brain, dilatation with mottled grayish-white discoloration scattered in all lobules in the lung, loss of all fingers in the left hindlimb, large size of the spleen, and crust in the cervical skin (Table S12). In the AAV9/3-*modHEXB* groups, eosin-positive granules were found in a dose-related manner in neuronal cells in the central nervous system and other organs such as the heart, thoracic aorta, submandibular gland, and gastrointestinal tract (Figure 5C; Table S13). Similar intracellular features were also noted in non-human primates (Figure 4B), indicating that this finding is not restricted to a single species. To further characterize the nature of these eosin-positive granules, we performed immunohistochemical analyses in rat tissues using an anti-HexB antibody. The granules were positive to modHexB (Figures 5C and S6), suggesting that lysosomes overexpressing modHexB were stained with eosin dye. In addition, no associated inflammatory cell infiltration or degenerative changes were observed in these regions. All AAV9/3-*modHEXB*-treated males and females were positive for anti-AAV9/3 antibodies 26 weeks after administration (Table S14). The vector genome was detected in organs at weeks 13 and 26 after administration in a dose-related manner, whereas it was not detected in body fluids, excreta, and secreta (Figure 5D). The expression levels of *modHEXB* mRNA were detected in a dose-related manner at week 13 in the administration sites: cervical, thoracic, and lumbar cord; cerebrum; and heart; high expression values in these tissues were maintained until week 26 (Figure 5E). In conclusion, the

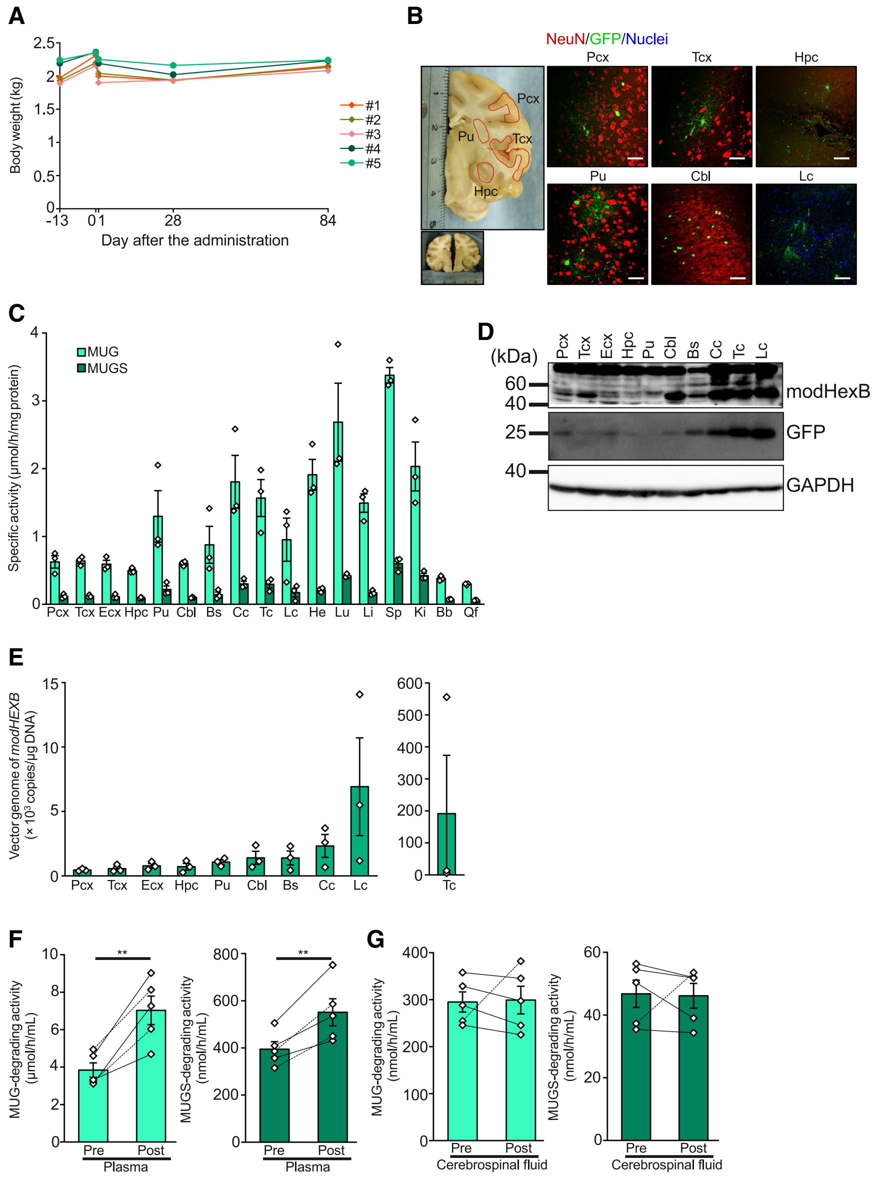

Figure 4. Toxicity and biodistribution studies of i.t. administration of AAV9/3-*modHEXB* in non-human primates

(A) Body weight change.

(B) GFP expression in brain neurons. Scale bars, 50 μm.

(C) Tissue distribution of β-Hex activity in #1, #2, and #3. Error bars show means ± SEM ($n = 3$).

(D) Expression of modHexB and GFP proteins in the brain and spinal cord.

(E) Distribution of vector genome of *modHEXB*. Error bars show means ± SEM ($n = 3$).

(F) β-Hex activity in plasma. Lines between bar graphs indicate changes in the same individual. Error bars show means ± SEM ($n = 5$). Paired *t* test was conducted. $^{**}p < 0.01$.

(G) β-Hex activity in the cerebrospinal fluid of macaques before (pre) or 3 months after (post) administration of AAV9/3-*modHEXB*. Lines between bar graphs indicate changes in the same individual. Error bars show means ± SEM ($n = 5$).

Pcx: parietal cortex, Tcx: temporal cortex, Ecx: entorhinal cortex, Hpc: hippocampus, Pu: putamen, Cbl: cerebellum, Bs: brainstem, Cc: cervical cord (C4–C6), Tc: thoracic cord (T6–T8), Lc: lumbar cord (L2–L4), He: heart, Lu: lung, Li: liver, Sp: spleen, Ki: kidney, Bb: biceps brachii, Qf: quadriceps femoris, Pre: pre-administration, Post: post-administration.

no observed adverse effect level was 1.6 × 10^{12} vg/rat (9.9 × 10^{11} vg/g brain) in both male and female rats subjected to i.t. administration of a single injection of AAV9/3-*modHEXB* under this study condition.

DISCUSSION

In this study, we demonstrated the potent efficacy of AAV9/3-*modHEXB* compared to that of the *HEXB-P2A-HEXA* vector in SD mice. We also provided evidence of the safety and biodistribution of AAV9/3-*modHEXB* in accordance with non-GLP and GLP using normal non-human primates and rats, respectively. These results suggest the potential for future clinical trials with AAV9/3-*modHEXB* in patients with infantile, juvenile, and adult types of TSD and SD.

Previous studies demonstrated that administration of rAAV2/1 monocistronic vectors carrying *HEXA* and *HEXB* into the cerebellum of 4- or 8-week-old SD mice extended their lifespan up to 615 and 233 days, respectively.[23] In other studies, administration of bicistronic vectors carrying *HEXA* and *HEXB* to SD mice at asymptomatic ages, such as neonates or 4–6 weeks of age, has also proved their efficacy in preventing the onset of the disease.[13,14,31] In contrast, in the present study, we demonstrated the superior therapeutic efficacy of AAV9/3-*modHEXB* administered to SD mice at the relatively late age of 8 weeks, with better phenotypic improvements in motor function and lifespan compared to those achieved with the same dose of the *HEXB-P2A-HEXA* bicistronic vector (Figure 3). Possible reasons for this are (1) the longer intracellular half-life of the β′β′ homodimer, i.e., 6.6 days for recombinant HexA compared to 10.7 days for recombinant modHexB[17]; (2) the higher cross-correction of the modHexB protein due to its stability at neutral pH and higher M6P-containing *N*-glycans compared to HexA[17]; (3) the stable homodimer interface of β- and β′-subunit, which facilitates dimerization more readily than αβ heterodimers,[32] as well as chimeric dimers formed with endogenous α- and β-subunits,[33] which are also expected to degrade GM2; and (4) the fact that the αβ heterodimer has one site each to interact with GM2A or degrade GM2, whereas the β′β′ homodimer has two sites each.

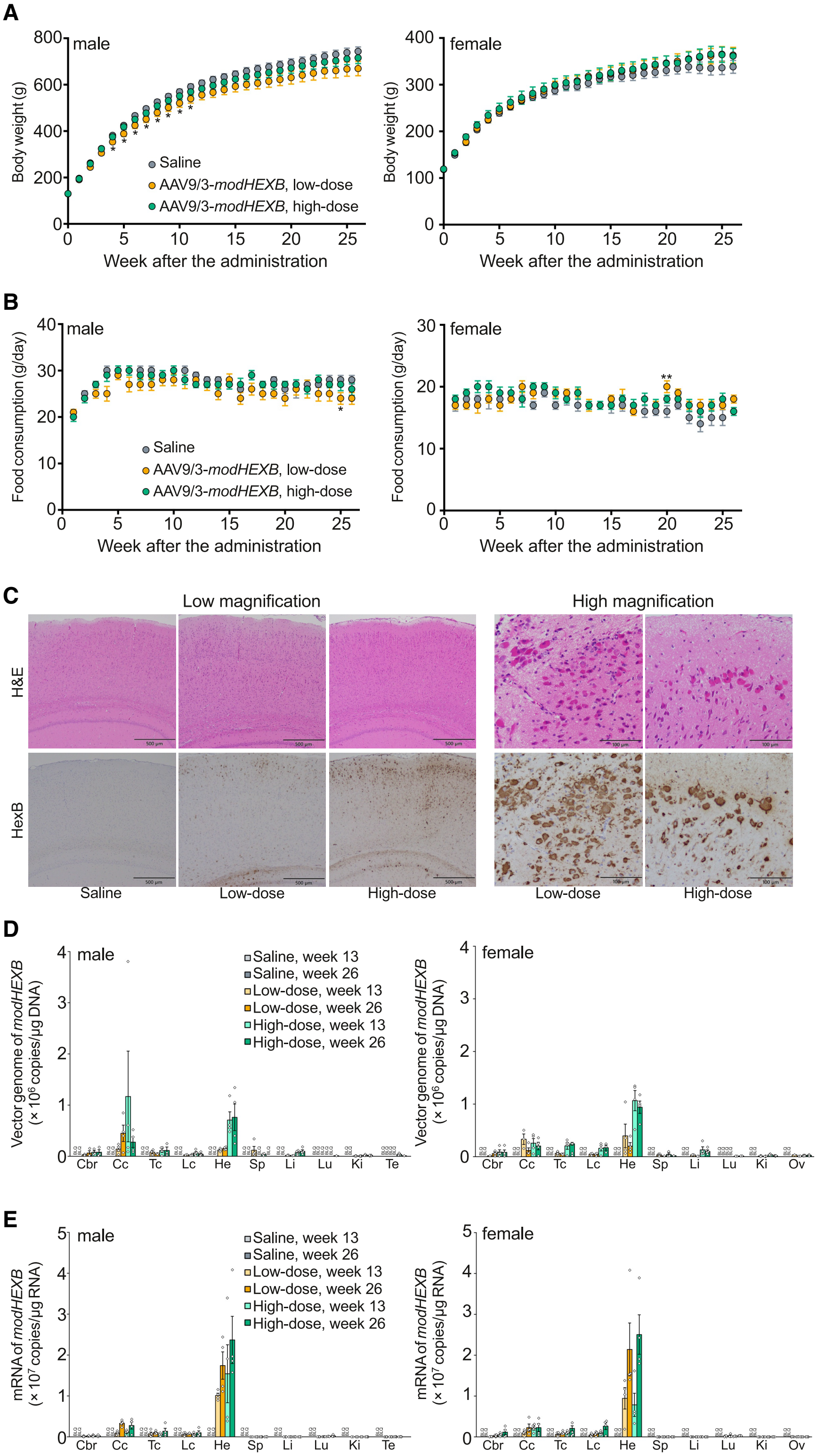

Figure 5. Toxicity and biodistribution studies of i.t. administration of AAV9/3-*modHEXB* in juvenile normal rats

Single intrathecal administration of AAV9/3-*modHEXB* at low dose (5.3×10^{11} vg/rat) or high dose (1.6×10^{12} vg/rat) to male and female rats.

(A and B) Body weight changes and food consumption. Error bars show means ± SEM (n = 9–10). One-way ANOVA with Dunnett or Steel test was conducted. *$p < 0.05$, **$p < 0.01$.

(C) Eosin-staining- and anti-HexB antibody-positive granules. Scale bars: 500 μm (low magnification) and 100 μm (high magnification).

(D and E) Distribution of vector genome and mRNA of *modHEXB*. Error bars show means ± SEM (n = 4). Samples below the lower limit of quantitation are not shown in the graph with markers. Cbr: cerebrum, Cc: cervical cord, Tc: thoracic cord, Lc: lumbar cord, He: heart, Sp: spleen, Li: liver, Lu: lung, Ki: kidney, Te: testis, Ov: ovary.

The AAV9 vector is a major tool in the development of *in vivo* gene therapy for diseases with neurological symptoms as it allows intravenous administration of vectors that cross the blood-brain barrier and introduce exogenous genes into neuronal cells. However, most intravenously administered AAV9 vectors get trapped in peripheral tissues, requiring higher doses that also increase the risk of hepatotoxicity.[34] i.t. administration of AAV vectors is expected to overcome these problems and provide efficacy and safety advantages over intravenous administration. The recent approval of *in vivo* gene therapy using i.t. administration of AAV9 vectors[35] has led us to hypothesize that i.t. administration of AAV9/3-*modHEXB* for TSD and SD is promising for clinical applications. In this study, we demonstrated that modHexB was distributed across several cerebrospinal regions following i.c.v. or i.t. administration in SD mice, non-human primates, and juvenile rats (Figures 2, 4, and 5). The modHexB expressed in neuronal cells could have reduced the accumulated substrate in the surrounding cells via CI-M6PR-mediated cross-correction. Alternatively, a recent study reported that β-Hex mediates a partial cross-correction via extracellular vesicles,[36] suggesting a mechanism of extracellular vesicle-mediated recovery of β-Hex activity in gene therapy. In peripheral tissues, modHexB expression was particularly high in the heart of SD mice and normal rats, whereas it was evenly distributed in each tissue of non-human primates. It was reported that intravenous administration of AAV9 was efficiently transduced into cardiomyocytes in mice and rats compared to other serotypes,[37] suggesting that i.c.v. or i.t. administration of AAV9/3-*modHEXB* also results in cardiac transduction via systemic exposure. In non-human primates, mild elevations in serum alkaline phosphatase and other liver-associated enzymes were observed following i.t. administration of AAV9/3-*modHEXB*. Such changes are consistent with the known biodistribution of AAV9 vectors, which can enter the systemic circulation and transduce hepatocytes even after cerebrospinal administration. In future clinical applications, transient prophylactic corticosteroid administration, as employed in approved AAV9-based therapies such as onasemnogene abeparvovec,[38,39] may be considered to mitigate liver enzyme elevations. Importantly, despite the relatively high modHexB expression in the heart and liver, no treatment-related toxicity or histopathological abnormalities were observed, supporting the peripheral safety of this vector design.

In previous studies, direct thalamic delivery of AAVrh8-*HEXA*/*HEXB* to non-human primates caused adverse events, including those with neurological signs.[40] This may be due to endoplasmic reticulum stress-induced neuronal cell death induced by the overexpression of β-Hex isozymes. Since HexS (αα) and HexA (αβ) are more sensitive to denaturation than HexB (ββ), the tolerance for the overexpression of β-Hex isozymes may be lower than that for other lysosomal enzymes. Although HexM was highly stable[32] and demonstrated therapeutic efficacy in SD mice, high dosages caused lethal adverse events that may be related to the HexM protein, with an LD_{50} of approximately 1×10^{13} vg/mouse.[41] In the present study, *modHEXB* demonstrated no adverse effects in non-human primates and rats, even at doses that had therapeutic effects on SD mice. These results suggest that the therapeutic index of *modHEXB* is superior to that of *HEXA*/*B* and *HEXM*. On the contrary, we detected eosin-positive granules in neurons of the central nervous system and peripheral tissues following AAV9/3-*modHEXB* administration to rats (Figure 5), suspecting eosinophilic infiltration due to inflammation; however, these granules were positive for anti-HexB antibody. Considering that the precursor and mature modified β-subunits have isoelectric points between 5.7 and 6.4, respectively, and those are positively charged molecules in acidic eosin solution, the negatively charged eosin dye could prefer to stain lysosomes, endosomes, and extracellular vesicles containing large amounts of the positively charged modHexB protein. Consistent with the hypothesis that overexpressed modHexB in neurons should be secreted extracellularly and exhibit cross-correction, degenerative (toxic) and adaptive (inflammatory) changes were not observed in neuronal cells in the central nervous system and other organs (Table S13). Thus, we conclude that the eosin-positive granules are not a toxicologically significant finding related to the test substance.

One of the problems with ERT is that the administered therapeutic enzymes are recognized as non-autologous proteins, causing immune reactions that include allergy and neutralizing antibody production.[42,43] As the expression of the α- and β-subunits of β-Hex is severely reduced in patients with TSD and SD, respectively, *in vivo* gene therapy with both *HEXA* and *HEXB* may trigger immune responses, resulting in the production of anti-α- and anti-β-subunit antibodies, respectively. In contrast, we substituted only 9 amino acid residues of 556 constituting the β-subunit; thus, modHexB is expected to be recognized as an autologous protein and immunologically tolerated in patients with TSD having normal HexB. Consistent with this hypothesis, i.t. administration of AAV9/3-*modHEXB* to non-human primates was not associated with anatomical and histopathological findings indicative of overt immune- or inflammation-related toxicity as those caused by non-autologous proteins, suggesting that the 94.1% amino acid homology between the human-modified and macaque β-subunits resulted in immune tolerance. Recently, it was reported that repeated administration of modified α-*N*-acetylgalactosaminidase to human *NAGA*-transgenic/*Gla*-knockout mice induced no anti-drug antibodies and is a potential ERT drug for patients with Fabry disease.[44] Although modHexB, designed based on a similar concept, is expected to have low anti-drug antibody inducibility, which was designed based on a similar concept, future studies are needed to establish an enzyme-linked immunosorbent assay (ELISA) system that complies with FDA guidelines[45] to evaluate antibody production to modHexB. On the other hand, however, this immunological assumption may not directly apply to patients with SD, who lack endogenous *HEXB* expression. In such patients, modHexB may be perceived as a non-self-antigen, potentially resulting in differential immune responses, therapeutic durability, or safety profiles compared to those in patients with TSD. Thus, the immunogenicity and long-term tolerability of modHexB may differ between these two disease populations and should be carefully evaluated in future studies.

From a translational perspective, vector dose and route of administration are critical determinants of both efficacy and safety in AAV9-based gene therapy. Intravenous administration of AAV9 at doses exceeding approximately 5×10^{13} vg/kg has

been associated with serious liver failure.[27] In contrast, i.t. administration has enabled widespread transduction of the brain and spinal cord at substantially lower total vector doses compared to intravenous delivery. In clinical and translational studies, i.t. AAV9 dosing is usually performed in a body-weight-independent manner, typically ranging from approximately 1×10^{14} to 3×10^{14} vg per subject.[28,46] Consistent with these considerations, a similar i.t. dosing range may be appropriate for future clinical trials of AAV9/3-*modHEXB* in patients with TSD and SD. Importantly, even when the modHexB protein itself is designed to be minimally immunogenic for patients with TSD, immune responses against the AAV capsid remain a major consideration in both patients with TSD and SD. Therefore, prophylactic immunosuppressive regimens are commonly incorporated into clinical AAV gene therapy protocols. For example, recent clinical studies have administered oral prednisolone at approximately 1 mg/kg starting 1 day prior to vector administration and continued for several weeks to mitigate immune-mediated adverse effects.[38] Accordingly, future clinical studies of AAV9/3-*modHEXB* are also expected to incorporate similar prophylactic immunosuppressive strategies to manage vector-associated immune responses and to ensure sustained therapeutic benefit.

Systems for early diagnosis and treatment of patients with infantile TSD and SD are essential. Several studies have reported methods for newborn screening[47–49] and potential biomarkers, including magnetic resonance imaging (MRI) changes,[50] quantitative oculomotor measures,[51] GM2 measurements in the plasma or cerebrospinal fluid,[52–54] lyso-GM2 measurements,[55] inflammatory markers,[56] and chitotriosidase level analysis in the spinal fluid involved in macrophage activation.[57] In this study, we observed that AAV9/3-*modHEXB* administration reduced GM2 and MIP-1α levels in diverse tissues. Considering that AAV9/3-*modHEXB* restores β-Hex activity in the central nervous system and peripheral tissues, studies to identify markers that reflect GM2 reduction in the brain of patients, rather than in peripheral tissues, are urgently needed.

Limitations of the study

A limitation of the present study is that the comparison between the *modHEXB* vector and the bicistronic *HEXB-P2A-HEXA* vector was performed under a single dosing condition and at a relatively late disease stage in SD mice. Differences in age at treatment initiation and the absence of immunosuppressive protocols, which were employed in other studies, may have influenced the observed therapeutic efficacy of the bicistronic vector. In addition, although the bicistronic vector encodes both *HEXA* and *HEXB*, we did not directly assess the distribution, enzymatic activity, or isoenzyme composition of HexA in the central or peripheral nervous systems. Moreover, a comprehensive side-by-side pathological evaluation—such as quantitative analyses of GM2 storage, neuroinflammation, microglial activation, and neuronal cell loss—was not performed between the two vector designs. Also, the two vectors differed in their regulatory architectures. The AAV9/3-*modHEXB* vector was designed to enhance transgene expression via the inclusion of the hGH first intron and the WPRE. In contrast, due to the packaging size limitation of AAV vectors, the AAV9/3-*HEXB-P2A-HEXA* vector consisted only of the CMV promoter, the bicistronic cassette, and a polyA signal. This difference in vector design may have contributed to the enzyme expression levels under the conditions tested. Therefore, the superior efficacy observed with AAV9/3-*modHEXB* in this study should be interpreted in the context of these experimental conditions, and further studies incorporating dose-ranging designs, earlier intervention, immunomodulatory regimens, and comprehensive analyses of Hex isoenzyme profiles will be required to comprehensively compare vector designs across different treatment paradigms.

In the present study, the non-human primate experiments were primarily designed to evaluate the *in vivo* biodistribution and translational feasibility of AAV9/3-*modHEXB* following i.t. administration. Accordingly, these experiments were conducted in normal animals rather than in a disease model, precluding direct assessment of therapeutic efficacy. In addition, vehicle-treated control groups were not included, and post-treatment findings were compared with pre-treatment baselines within the same animals. Furthermore, direct comparison between the *modHEXB* vector and the bicistronic *HEXB-P2A-HEXA* vector, as well as a formal dose-response analysis, were not performed. These design choices were made to minimize animal use in accordance with ethical considerations. Also, quantitative vector copy-number analysis was primarily focused on central nervous system tissues, and the lack of data for peripheral tissues precludes a complete assessment of systemic biodistribution and potential off-target effects. Notably, a previous study using the same AAV9/3 backbone administered via intracisterna magna delivery in pigs demonstrated vector genome distribution in both central and peripheral tissues following cerebrospinal fluid administration.[58] Although the transgene, species, and route differ from the present study, both approaches involve delivery into the cerebrospinal fluid compartment and, therefore, provide supportive contextual information regarding expected systemic exposure after cerebrospinal fluid-based administration. However, future preclinical studies will be required to evaluate central and peripheral biodistribution to fully assess cross-correction, systemic exposure, and safety, as well as long-term and dose-escalation studies to investigate risks associated with neurodegeneration, inflammatory infiltrates, or cardiomyocyte damage due to transgene overexpression.

Also, while the present study demonstrates widespread distribution of modHexB and recovery of β-Hex activity in the central nervous system, the precise cellular and molecular mechanisms underlying the functional restoration in regions with limited vector transduction remain to be fully elucidated. In particular, whether secreted modHexB contributes to enzyme activity in neighboring cells through classical CI-M6PR-dependent uptake, extracellular vesicle-mediated transfer, or other mannose-6-phosphate-independent pathways[59] warrants further investigation. Direct experimental validation of these mechanisms, including functional uptake assays using conditioned media and cell-type-specific analyses in relevant disease models, will be important to better define the contribution of intercellular enzyme transfer to therapeutic efficacy.

Finally, a limitation inherent to studies using SD mice is that the efficacy of *in vivo* gene therapy, including those using *HEXA*/*HEXB* and *modHEXB*, may be overestimated due to the

CellPress
OPEN ACCESS

metabolic pathway of GM2 by HexB and Neu3, which is not present in humans.[60] Further studies using *Hexa*$^{-/-}$ *Neu3*$^{-/-}$ mice,[61] SD cats,[62] TSD Jacob sheep,[63] and TSD wild boars[64] would be informative and further provide the basis for the use of AAV9/3-*modHEXB* in patients with TSD and SD.

RESOURCE AVAILABILITY

Lead contact

Further information and requests for resources should be directed to and will be fulfilled by the lead contact, Kohji Itoh (kitoh@tokushima-u.ac.jp).

Materials availability

Unique reagents generated in this study will be made available upon reasonable request and may require completion of a materials transfer agreement. More information and request should be directed to the lead contact.

Data and code availability

- All data reported in this paper will be shared by the lead contact upon request.
- This paper does not report any original code.
- Any additional information required to reanalyze the data reported in this paper is available from the lead contact upon request.

ACKNOWLEDGMENTS

The authors would like to thank Masako Kawase, Tsuyoshi Teshima, and Katsuhito Asai (ONODERA GT Pharma, Inc.) for preparing AAV vectors; Hideki Watanabe (ONODERA GT Pharma, Inc.) for immunohistochemistry using anti-HexB antibody; Hiromi Ogawa, Yoshiko Munesue, Chieko Ohno, Iori Itagaki (The Corporation for Production and Research of Laboratory Primates), and Drs. Kentaro Ogami and Naohide Ageyama (Tsukuba Primate Research Center) for supporting experiments with non-human primates; Dr. Norio Sakai (Osaka University) for clinical trial plan formulation; Dr. Hironobu Tan (Okayama University Hospital) for preclinical and clinical trial project management; Dr. Naozumi Ishimaru (Tokushima University) for preparing tissue sections; Miyuki Watanabe (Division of Pediatrics, Jichi Medical University) for clinical research support; and Mayuko Oe-Ike (Tokushima University) for secretarial assistance. The authors thank Editage (www.editage.com) for English language editing. This work was supported by Japan Agency for Medical Research and Development (AMED) grant numbers 17im0210605h0002, 17lm0203037h0001, 19lm0203094h0001, and 21im0210116h0003 to K.I.

AUTHOR CONTRIBUTIONS

Conceptualization, K.I.; investigation, Y.O., D.T., R.W., N.K., Y.K., H.S., S.Y., M.I., and N.T.; visualization, K.K.; data curation, K.K.; funding acquisition, K.I.; project administration, K.I.; supervision, K.I.; writing – original draft: K.K.; writing – review & editing, Y.O., Y.K., S.-i.M., and K.I.

DECLARATION OF INTERESTS

The authors declare the following competing interests: a patent "Novel adeno-associated virus virion for treatment of Tay-Sachs disease and Sandhoff disease" with patent no. PCT/JP2019/002428 (co-inventors: K.I., D.T., and S.M.) is relevant to this study. S.Y. and S.M. are employees of ONODERA GT Pharma, Inc.

STAR★METHODS

Detailed methods are provided in the online version of this paper and include the following:

- KEY RESOURCES TABLE
- EXPERIMENTAL MODEL AND STUDY PARTICIPANT DETAILS
  - Study design
  - Cells
  - Mice
  - Non-human primates
  - Rats
- METHOD DETAILS
  - Production of AAV vectors
  - AAV vector administration to cultured cells
  - Preparation of cell lysates
  - Enzyme assays
  - Immunoblot analysis
  - Immunofluorescence staining
  - Vector delivery in mice
  - Vector delivery in non-human primates
  - Vector delivery in rats
  - Tissue collection and processing from mice
  - Immunohistochemistry
  - ELISA
  - *In situ* staining for β-hex activity
  - Thin-layer chromatography
  - Rota-rod test and lifespan analysis
  - Pathological and histopathological examination in non-human primates
  - Hematological and blood chemistry analyses using non-human primates
  - General toxicity studies in accordance with GLP using normal rats
  - Biodistribution studies using normal rats
  - Quantification of AAV vector genome copies
- QUANTIFICATION AND STATISTICAL ANALYSIS

SUPPLEMENTAL INFORMATION

Supplemental information can be found online at https://doi.org/10.1016/j.xcrm.2026.102762.

Received: September 26, 2025
Revised: January 19, 2026
Accepted: March 23, 2026
Published: April 22, 2026

REFERENCES

1. Sandhoff, K., and Harzer, K. (2013). Gangliosides and gangliosidoses: principles of molecular and metabolic pathogenesis. J. Neurosci. *33*, 10195–10208. https://doi.org/10.1523/JNEUROSCI.0822-13.2013.
2. Leal, A.F., Benincore-Flórez, E., Solano-Galarza, D., Garzón Jaramillo, R.G., Echeverri-Peña, O.Y., Suarez, D.A., Alméciga-Díaz, C.J., and Espejo-Mojica, A.J. (2020). GM2 gangliosidoses: Clinical features, pathophysiological aspects, and current therapies. Int. J. Mol. Sci. *21*, 6213. https://doi.org/10.3390/ijms21176213.
3. Solomon, M., and Muro, S. (2017). Lysosomal enzyme replacement therapies: Historical development, clinical outcomes, and future perspectives. Adv. Drug Deliv. Rev. *118*, 109–134. https://doi.org/10.1016/j.addr.2017.05.004.
4. Hawkes, C., and Kar, S. (2003). Insulin-like growth factor-II/mannose-6-phosphate receptor: Widespread distribution in neurons of the central nervous system including those expressing cholinergic phenotype. J. Comp. Neurol. *458*, 113–127. https://doi.org/10.1002/cne.10578.
5. Sly, W.S., Vogler, C., Grubb, J.H., Levy, B., Galvin, N., Tan, Y., Nishioka, T., and Tomatsu, S. (2006). Enzyme therapy in mannose receptor-null mucopolysaccharidosis VII mice defines roles for the mannose 6-phosphate and mannose receptors. Proc. Natl. Acad. Sci. USA *103*, 15172–15177. https://doi.org/10.1073/pnas.0607053103.
6. Schulz, A., Ajayi, T., Specchio, N., de Los Reyes, E., Gissen, P., Ballon, D., Dyke, J.P., Cahan, H., Slasor, P., Jacoby, D., et al. (2018). CLN2 Study Group, Study of intraventricular cerliponase alfa for CLN2

disease. N. Engl. J. Med. *378*, 1898–1907. https://doi.org/10.1056/NEJMoa1712649.

7. Seo, J.H., Kosuga, M., Hamazaki, T., Shintaku, H., and Okuyama, T. (2023). Intracerebroventricular enzyme replacement therapy in patients with neuronopathic mucopolysaccharidosis type II: Final report of 5-year results from a Japanese open-label phase 1/2 study. Mol. Genet. Metab. *140*, 107709. https://doi.org/10.1016/j.ymgme.2023.107709.
8. Ellison, S., Parker, H., and Bigger, B. (2023). Advances in therapies for neurological lysosomal storage disorders. J. Inherit. Metab. Dis. *46*, 874–905. https://doi.org/10.1002/jimd.12615.
9. Kido, J., Sugawara, K., and Nakamura, K. (2023). Gene therapy for lysosomal storage diseases: Current clinical trial prospects. Front. Genet. *14*, 1064924. https://doi.org/10.3389/fgene.2023.1064924.
10. Daci, R., and Flotte, T.R. (2024). Delivery of adeno-associated virus vectors to the central nervous system for correction of single gene disorders. Int. J. Mol. Sci. *25*, 1050. https://doi.org/10.3390/ijms25021050.
11. Leinekugel, P., Michel, S., Conzelmann, E., and Sandhoff, K. (1992). Quantitative correlation between the residual activity of β-hexosaminidase A and arylsulfatase A and the severity of the resulting lysosomal storage disease. Hum. Genet. *88*, 513–523. https://doi.org/10.1007/BF00219337.
12. Flotte, T.R., Cataltepe, O., Puri, A., Batista, A.R., Moser, R., McKenna-Yasek, D., Douthwright, C., Gernoux, G., Blackwood, M., Mueller, C., et al. (2022). AAV gene therapy for Tay-Sachs disease. Nat. Med. *28*, 251–259. https://doi.org/10.1038/s41591-021-01664-4.
13. Woodley, E., Osmon, K.J.L., Thompson, P., Richmond, C., Chen, Z., Gray, S.J., and Walia, J.S. (2019). Efficacy of a bicistronic vector for correction of Sandhoff disease in a mouse model. Mol. Ther. Methods Clin. Dev. *12*, 47–57. https://doi.org/10.1016/j.omtm.2018.10.011.
14. Lahey, H.G., Webber, C.J., Golebiowski, D., Izzo, C.M., Horn, E., Taghian, T., Rodriguez, P., Batista, A.R., Ellis, L.E., Hwang, M., et al. (2020). Pronounced therapeutic benefit of a single bidirectional AAV vector administered systemically in Sandhoff mice. Mol. Ther. *28*, 2150–2160. https://doi.org/10.1016/j.ymthe.2020.06.021.
15. Eichler, F., Cataltepe, O.I., Daci, R., Puri, A.S., Taghian, T., Jiang, X., Shazeeb, M.S., Kuhn, A., Hader, A., Celik, H., et al. (2025). Dual-vector rAAVrh8 gene therapy for GM2 gangliosidosis: a phase 1/2 trial. Nat. Med. *31*, 2927–2935. https://doi.org/10.1038/s41591-025-03822-4.
16. Flotte, T.R., Blackwood, M., Arjomandnejad, M., Harkins, A., Sylvia, K., Iyer, S., Kokoski, D., Artinian, R., and Keeler, A.M. (2025). Characterization of immune responses to rAAVrh8 gene therapy for GM2 gangliosidosis in phase 1/2 trial. Preprint at medRxiv. https://doi.org/10.1101/2025.05.07.25327170.
17. Kitakaze, K., Mizutani, Y., Sugiyama, E., Tasaki, C., Tsuji, D., Maita, N., Hirokawa, T., Asanuma, D., Kamiya, M., Sato, K., et al. (2016). Protease-resistant modified human β-hexosaminidase B ameliorates symptoms in GM2 gangliosidosis model. J. Clin. Investig. *126*, 1691–1703. https://doi.org/10.1172/JCI85300.
18. Iida, A., Takino, N., Miyauchi, H., Shimazaki, K., and Muramatsu, S.i. (2013). Systemic delivery of tyrosine-mutant AAV vectors results in robust transduction of neurons in adult mice. BioMed Res. Int. *2013*, 974819. https://doi.org/10.1155/2013/974819.
19. Tanaka, H., Tsuji, D., Watanabe, R., Ohnishi, Y., Kitaguchi, S., Nakae, R., Teramoto, H., Tsukimoto, J., Horii, Y., and Itoh, K. (2022). Aberrant autophagy in lysosomal storage disorders marked by a lysosomal SNARE protein shortage due to suppression of endocytosis. J. Inherit. Metab. Dis. *45*, 1191–1202. https://doi.org/10.1002/jimd.12558.
20. Sango, K., Yamanaka, S., Hoffmann, A., Okuda, Y., Grinberg, A., Westphal, H., McDonald, M.P., Crawley, J.N., Sandhoff, K., Suzuki, K., et al. (1995). Mouse models of Tay-Sachs and Sandhoff diseases differ in neurologic phenotype and ganglioside metabolism. Nat. Genet. *11*, 170–176. https://doi.org/10.1038/ng1095-170.
21. Sango, K., McDonald, M.P., Crawley, J.N., Mack, M.L., Tifft, C.J., Skop, E., Starr, C.M., Hoffmann, A., Sandhoff, K., Suzuki, K., et al. (1996). Mice lacking both subunits of lysosomal β-hexosaminidase display gangliosidosis and mucopolysaccharidosis. Nat. Genet. *14*, 348–352. https://doi.org/10.1038/ng1196-348.
22. Cachón-González, M.B., Wang, S.Z., McNair, R., Bradley, J., Lunn, D., Ziegler, R., Cheng, S.H., and Cox, T.M. (2012). Gene transfer corrects acute GM2 gangliosidosis—potential therapeutic contribution of perivascular enzyme flow. Mol. Ther. *20*, 1489–1500. https://doi.org/10.1038/mt.2012.44.
23. Cachón-González, M.B., Wang, S.Z., Ziegler, R., Cheng, S.H., and Cox, T.M. (2014). Reversibility of neuropathology in Tay–Sachs-related diseases. Hum. Mol. Genet. *23*, 730–748. https://doi.org/10.1093/hmg/ddt459.
24. Kurokawa, Y., Osaka, H., Kouga, T., Jimbo, E., Muramatsu, K., Nakamura, S., Takayanagi, Y., Onaka, T., Muramatsu, S.I., and Yamagata, T. (2021). Gene therapy in a mouse model of Niemann-Pick disease type C1. Hum. Gene Ther. *32*, 589–598. https://doi.org/10.1089/hum.2020.175.
25. Tsuji, D., Kuroki, A., Ishibashi, Y., Itakura, T., Kuwahara, J., Yamanaka, S., and Itoh, K. (2005). Specific induction of macrophage inflammatory protein 1-α in glial cells of Sandhoff disease model mice associated with accumulation of *N*-acetylhexosaminyl glycoconjugates. J. Neurochem. *92*, 1497–1507. https://doi.org/10.1111/j.1471-4159.2005.02986.x.
26. Tsuji, D., Kuroki, A., Ishibashi, Y., Itakura, T., and Itoh, K. (2005). Metabolic correction in microglia derived from Sandhoff disease model mice. J. Neurochem. *94*, 1631–1638. https://doi.org/10.1111/j.1471-4159.2005.03317.x.
27. Muramatsu, S.I. (2024). AAV vectors advance cardiac gene therapy while overcoming challenges. Int. J. Cardiol. *400*, 131704. https://doi.org/10.1016/j.ijcard.2023.131704.
28. Kumagai, S., Nakajima, T., Shimazaki, K., Kakiuchi, T., Harada, N., Ohba, H., Onuki, Y., Takino, N., Ito, M., Sato, M., et al. (2023). Early distribution of ^{18}F-labeled AAV9 vectors in the cerebrospinal fluid after intracerebroventricular or intracisternal magna infusion in non-human primates. J. Gene Med. *25*, e3457. https://doi.org/10.1002/jgm.3457.
29. Koo, B.S., Lee, D.H., Kang, P., Jeong, K.J., Lee, S., Kim, K., Lee, Y., Huh, J.W., Kim, Y.H., Park, S.J., et al. (2019). Reference values of hematological and biochemical parameters in young-adult cynomolgus monkey (*Macaca fascicularis*) and rhesus monkey (*Macaca mulatta*) anesthetized with ketamine hydrochloride. Lab. Anim. Res. *35*, 7. https://doi.org/10.1186/s42826-019-0006-0.
30. Bakker, J., Maaskant, A., Wegman, M., Zijlmans, D.G.M., Hage, P., Langermans, J.A.M., and Remarque, E.J. (2023). Reference intervals and percentiles for hematologic and serum biochemical values in captive bred rhesus (*Macaca mulatta*) and cynomolgus macaques (*Macaca fascicularis*). Animals. *13*, 445. https://doi.org/10.3390/ani13030445.
31. Ryckman, A.E., Deschenes, N.M., Quinville, B.M., Osmon, K.J.L., Mitchell, M., Chen, Z., Gray, S.J., and Walia, J.S. (2024). Intrathecal delivery of a bicistronic AAV9 vector expressing β-hexosaminidase A corrects Sandhoff disease in a murine model: A dosage study. Mol. Ther. Methods Clin. Dev. *32*, 101168. https://doi.org/10.1016/j.omtm.2023.101168.
32. Tropak, M.B., Yonekawa, S., Karumuthil-Melethil, S., Thompson, P., Wakarchuk, W., Gray, S.J., Walia, J.S., Mark, B.L., and Mahuran, D. (2016). Construction of a hybrid β-hexosaminidase subunit capable of forming stable homodimers that hydrolyze GM2 ganglioside *in vivo*. Mol. Ther. Methods Clin. Dev. *3*, 15057. https://doi.org/10.1038/mtm.2015.57.
33. Sinici, I., Yonekawa, S., Tkachyova, I., Gray, S.J., Samulski, R.J., Wakarchuk, W., Mark, B.L., and Mahuran, D.J. (2013). In cellulo examination of a beta-alpha hybrid construct of beta-hexosaminidase A subunits, reported to interact with the GM2 activator protein and hydrolyze GM2 ganglioside. PLoS One *8*, e57908. https://doi.org/10.1371/journal.pone.0057908.
34. Hinderer, C., Katz, N., Buza, E.L., Dyer, C., Goode, T., Bell, P., Richman, L.K., and Wilson, J.M. (2018). Severe toxicity in nonhuman primates and piglets following high-dose intravenous administration of an

CellPress
OPEN ACCESS

adeno-associated virus vector expressing human SMN. Hum. Gene Ther. *29*, 285–298. https://doi.org/10.1089/hum.2018.015.

35. Rao, V.K., Kapp, D., and Schroth, M. (2018). Gene therapy for spinal muscular atrophy: An emerging treatment option for a devastating disease. J. Manag. Care Spec. Pharm. *24*, S3–S16. https://doi.org/10.18553/jmcp.2018.24.12-a.s3.

36. Calzoni, E., Cerrotti, G., Sagini, K., Delo, F., Buratta, S., Pellegrino, R.M., Alabed, H.B.R., Fratini, F., Emiliani, C., and Urbanelli, L. (2024). Evidence of lysosomal β-hexosaminidase enzymatic activity associated with extracellular vesicles: Potential applications for the correction of Sandhoff disease. J. Funct. Biomater. *15*, 153. https://doi.org/10.3390/jfb15060153.

37. Zincarelli, C., Soltys, S., Rengo, G., and Rabinowitz, J.E. (2008). Analysis of AAV serotypes 1-9 mediated gene expression and tropism in mice after systemic injection. Mol. Ther. *16*, 1073–1080. https://doi.org/10.1038/mt.2008.76.

38. Proud, C.M., Vũ, D.C., Wilmshurst, J.M., Sanmaneechai, O., Gulati, S., Xiong, H., Moreno, H.C., Tay, S.K.H., Thong, M.K., Born, A.P., et al. (2025). Intrathecal onasemnogene abeparvovec in treatment-naive patients with spinal muscular atrophy: a phase 3, randomized controlled trial. Nat. Med. *32*, 481–487. https://doi.org/10.1038/s41591-025-04103-w.

39. Kwon, J.M., Munell, F., Le Goff, L., Yuge, K., Kato, T., Cances, C., De Waele, L., Woodcock, I.R., Mercuri, E.M., Proud, C.M., et al. (2026). Intrathecal onasemnogene abeparvovec for treatment-experienced patients with spinal muscular atrophy: a phase 3b, open-label trial. Nat. Med. *32*, 488–493. https://doi.org/10.1038/s41591-025-04119-2.

40. Golebiowski, D., van der Bom, I.M.J., Kwon, C.S., Miller, A.D., Petrosky, K., Bradbury, A.M., Maitland, S., Kühn, A.L., Bishop, N., Curran, E., et al. (2017). Direct intracranial injection of AAVrh8 encoding monkey β-N-acetylhexosaminidase causes neurotoxicity in the primate brain. Hum. Gene Ther. *28*, 510–522. https://doi.org/10.1089/hum.2016.109.

41. Osmon, K.J., Thompson, P., Woodley, E., Karumuthil-Melethil, S., Heindel, C., Keimel, J.G., Kaemmerer, W.F., Gray, S.J., and Walia, J.S. (2022). Treatment of GM2 gangliosidosis in adult Sandhoff mice using an intravenous self-complementary hexosaminidase vector. Curr. Gene Ther. *22*, 262–276. https://doi.org/10.2174/1566523221666210916153051.

42. de Vries, J.M., Kuperus, E., Hoogeveen-Westerveld, M., Kroos, M.A., Wens, S.C.A., Stok, M., van der Beek, N.A.M.E., Kruijshaar, M.E., Rizopoulos, D., van Doorn, P.A., et al. (2017). Pompe disease in adulthood: effects of antibody formation on enzyme replacement therapy. Genet. Med. *19*, 90–97. https://doi.org/10.1038/gim.2016.70.

43. Wang, J., Lozier, J., Johnson, G., Kirshner, S., Verthelyi, D., Pariser, A., Shores, E., and Rosenberg, A. (2008). Neutralizing antibodies to therapeutic enzymes: considerations for testing, prevention and treatment. Nat. Biotechnol. *26*, 901–908. https://doi.org/10.1038/nbt.1484.

44. Sakuraba, H., Tsukimura, T., Shiga, T., Saito, S., Tajima, Y., Kawashima, I., Fukushige, T., and Togawa, T. (2025). Enzyme replacement therapy using a modified α-*N*-acetylgalactosaminidase to suppress progressive glycolipid accumulation and escape from antidrug antibody formation in young human *NAGA*-transgenic/*Gla*-knockout mice. Mol. Genet. Metab. *145*, 109151. https://doi.org/10.1016/j.ymgme.2025.109151.

45. US Food and Drug Administration (2019). Immunogenicity Testing of Therapeutic Protein Products—Developing and Validating Assays for Anti-drug Antibody Detection (Center for Biologics Evaluation and Research (CBER)). https://www.fda.gov/regulatory-information/search-fda-guidance-documents/immunogenicity-testing-therapeutic-protein-products-developing-and-validating-assays-anti-drug.

46. Okai, T., Sato, S., Yasuno, H., Nakayama, M., Yamamoto, S., Sjöqvist, S., Otake, K., Nakashima, M., Deshpande, M., Galbreath, E., et al. (2025). Biodistribution of AAV1, AAV5, AAV9, and AAVDJ serotypes after intracisterna magna delivery in non-human primates. Mol. Ther. Methods Clin. Dev. *33*, 101593. https://doi.org/10.1016/j.omtm.2025.101593.

47. Chamoles, N.A., Blanco, M., Gaggioli, D., and Casentini, C. (2002). Tay-Sachs and Sandhoff diseases: enzymatic diagnosis in dried blood spots on filter paper: retrospective diagnoses in newborn-screening cards. Clin. Chim. Acta *318*, 133–137. https://doi.org/10.1016/s0009-8981(02)00002-5.

48. Fitterer, B., Hall, P., Antonishyn, N., Desikan, R., Gelb, M., and Lehotay, D. (2014). Incidence and carrier frequency of Sandhoff disease in Saskatchewan determined using a novel substrate with detection by tandem mass spectrometry and molecular genetic analysis. Mol. Genet. Metab. *111*, 382–389. https://doi.org/10.1016/j.ymgme.2014.01.002.

49. Herbst, Z.M., Hold, S., Gelb, M.H., and Khaledi, H. (2024). Tandem mass spectrometric enzyme assay for simultaneous detection of Tay-Sachs and Sandhoff diseases in dried blood spots for newborn screening. Mol. Genet. Metab. *142*, 108517. https://doi.org/10.1016/j.ymgme.2024.108517.

50. Bradbury, A.M., Gray-Edwards, H.L., Shirley, J.L., McCurdy, V.J., Colaco, A.N., Randle, A.N., Christopherson, P.W., Bird, A.C., Johnson, A.K., Wilson, D.U., et al. (2015). Biomarkers for disease progression and AAV therapeutic efficacy in feline Sandhoff disease. Exp. Neurol. *263*, 102–112. https://doi.org/10.1016/j.expneurol.2014.09.020.

51. Stephen, C.D., Balkwill, D., James, P., Haxton, E., Sassower, K., Schmahmann, J.D., Eichler, F., and Lewis, R. (2020). Quantitative oculomotor and nonmotor assessments in late-onset GM2 gangliosidosis. Neurology *94*, e705–e717. https://doi.org/10.1212/WNL.0000000000008959.

52. Gu, J., Tifft, C.J., and Soldin, S.J. (2008). Simultaneous quantification of GM1 and GM2 gangliosides by isotope dilution tandem mass spectrometry. Clin. Biochem. *41*, 413–417. https://doi.org/10.1016/j.clinbiochem.2007.12.026.

53. Blondel, A., Kraoua, I., Marcelino, C., Khrouf, W., Schlemmer, D., Ganne, B., Caillaud, C., Fernández-Eulate, G., Turki, I.B.Y., Dauriat, B., et al. (2023). Plasma GM2 ganglioside potential biomarker for diagnosis, prognosis and disease monitoring of GM2-Gangliosidosis. Mol. Genet. Metab. *138*, 106983. https://doi.org/10.1016/j.ymgme.2022.106983.

54. Kim, J., Byeon, S.K., Oglesbee, D., Schultz, M.J., Matern, D., and Pandey, A. (2024). A multiplexed targeted method for profiling of serum gangliosides and glycosphingolipids: application to GM2-gangliosidosis. Anal. Bioanal. Chem. *416*, 5689–5699. https://doi.org/10.1007/s00216-024-05487-3.

55. Kodama, T., Togawa, T., Tsukimura, T., Kawashima, I., Matsuoka, K., Kitakaze, K., Tsuji, D., Itoh, K., Ishida, Y.I., Suzuki, M., et al. (2011). Lyso-GM2 ganglioside: a possible biomarker of Tay-Sachs disease and Sandhoff disease. PLoS One *6*, e29074. https://doi.org/10.1371/journal.pone.0029074.

56. Utz, J.R.J., Crutcher, T., Schneider, J., Sorgen, P., and Whitley, C.B. (2015). Biomarkers of central nervous system inflammation in infantile and juvenile gangliosidoses. Mol. Genet. Metab. *114*, 274–280. https://doi.org/10.1016/j.ymgme.2014.11.015.

57. Kim, S., Whitley, C.B., and Jarnes, J.R. (2021). Chitotriosidase as a biomarker for gangliosidoses. Mol. Genet. Metab. Rep. *29*, 100803. https://doi.org/10.1016/j.ymgmr.2021.100803.

58. Nakamura, S., Osaka, H., Muramatsu, S.I., Takino, N., Ito, M., Jimbo, E.F., Watanabe, C., Hishikawa, S., Nakajima, T., and Yamagata, T. (2021). Intracisterna magna delivery of an AAV vector with the GLUT1 promoter in a pig recapitulates the physiological expression of *SLC2A1*. Gene Ther. *28*, 329–338. https://doi.org/10.1038/s41434-020-00203-z.

59. Shinoda, C., Kitakaze, K., Sasai, Y., Nishioka, S.I., Kobayashi, I., Sumitani, M., Tatematsu, K.I., Iizuka, T., Harazono, A., Mitani, A., et al. (2025). *N*-glycan-modified α-L-iduronidase produced by transgenic silkworms ameliorates clinical signs in a Japanese macaque with mucopolysaccharidosis I. Commun. Med. *5*, 128. https://doi.org/10.1038/s43856-025-00841-7.

60. Yuziuk, J.A., Bertoni, C., Beccari, T., Orlacchio, A., Wu, Y.Y., Li, S.C., and Li, Y.T. (1998). Specificity of mouse G_{M2} activator protein and β-*N*-acetylhexosaminidases A and B. Similarities and differences with their human counterparts in the catabolism of G_{M2}. J. Biol. Chem. *273*, 66–72. https://doi.org/10.1074/jbc.273.1.66.

61. Seyrantepe, V., Demir, S.A., Timur, Z.K., Von Gerichten, J., Marsching, C., Erdemli, E., Oztas, E., Takahashi, K., Yamaguchi, K., Ates, N., et al. (2018).

Murine Sialidase Neu3 facilitates GM2 degradation and bypass in mouse model of Tay-Sachs disease. Exp. Neurol. *299*, 26–41. https://doi.org/10.1016/j.expneurol.2017.09.012.

62. Lawson, C.A., and Martin, D.R. (2016). Animal models of GM2 gangliosidosis: utility and limitations. Appl. Clin. Genet. *9*, 111–120. https://doi.org/10.2147/TACG.S85354.
63. Story, B., Taghian, T., Gallagher, J., Koehler, J., Taylor, A., Randle, A., Nielsen, K., Gross, A., Maguire, A., Carl, S., et al. (2021). Natural history of Tay-Sachs disease in sheep. Mol. Genet. Metab. *134*, 164–174. https://doi.org/10.1016/j.ymgme.2021.08.009.
64. Bertani, V., Prioni, S., Di Lecce, R., Gazza, F., Ragionieri, L., Merialdi, G., Bonilauri, P., Jagannathan, V., Grassi, S., Cabitta, L., et al. (2021). A pathogenic *HEXA* missense variant in wild boars with Tay-Sachs disease. Mol. Genet. Metab. *133*, 297–306. https://doi.org/10.1016/j.ymgme.2021.05.001.
65. Kotani, M., Ozawa, H., Kawashima, I., Ando, S., and Tai, T. (1992). Generation of one set of monoclonal antibodies specific for a-pathway ganglio-series gangliosides. Biochim. Biophys. Acta *1117*, 97–103. https://doi.org/10.1016/0304-4165(92)90168-t.
66. Izumi, T., Fukuyama, Y., Tsuji, A., Yamanaka, T., Hirabayashi, Y., and Suzuki, Y. (1988). GM2-gangliosidosis: BI variant with thermostable β-hexosaminidase A and molecular analysis of the mutant enzyme. In Lipid Storage Disorders. NATO ASI Series, R. Salvayre, L. Douste-Blazy, and S. Gatt, eds. (Boston, MA: Springer), pp. 237–245. https://doi.org/10.1007/978-1-4613-1029-7_27.
67. Utsumi, K., Tsuji, A., Kase, R., Tanaka, A., Tanaka, T., Uyama, E., Ozawa, T., Sakuraba, H., Komaba, Y., Kawabe, M., et al. (2002). Western blotting analysis of the β-hexosaminidase α- and β-subunits in cultured fibroblasts from cases of various forms of GM2 gangliosidosis. Acta Neurol. Scand. *105*, 427–430. https://doi.org/10.1034/j.1600-0404.2002.01097.x.
68. Cheung, Y.T., Lau, W.K.W., Yu, M.S., Lai, C.S.W., Yeung, S.C., So, K.F., and Chang, R.C.C. (2009). Effects of all-trans-retinoic acid on human SH-SY5Y neuroblastoma as *in vitro* model in neurotoxicity research. Neurotoxicology *30*, 127–135. https://doi.org/10.1016/j.neuro.2008.11.001.
69. Suzuki, K. (1987). Enzymatic diagnosis of sphingolipidoses. Methods Enzymol. *138*, 727–762. https://doi.org/10.1016/0076-6879(87)38063-2.
70. Hayashi, M. (1965). Histochemical demonstration of N-acetyl-β-glucosaminidase employing naphthol AS-BI N-acetyl-β-glucosaminide as substrate. J. Histochem. Cytochem. *13*, 355–360. https://doi.org/10.1177/13.5.355.

CellPress
OPEN ACCESS

STAR★METHODS

KEY RESOURCES TABLE

REAGENT or RESOURCE	SOURCE	IDENTIFIER
**Antibodies**		
GM2-specific mouse mAb (GMB28: IgM)	Provided by Dr. Tadashi Tai	Kotani et al.[65]
Anti-NAG(A), antiserum against human HexA	Provided by Dr. Akihiko Tsuji	Izumi et al.,[66] Utsumi et al.[67]
Anti-GFP antibody (JL-8)	Takara Bio	Cat# 632381; RRID: AB_2313808
anti-NeuN antibody	Merck Millipore	Cat# ABN78; RRID: AB_10807945
anti-GAPDH antibody (6C5)	Santa Cruz Biotechnology	Cat# ab8245; RRID: AB_2107448
anti-HexB antibody	Abcam	Cat# ab140649; RRID: AB_3065101
Anti-CD68 antibody (FA-11)	Thermo Fisher Scientific	Cat# 14-0681-82; RRID: AB_2572857
Anti-rabbit IgG, HRP-linked Antibody	Cell Signaling Technology	Cat# 7074; RRID: AB_2099233
Anti-mouse IgG, HRP-linked Antibody	Cell Signaling Technology	Cat# 7076; RRID: AB_330924
Goat Anti-Mouse IgG+IgM H&L (FITC) preadsorbed	Abcam	Cat# ab47830; RRID: AB_955209
**Chemicals, peptides, and recombinant proteins**		
4-methylumbelliferyl-*N*-acetyl-β-D-glucosaminide	Sigma-Aldrich	Cat# 474502
4-methylumbelliferyl-6-sulfo-*N*-acetyl-β-D-glucosaminide	Merck	Cat# 454428
Blocking One	Nacalai Tesque	Cat# 03953-95
Western Lightning Plus-ECL	Perkin Elmer	Cat# NEL104001EA
Western Lightning Ultra	Perkin Elmer	Cat# NEL111001EA
Hoechst33258	Sigma-Aldrich	Cat# 94403
Tissue-Tek O.C.T. compound	Sakura Finetek Japan	Cat# 4583
HistoVT One	Nacalai Tesque	Cat# 06380-76
antigen retrieval buffer	Abcam	Cat# ab93684
AS-BI *N*-acetyl-β-D-glucosaminide	Merck	Cat# N4006
**Critical commercial assays**		
DC Protein Assay	Bio-Rad	Cat# 5000112
DeadEnd Colorimetric TUNEL system	Promega	Cat# G7360
VECTASTAIN Elite ABC Kit	Vector Laboratories	Cat# PK-6100
Tissue-Tek Mayer Hematoxylin For Prisma standard solution reservoir	Sakura Finetek Japan	Cat# 6186-4P
Mouse CCL3/MIP-1 alpha ELISA Kit - Quantikine	R&D Systems	Cat# MMA00
QIAamp DNA Blood Mini Kit	QIAGEN	Cat# 51106
QIAamp 96 DNA QIAcube HT Kit	QIAGEN	Cat# 51331
QIAamp Fast DNA Stool Mini Kit	QIAGEN	Cat# 51604
TaqPath qPCR Master Mix	Thermo Fisher Scientific	Cat# A15297
**Experimental models: Cell lines**		
SH-SY5Y	ATCC	Cat# 94030304 RRID: CVCL_0019
iPSCs derived from patients with TSD	Tanaka et al. (*19*)	N/A

(*Continued on next page*)

Continued		
REAGENT or RESOURCE	SOURCE	IDENTIFIER
Experimental models: Organisms/strains		
SD model mice	Provided by Dr. R.L. Proia	Sango et al.[20]
cynomolgus macaque	Tsukuba Primate Research Center	N/A
Crl:CD(SD) rat	Jackson Laboratory Japan	RRID: RGD_734476
Recombinant DNA		
AAV9/3-*modHEXB*	This study	N/A
AAV9/3-*HEXB-P2A-HEXA*	This study	N/A
AAV9/3-*GFP*	This study	N/A
Software and algorithms		
SAS System ver. 9.4	SAS Institute	RRID: SCR_008567
Prism ver. 8.42	GraphPad Software	RRID: SCR_002798
ImageJ ver. 1.52	NIH	RRID: SCR_003070

EXPERIMENTAL MODEL AND STUDY PARTICIPANT DETAILS

Study design

The goal of this study was to assess the efficacy in SD mice and safety in non-human primates and rats of the *i.c.v./i.t.* administration of the AAV9/3 vector encoding *modHEXB*. The sample size for each experiment is included in the figure legends. The number of animals used was selected based on previous analyses conducted in the same model. Sample collection, treatment, and processing information are included in Results and Methods sections.

Cells

Human neuroblastoma cell line SH-SY5Y (94030304, ATCC, Manassas, VA) was maintained in DMEM/F12 supplemented with 10% (v/v) FBS (Biosera, Cholet, France), 100 μg/mL Streptomycin (Sigma-Aldrich, St. Louis, MO), and 70 μg/mL Penicillin G (Sigma-Aldrich). A control human induced pluripotent stem cell (iPSC) line (201B7) was obtained from the RIKEN BRC Cell Bank (Tsukuba, Japan). TSD iPSC lines were previously established and characterized.[19] iPSCs were maintained on SNL feeder cells using Repro Stem medium and Primate ES Cell Medium (ReproCELL, Yokohama, Japan) supplemented with 5 ng/mL basic fibroblast growth facto. All cell lines were cultured at 37°C in a humidified atmosphere of 5% CO_2 and 95% air. Cells were obtained from authenticated repositories or previously characterized sources. Cell identity was confirmed by morphology during routine culture. The cell lines used in this study were not tested for mycoplasma contamination.

Mice

Experiments using mice were approved by the ethics committee on animal care at Tokushima University and were performed in accordance with institutional guidelines for animal care at Tokushima University (approval No. T28-70). SD model mice (*Hexb*$^{-/-}$, C57BL/6 × 129Sv background) were provided by Dr. R.L. Proia (National Institute of Diabetes and Digestive and Kidney Diseases, NIH). The mice were bred by mating them with C57BL/6 mice (Japan SLC, Hamamatsu, Japan) and maintained under specific pathogen-free conditions in the animal facilities at Tokushima University. Mice were genotyped and randomly assigned to the experimental groups. Animals were used at the ages specified in each experiment. Based on previous reports indicating no sex differences in central nervous system phenotypes or lifespan in this mouse model, sex was not considered as a biological variable.

Non-human primates

Experiments using non-human primates were approved by the Use and Care of Experimental Animals Committee of Jichi Medical University (approval No. 17214) and National Institutes of Biomedical Innovation, Health and Nutrition (NIBIOHN, approval No. DS29-43), and were performed in accordance with the Rules for Animal Care and Management of the Tsukuba Primate Research Center (TPRC), the Guiding Principles for Animal Experiments Using Non-human Primates formulated by the Primate Society of Japan (Primate Society of Japan, 1986), and the Guide for the Care and Use of Laboratory Animals (Institute for Laboratory Animal Research, 2011). The female cynomolgus macaques (*Macaca fascicularis*) of 2–3 years used in this study were bred and maintained at TPRC at the NIBIOHN. The macaques were housed in stainless-steel cages (W500 × D860 × H800 mm) that could be coupled together by opening a panel of the side wall. Animal rooms were maintained at a temperature of 25 ± 3°C and 50–70% humidity, with 12 air changes per hour and a 12/12-h light/dark cycle. The animals could visually, aurally, and olfactory sense their roommates at the front and both sides of the cages. Each macaque was fed 70 g of commercial feed (CMK-2; CLEA Japan, Tokyo, Japan) and 100 g of apples daily. Tap water was supplied *ad libitum*. The health of all animals, such as appetite, fur-coat appearance, excreta, menstrual blood, and clinical and behavioral statuses, was monitored by veterinarians and experienced animal technicians twice a

day (morning and afternoon). Only female macaques were used in this study; therefore, potential sex-dependent differences could not be evaluated.

Rats

Studies using rats were approved by the Institutional Animal Care and Use Committee (IACUC) of CMIC Pharma Science Co., Ltd. (approval No. IACUC-CBR-2204-011). Crl:CD(SD) rats (3 weeks of age) were provided by the Jackson Laboratory Japan. After quarantine and acclimation, the rats were housed individually in plastic cages with wire lids and bedding materials (W20 × L30 × H18 cm), managed in a specific pathogen-free condition under a 12-h shift of the light-dark cycle, and fed with pellet food CRF-1 (Oriental Yeast, Tokyo, Japan) sterilized by γ-ray and filtered tap water (Hokuto, Japan) *ad libitum*. Temperature and relative humidity were kept at 20.3°C–23.2°C and 43.4–66.4%, respectively, during the study period. The animals were distributed into 3 groups of 10 males and 10 females in the toxicity study unit, and 6 groups of 4 males and 4 females in the biodistribution study unit, with similar mean body weight based on body weights on the day of grouping. The facility has earned full accreditation from the AAALAC International (File No. 001182). This study, except measurement of antibody and genetic tests, was conducted in compliance with "Ministerial Ordinance on Good Laboratory Practice for Nonclinical Safety Studies of Regenerative Medical Products" (Ordinance of the Ministry of Health, Labor and Welfare of Japan No. 88 of July 30, 2014). Measurement of AAV9/3-*modHEXB* genome titer and *modHEXB* mRNA expression levels were performed in conformity with the Regulation for Enforcement of the Act on Securing Quality, Efficacy and Safety of Products Including Pharmaceuticals and Medical Devices (Order of the Ministry of Health and Welfare of Japan No. 1 of February 1, 1961). This study was performed in conformity with the guidelines; "Guidelines for Toxicity Studies of Drugs" (Notification No. 24 of First Evaluation and Registration Division, September 11, 1989. Notification No. 655 of the Evaluation and Licensing Division, April 5, 1999, Ministry of Health, Labor and Welfare of Japan) and "Guideline on Ensuring the Quality and Safety of Gene Therapy Products" (PSEHB/MDED Notification No.0709-2, July 9, 2019). Both male and female rats were included to reduce potential sex bias; however, the study was not designed or powered to evaluate sex-specific differences.

METHOD DETAILS

Production of AAV vectors

The AAV vector plasmid contained an expression cassette consisting of the cytomegalovirus immediate-early promoter, human growth hormone 1st intron, cDNA of *GFP* or *modHEXB*, woodchuck hepatitis virus posttranscriptional regulatory element, and the simian virus 40 polyadenylation signal sequence between the inverted terminal repeats of the AAV3 genome. AAV9 vp cDNA was synthesized, and the sequence was identical to that previously described,[18] except for the substitution of thymidine for adenine 1337, which introduces an amino acid change from tyrosine to phenylalanine at position 446. Recombinant AAV vectors were produced by transient transfection of HEK293 cells using the vector plasmid, an AAV3 rep and the tyrosine-mutant AAV9 vp expression plasmids, and the adenoviral helper plasmid pHelper (Agilent Technologies, Santa Clara, CA). The recombinant viruses were purified by isolation from two sequential continuous cesium chloride gradients, and the viral titers were determined by qPCR.

AAV vector administration to cultured cells

Differentiation of SH-SY5Y cells was performed as described previously[68] with modifications. Briefly, cells were seeded on collagen type I coated dish (AGC Techno Glass, Haibara, Japan). After 1–2 days, cells were treated with 10 μM retinoic acid (Sigma-Aldrich) in DMEM/F12 supplemented with 1% FBS for over 14 days with changing the medium every 2–3 days. Differentiated SH-SY5Y cells were seeded on collagen type I-coated 6-well plate (AGC Techno Glass) and treated with or without AAV9/3-*modHEXB* or AAV9/3-*HEXB-P2A-HEXA* (0.1–5.0 × 10^5 vg/cell) for 1 week.

Preparation of cell lysates

Cells were washed with ice-cold phosphate-buffered saline (PBS), then removed with a scraper and collected in PBS, followed by centrifugation at 2000 × *g* for 5 min at 4°C. The pellet was resuspended with RIPA buffer (50 mM Tris–HCl (pH 7.6), 150 mM NaCl, 1% Nonidet P40, 0.5% sodium deoxycholate, and 0.1% sodium dodecyl sulfate (SDS)) containing protease inhibitor cocktail (1 μM pepstatin A, 20 μM leupeptin, 1 mM ethylenediaminetetraacetic acid, and 1 mM phenylmethylsulfonyl fluoride). The cell suspensions were sonicated and centrifuged at 12000 × *g* for 15 min at 4°C. The supernatants were collected as cell lysates.

Enzyme assays

β-Hex activities toward 4-methylumbelliferyl-*N*-acetyl-β-D-glucosaminide (MUG, Sigma-Aldrich) and 4-methylumbelliferyl-6-sulfo-*N*-acetyl-β-D-glucosaminide (MUGS; Merck, Darmstadt, Germany) were measured in 0.1 M sodium citrate buffer at pH 4.5 and 4.2, respectively.[69] The protein levels were assayed with the DC Protein Assay (Bio-Rad, Hercules, CA) with bovine serum albumin (BSA, Sigma-Aldrich) as a standard.

Immunoblot analysis

Protein samples were incubated at 100°C for 3 min in SDS sample buffer. The obtained samples were separated on SDS-PAGE and electrotransferred onto Immobilon-P membranes (Merck Millipore) using the Trans-Blot SD Semi-Dry Transfer Cell (Bio-Rad).

The membranes were blocked with Blocking One (Nacalai Tesque, Kyoto, Japan)/tris-buffered saline (TBS) (1:1) or TBS containing 5% skim milk and 0.1% Tween 20 for 1 h at 25°C. Then, they were probed with anti-NAG(A) (1:1000 dilution in blocking buffer), anti-GFP (1:1000), or anti-GAPDH (1:1000) overnight at 4°C. After the membranes were washed with TBS/0.1%Tween 20, the bound antibodies were visualized using horseradish peroxidase (HRP)-linked anti-rabbit or anti-mouse IgG secondary antibody (#7074 and #7076, Cell Signaling Technology, Danvers, MA, 1:1000) depending on the primary antibody. Chemiluminescence signals were detected with Western Lightning Plus-ECL or Ultra (PerkinElmer, Waltham, MA) in a LAS-4000miniEPUV (FUJIFILM, Tokyo, Japan) or ChemiDoc XRS+ system (Bio-Rad).

Immunofluorescence staining

Cells were seeded onto 8-well Lab-Tek chamber slides (Thermo Fisher Scientific) coated with 3 mg/mL atelocollagen (Koken, Tokyo, Japan). Then, 5×10^5 vg/cell AAV9/3-*modHEXB* was added, followed by incubation for 1 week. The cells were fixed with 4% paraformaldehyde/PBS. After washing with PBS, the samples were blocked with 5% goat serum (Cedarlane Labs, Burlington, Canada)/1% BSA/PBS for 1 h at 25°C. Then, they were probed with anti-GM2 antibody (1:50) overnight at 4°C. After washed with 0.1% Tween 20/PBS, the cells were treated with fluorescein isothiocyanate-conjugated anti-mouse IgG+M (1:1000, Abcam, ab47830) and Hoechst33258 (Sigma-Aldrich). The specimens were viewed with LSM700 (Zeiss, Oberkochen, Germany). Fluorescence signal intensities were quantified using ImageJ software.

Vector delivery in mice

AAV9/3-*modHEXB* at 5.8×10^{12}, 1.6×10^{13}, and 2.9×10^{13} vg/kg, as well as AAV9/3-*GFP* at 1.5×10^{13} vg/kg, were injected (0.5 mm caudally from the bregma, 1.0 mm laterally from the central line, and 2.0 mm from the top of the skull) into each ventricle with a two-step needle (Hoshiseido, Tokyo, Japan). After the injection, the needle was held at the site for 1 min to prevent reverse flow.

Vector delivery in non-human primates

The AAV vector administration into non-human primates was performed at TPRC. Non-human primates were anesthetized with 10 mg/kg ketamine hydrochloride (Ketaral, Daiichi Sankyo, Tokyo, Japan) and 1 mg/kg xylazine (Seractal, Bayer Yakuhin, Osaka, Japan) by intramuscular injection. We inserted a cannula into the lumbar cavity by lumbar puncture and collected 1 mL of cerebrospinal fluid. The head was then placed in a supine position with the head lowered 30°, and AAV vector was administered over 5 min. The AAV vector solution was washed with 500 μL of artificial spinal fluid (Artcereb, Otsuka Pharmaceutical Factory, Naruto, Japan), maintained for 15 min, and then the puncture site was compressed. Finally, xylazine was antagonized with an intramuscular injection of 0.5 mg/kg atipamezole (Antisedan, Nippon Zenyaku Kogyo, Koriyama, Japan).

Vector delivery in rats

The *i.t.* single administration via a cannula in the medullary cavity of the thoracic vertebrae was performed with a plastic syringe, an injector, and a syringe pump at a flow rate of 7.5 μL/min. The dosage volume was 17.5 μL/rat (low-dose group) or 53 μL/rat (control and high-dose groups), respectively.

Tissue collection and processing from mice

For tissue extracts, the frozen tissues were thawed and 300 μL of RIPA buffer containing protease inhibitors for each 100 mg of wet weight was added and then homogenized by sonication. After centrifugation at $12000 \times g$ for 15 min at 4°C, the supernatants were collected as tissue extracts.

For lipid extraction, mouse brains were homogenized by adding 100 μL of chloroform/methanol (1:1, by vol.) to 10 mg wet weight, then incubated overnight at 4°C, and the supernatant was used as total lipids.

For tissue sections, the mouse brain was cut in half in the sagittal direction and the spinal cord was cut in the coronal direction. They were embedded in Tissue-Tek O.C.T. compound (Sakura Finetek Japan, Tokyo, Japan), and 10-μm-thick sections were prepared using a CM3050S cryostat (Leica Biosystems, Wetzlar, Germany), mounted on APS glass-coated slides (Matsunami Glass Industry, Kishiwada, Japan), and stored at −80°C. The left brain and spinal cord of non-human primates were fixed in 10% formalin and then sectioned coronally to prepare 10-μm-thick sections.

Immunohistochemistry

The sections of mouse brains were thawed and dried at 25°C for 30 min and then fixed in 4% PFA/PBS at 25°C for 1 h. The sections of non-human primate tissues were deparaffinized using ST5010 Autostainer XL (Leica Biosystems) and were antigen-retrieved in HistoVT One (Nacalai Tesque) for 40 min at 90°C. The sections were washed in PBS and then incubated in the blocking solution (PBS containing 1% BSA and 5% normal goat serum) for 60 min at 25°C. The primary antibody was applied overnight at 4°C, followed by the incubation with a secondary antibody and Hoechst 33258 for 60 min at 25°C in the dark. For TUNEL staining, DeadEnd Colorimetric TUNEL system (Promega, Madison, WI) was used according to the manufacturer's instructions. The treated sections were visualized using LSM700 microscope. The primary antibodies used were as follows: anti-NAG(A) (1:1000 dilution), anti-GM2 (1:20), anti-GFP (1:1000), anti-NeuN (1:200), and anti-CD68 (1:400).

Sliced rat specimens were stained using the VECTASTAIN Elite ABC Kit (Vector Laboratories, Newark, CA). Briefly, the sections were deparaffinized and rinsed with running tap water at 25°C for 5 min, and ion-exchanged water. The sections were soaked in PBS at 25°C for 5 min followed by antigen retrieval buffer (Abcam, ab93684), and processed in an antigen activation device at 110°C for 15 min. After cooling at 25°C, the sections were washed twice with PBS for 5 min and soaked in 0.3% hydrogen peroxide solution to block endogenous peroxidase at 25°C for 30 min. The sections were incubated with Blocking Serum at 25°C for 30 min. Anti-HexB antibody (1:2500) or PBS for negative control was applied at 4°C overnight. The sections were sequentially incubated with biotinylated secondary antibody at 25°C for 30 min, enzyme-labeled complex at 25°C for 30 min, and DAB color developer at 25°C for approximately 1 min. The stained sections were rinsed with running tap water at 25°C for at least 5 min, and nuclear were stained using Tissue-Tek Mayer Hematoxylin For Prisma standard solution reservoir (Sakura Finetek Japan) at 25°C for approximately 1 s. The sections were dehydrated to be transparent and mounted. The treated sections were visualized using Olympus BX43F microscope (Olympus, Hachioji, Japan).

ELISA

The MIP-1α levels in the mouse brain (each 200 μg protein) were measured with Mouse CCL3/MIP-1 alpha ELISA Kit - Quantikine (R&D Systems, Minneapolis, MN) according to the manufacturer's instruction.

To quantify anti-modHexB antibodies in mouse serum, purified recombinant modHexB protein was diluted to 10 μg/mL in PBS and coated onto 96-well ELISA plates (Greiner BIO-ONE) at 100 μL per well, followed by incubation at 4°C overnight. Plates were washed five times with PBS and blocked with 5% skim milk in PBS (200 μL per well) for 2 h at 25°C. Serum samples from untreated SD mice or SD mice that received i.c.v. administration of AAV9/3-*modHEXB* (5.8×10^{12} vg/kg BW at 6 weeks of age) were diluted 1:1000 in blocking buffer, applied at 100 μL per well, and incubated at 4°C overnight. After five washes with 0.1% Tween 20/PBS, HRP-conjugated anti-mouse Ig antibody (1:1000 dilution) was added and incubated for 1 h at 25°C. Plates were washed five times with 0.1% Tween 20/PBS, and bound antibodies were detected using a peroxidase assay kit for ELISA (Sumitomo Bakelite). Absorbance was measured at 450 nm using a microplate reader.

In situ staining for β-hex activity

In situ staining for β-Hex activity using Naphthol AS-BI *N*-acetyl-β-D-glucosaminide (Merck) and pararosaniline (Tokyo Chemical Industry, Tokyo, Japan) was performed as described previously[70] with modifications. Briefly, the sections of mouse brains were thawed and dried at 25°C for 30 min, and then reaction reagent was treated at 37°C for 1 h. After washing with PBS, the treated sections were visualized using BZ-9000 microscope (Keyence, Osaka, Japan).

Thin-layer chromatography

Total lipids extracted from the mouse brain were spotted on a thin-layer chromatography (TLC) plate (Merck) and developed using chloroform/methanol/0.2% calcium chloride (60:40:8.7, by vol.). The plate was soaked in 0.005% primulin/80% acetone and visualized with a ChemiDoc XRS+.

Rota-rod test and lifespan analysis

The abilities of the mice to maintain balance on a rotating cylinder were evaluated by the rota-rod test. Mice were tested for 120 s sessions using MK-610A (Muromachi Kikai, Tokyo, Japan), in which the rotating velocity was increased from 4 to 40 rpm over 120 s, with the latency for falling off in 6 trials/session. Rota-rod test and measurement of the body weight were performed every week from 10 to 16 weeks old. The lifespans were evaluated by the Kaplan-Meier method.

Pathological and histopathological examination in non-human primates

For pathological and histopathological examinations, non-human primates were anesthetized by intramuscular injection of ketamine hydrochloride. Body weight was measured, followed by urine and blood collection. Cerebrospinal fluid was then collected, after which animals were euthanized by intravenous administration of sodium pentobarbital. Subsequently, systemic perfusion was performed with 1,000 mL of heparinized physiological saline to remove blood from the circulation. After perfusion, a complete gross anatomical examination was conducted and macroscopic findings were recorded. The brain was removed, and the left hemisphere was fixed in 10% formalin for histopathological analysis. The right hemisphere was dissected to isolate major anatomical regions and snap-frozen for subsequent biochemical analyses. Representative samples of major organs were also collected and fixed in 10% formalin. The eyeballs were fixed in a glutaraldehyde-containing fixative. Formalin-fixed tissues were processed by routine methods, embedded in paraffin, sectioned, and stained with hematoxylin and eosin. Histopathological evaluation was performed on the central nervous system as well as on major peripheral organs. For frozen tissue sections, the fixed left brain and spinal cord were sectioned coronally to prepare 10-μm-thick sections. For immunoblot analyses, endogenous HexA derived from non-human primates in tissue extracts was separated using anion exchange chromatography based on the difference in isoelectric points. Briefly, Vivapure Q Mini H columns (Sartorius, Göttingen, Germany) were equilibrated to pH 6.0 with 10 mM sodium phosphate buffer. Tissue extract was added to the column to adsorb endogenous HexA onto an anion exchange carrier. The spin column was then centrifuged at 4°C, $2000 \times g$, for 3 min, and passed through fraction containing modHexB was collected.

Hematological and blood chemistry analyses using non-human primates

Complete blood count and blood chemistry analyses of samples were performed using pocH-100iV Diff (Sysmex Corporation, Kobe, Japan) and FUJIFILM DRI-CHEM 7000V (FUJIFILM), respectively.

General toxicity studies in accordance with GLP using normal rats

Mortality and clinical signs of all rats were observed twice on Day 0 (before and after dosing) and once a day hereafter. Rats found dead were immediately weighed and necropsied. The organs and tissues were weighed and fixed in neutral buffered 10% formalin, and these were examined histopathologically. All animals were weighed using an electronic balance on the day of dosing (before dosing), once a week until necropsy, and on the day before and the day of necropsy. Daily food consumption of all animals was calculated by measuring the amounts of food given and residue with electronic balance once a week until necropsy.

For ophthalmology, at 26 weeks after administration, for half of the animals in each group of the toxicity study unit, the anterior portions of the eye (the orbital region, palpebra, conjunctiva, cornea, sclera, anterior chamber, and iris) were examined with a portable slit-lamp (SL-15, Kowa, Nagoya, Japan). In addition, the intermediate optic media (the lens and vitreous body) and fundus (the optic papilla, retina, and choroid) were examined with a binocular indirect ophthalmoscope (IO-α LED BP-II, Neitz Instruments, Tokyo, Japan) after the instillation of a mydriatic agent (Mydrin P, Santen Pharmaceutical, Osaka, Japan).

For hematology, all surviving animals were fasted from the evening on the day before necropsy. The blood was collected from the abdominal aorta under anesthesia of Isoflurane on the necropsy day. Prothrombin time and activated partial thromboplastin time were determined using plasma obtained from the blood anticoagulated with 3.2% sodium citrate, followed by centrifugation at 3000 rpm for 15 min at 4°C. Other parameters were determined using plasma obtained from the blood anticoagulated with EDTA-2K. The parameters were measured using automatic blood cell counter (XT-2000iV, Sysmex Corporation) and automatic blood coagulation counter (CA-1500, Sysmex Corporation).

For blood chemistry, we used plasma anticoagulated with sodium heparin (Mochida Pharmaceutical, Tokyo, Japan) for aspartate aminotransferase and lactate dehydrogenase, and serum for other than these two enzymes. The parameters were measured using automatic clinical chemistry analyzer (JCA-BM6010, JEOL, Akishima, Japan).

For urinalysis, urine was collected using a metabolism cage. Fresh urine was collected within 2 h after urinary excretion and parameters including pH, protein, glucose, ketone bodies, urobilinogen, bilirubin, occult blood, sediments, and color were determined using test paper method (Multistix, Siemens Healthcare Diagnostics, Tokyo, Japan), microscopy after Sternheimer-Malbin (SM) stain, and color gross inspection. During the collection of fresh urine, food was not given but drinking water was available freely. The 24-h stock urine was used for examination of the parameters including volume, specific gravity, sodium, potassium, and chloride using volumetric cylinder, Uricometer (T2-SE, Atago, Tokyo, Japan), and Automatic clinical chemistry analyzer (JCA-BM6010, JEOL).

For pathological examination, all animals at 26 weeks after administration were fasted from the evening on the day before necropsy. Then, animals were euthanized by bleeding from the abdominal aorta after blood collection under isoflurane anesthesia, and animals were necropsied. The organs were weighed and the relative organ weights to body weight were calculated based on body weights on the necropsy day. Bilateral organs were weighed together. The eyeballs (including the optic nerve) were fixed in Davidson's fixative and the testes were in modified Davidson fluid. The other organs and tissues were fixed in neutral buffered 10% formalin. The fixed organs and tissues were embedded, thin-sectioned, stained with hematoxylin and eosin, and examined microscopically. Bone tissues were decalcified.

For anti-AAV9/3 antibody measurement, 0.5 mL of the blood was collected from the cervical vein without anesthesia. The collected blood was moved into the MiniCollect Blood Collection Tubes (Clot activator/serum separator, Greiner Bio-One, Kremsmünster, Austria) and allowed to stand at 25°C for 30 min or more. The collected blood was centrifuged at 1800 × g for 15 min at 4°C to obtain serum samples. AAV9/3 antibody in serum was measured by ELISA using analysis reagents for AAV9/3 antibody (Kainos Laboratories, Tokyo, Japan) and SpectraMax M4 (Molecular Devices, San Jose, CA). The presence or absence of anti-AAV9/3 antibodies in serum of each animal was judged based on the cut point calculated from the assay results of the control group.

Biodistribution studies using normal rats

For genome titer measurement, animals at 13 and 26 weeks after administration were euthanized by bleeding from the abdominal aorta under isoflurane anesthesia, and the organs were collected and weighed. On the sampling day, 0.3 mL of the blood, a part of feces, and urine excreted for 24 h from the day before sampling, were collected. Saliva was collected by dropping the quinine hydrochloride solution at a concentration of 10^{-2} mol/L in the oral cavity. The cryopreserved samples were thawed and DNA was extracted from these samples using QIAamp DNA Blood Mini Kit, QIAamp 96 DNA QIAcube HT Kit, and QIAamp Fast DNA Stool Mini Kit (QIAGEN, Hilden, Germany).

Quantification of AAV vector genome copies

Vector genome titers of AAV9/3-*modHEXB* were quantified by qPCR. For non-human primate tissues, genomic DNA was extracted using a phenol-chloroform-based method. Briefly, tissue samples were homogenized in 300 μL of DNA extraction buffer (50 mM Tris-HCl, 20 mM EDTA, 2% SDS) using a disposable pestle, followed by addition of proteinase K (3 μL) and incubation at 60°C overnight. After digestion, equal volumes of TE-saturated phenol and chloroform:isoamyl alcohol (24:1) were added, vortexed, and centrifuged at 12,000 × *g* for 10 min at 4°C. The aqueous phase was transferred and further extracted with chloroform, followed by centrifugation.

Sodium chloride (4 M) was added to the supernatant, centrifuged, and DNA was precipitated with absolute ethanol. The DNA pellet was washed with 75% cold ethanol, air-dried, and resuspended in 100 μL of nuclease-free water. DNA concentration was determined using a NanoDrop spectrophotometer. Quantification of AAV vector genomes was performed by iQ SYBR Green Supermix (Bio-Rad). Serial dilutions (10- to 100,000-fold) of AAV9/3-*modHEXB* vector stock (2.2×10^9 vg/mL) were used to generate standard curves. Each reaction contained 5 μL of SYBR Green Supermix, 500 nM each of forward primer (5′-ATTGCTTCCCGTATGGCTTTCA-3′) and reverse primer (5′-TCAGCAAACACAGTGCACACCA-3′), and 4.8 μL of template DNA. PCR was performed with an initial denaturation at 95°C for 3 min, followed by 40 cycles of 95°C for 15 s and 60°C for 1 min.

For rat samples, AAV9/3-*modHEXB* vector genome titer was measured using TaqPath qPCR Master Mix and Quant Studio 5 (Thermo Fisher Scientific). The primers and probe were as follows: 5′-TACGAGGAATTCGAGTCCTG-3′, 5′-AAAGGTGCCAGAGGGCTCAGAC-3', and/56-FAM/TGGGGAAAA/ZEN/GGTCAGAAAGACCTC/31ABkFQ/. The PCR conditions were 50°C for 2 min and 95°C for 20 s, followed by 40 cycles of denaturation at 95°C for 15 s and annealing/extension at 60°C for 1 min. The genome titers were calculated using standard curves generated with serially diluted *modHEXB* oligo. The sequence was as follows: AC CAAATGATGTCCGTATGGTGATTGAATATGCCAGATTACGAGGAATTCGAGTCCTGCCAGAATTTGATACCCCTGGGCATACACTA TCTTGGGGAAAAGGTCAGAAAGACCTCCTGACTCCATGTTACAGTGGGTCTGAGCCCTCTGGCACCTTTGGACCTATAAACCCTA CTCTGAATACAACATACAGCTTCCTTACTACATTTTTCAAAGAAATTAGTGAGGTGTTTCCAGATCAATTCATTCATTTGGGAGGAG ATGAAGTGGAATT.

QUANTIFICATION AND STATISTICAL ANALYSIS

All data are expressed as the mean ± SEM. *p*-values <0.05 were considered statistically significant. For experiments using rats, statistical analysis was performed using the SAS System ver. 9.4 (SAS Institute, Cary, NC). Data were analyzed for homogeneity using Bartlett's test. ANOVA followed by Dunnett's and Steel's tests were used for homogeneous and heterogeneous data, respectively. For other experiments, statistical analyses were performed using Prism ver. 8.43 (GraphPad Software; San Diego, CA). The paired two-tailed *t* test was used to compare two groups, while ANOVA followed by Tukey's test was used to compare three or more groups. Statistical significance in figures is indicated as follows: $^{*}p < 0.05$, $^{**}p < 0.01$, $^{***}p < 0.001$, $^{\dagger}p < 0.05$, $^{\dagger\dagger}p < 0.01$, $^{\dagger\dagger\dagger}p < 0.001$. The symbols indicate comparisons between groups as specified in the corresponding figure legends.

Cell Reports Medicine, Volume 7

Supplemental information

AAV-based gene therapy with modified *HEXB* confers lasting therapeutic benefits in GM2 gangliosidosis models

Keisuke Kitakaze, Yukiya Ohnishi, Daisuke Tsuji, Ryosuke Watanabe, Nijiho Kamori, Yuko Katakai, Hiroaki Shibata, Sota Yoshizawa, Mika Ito, Naomi Takino, Shin-ichi Muramatsu, and Kohji Itoh

Supplementary information

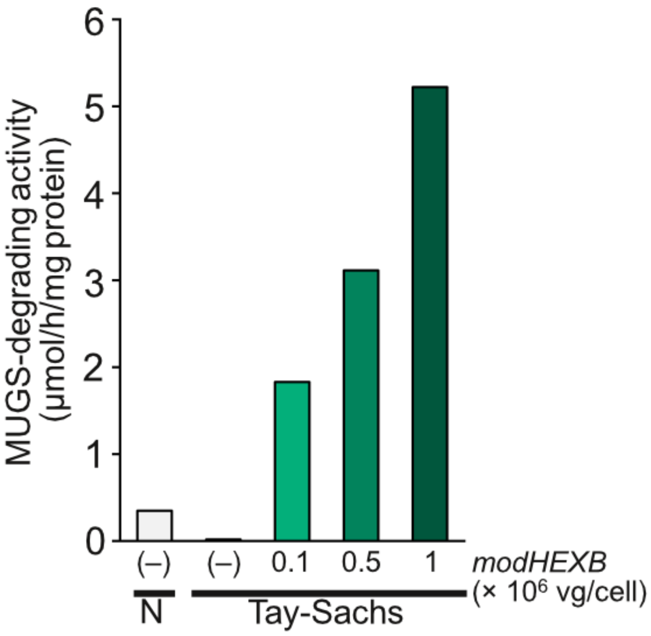

Fig. S1. Restoration of β-Hex activity in cultured human neuronal cells differentiated from iPS cells derived from patients with Tay-Sachs disease. Intracellular MUGS-degrading β-Hex activity after transduction with AAV9/3-*modHEXB* (n =1). N: normal.

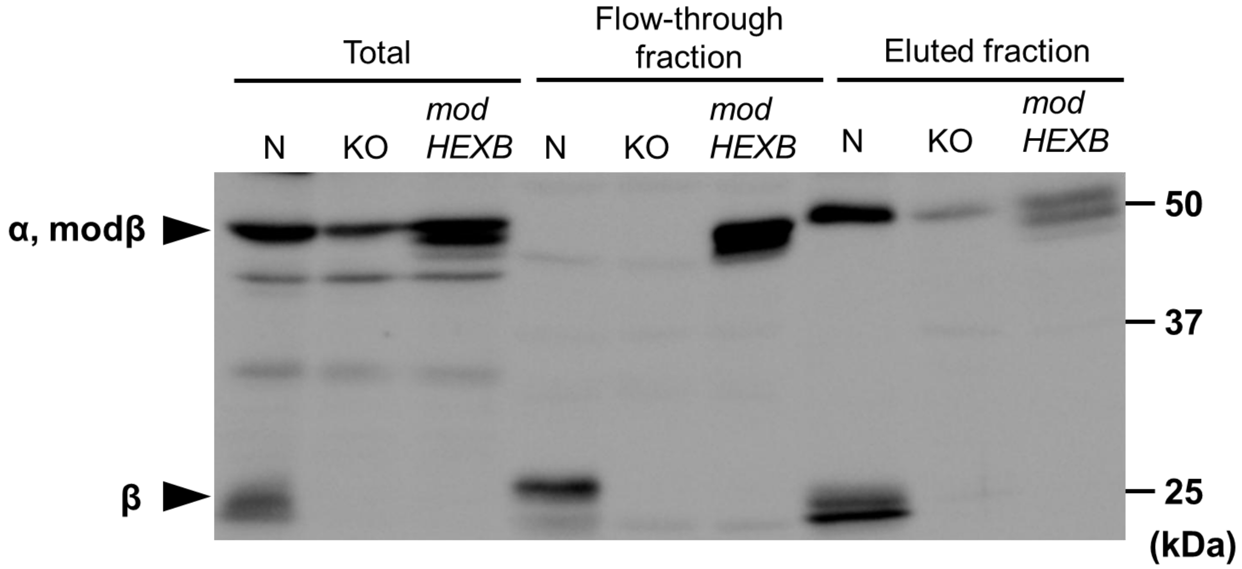

Fig. S2. Isoenzyme composition of β-hexosaminidase analyzed by Q-column chromatography in SH-SY5Y cells. Cell extracts from normal SH-SY5Y cells (N), *HEXB* knockout (KO) SH-SY5Y cells, and *HEXB* KO SH-SY5Y cells transduced with AAV9/3-*modHEXB* (*modHEXB*) were subjected to Q-column anion-exchange chromatography. The flow-through fractions contained endogenous HexB (β/β) and/or transgene-derived modHexB (modβ/modβ), whereas the eluted fractions contained HexA (α/β) and HexS (α/α). Fractions were analyzed by SDS–PAGE followed by immunoblotting using an anti-HexA antibody.

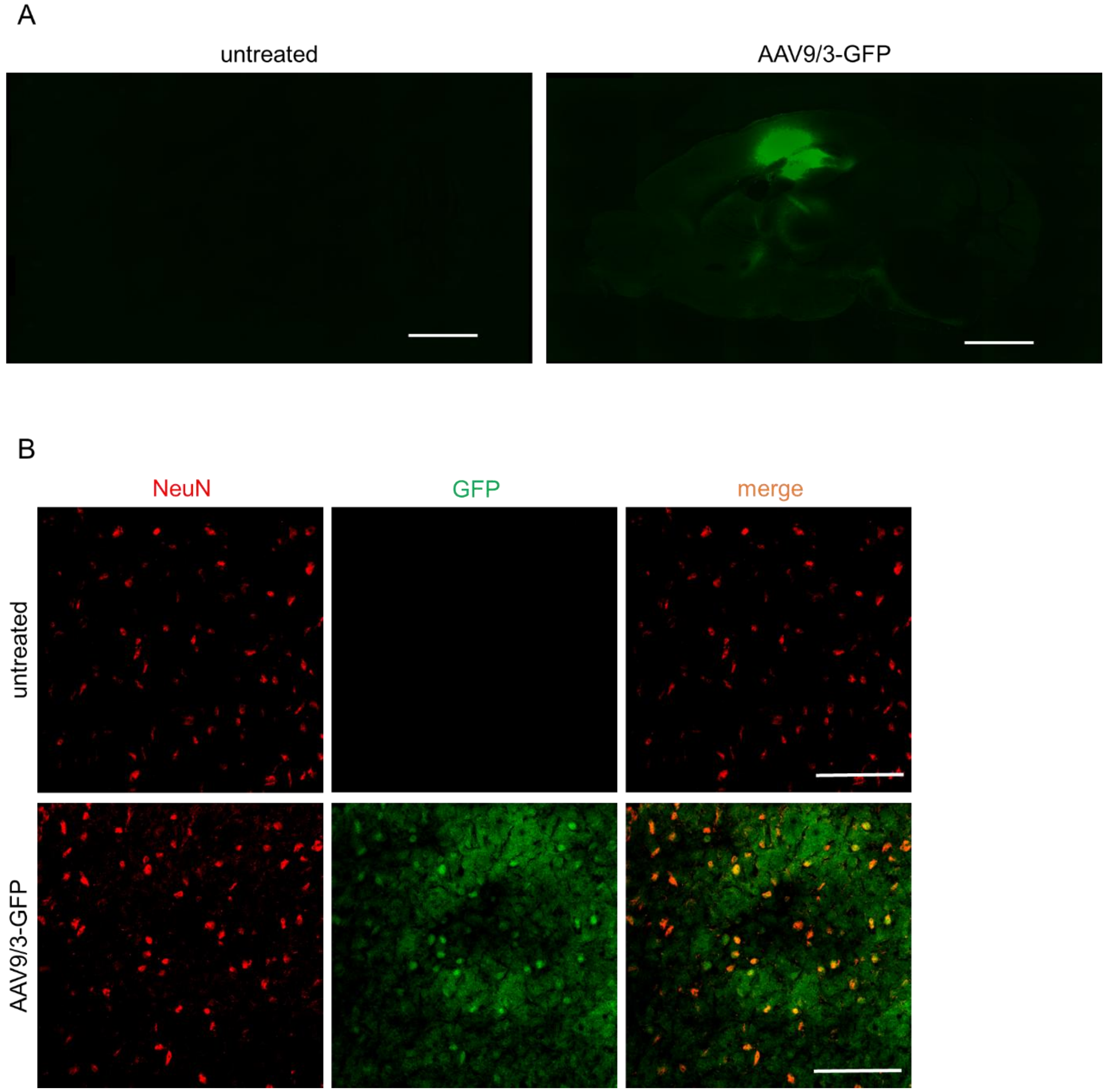

Fig. S3. Immunohistochemical analyses of the brain from wild-type mice intracerebroventricularly administered AAV9/3-GFP. (**A**) GFP in sagittal sections of the whole brain. Green: GFP. Scale bars: 2 mm. (**B**) Co-localization of NeuN and GFP in cerebral. Red: NeuN, green: GFP. Scale bars: 2 mm.

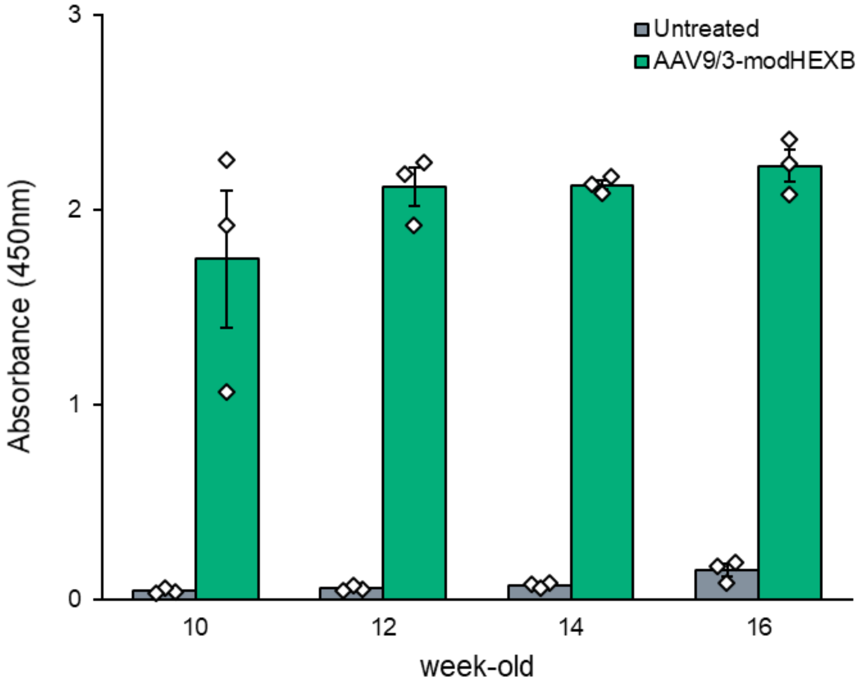

Fig. S4. Detection of anti-modHexB antibodies in SD mice by ELISA. Serum anti-modHexB antibody levels were measured by ELISA using purified recombinant modHexB as the coating antigen. Sera were collected from untreated SD mice and from SD mice treated with AAV9/3-*modHEXB* at 6 weeks of age (5.8×10^{12} vg/kg BW), diluted 1:1,000. Bound antibodies were detected using an HRP-conjugated anti-mouse Ig secondary antibody and quantified by absorbance at 450 nm. Data are shown as mean ± SEM (n = 3).

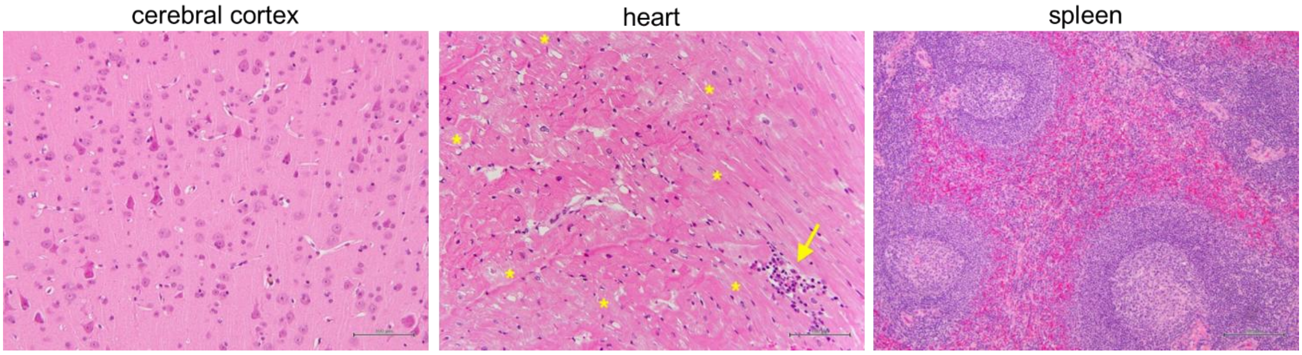

Fig. S5. Representative histopathological findings in non-human primates following *i.t.* administration of AAV9/3-*modHEXB* (2.0×10^{12} vg/kg). Left panel: Cerebral cortex (parietal lobe) from animal #1 showing mild gliosis with increased microglial cells, accompanied by a diffuse increase in neuronophagia and eosinophilic neurons. Middle panel: Heart from animal #3 demonstrating focal contraction band necrosis of cardiomyocytes in the left ventricular wall (outlined area), without progression to liquefactive necrosis. Multifocal, minimal mononuclear cell infiltration associated with localized cardiomyocyte degeneration is also observed (yellow arrows). Right panel: Spleen from animal #3 showing diffuse lymphoid follicular hyperplasia. Scale bars, 100 µm.

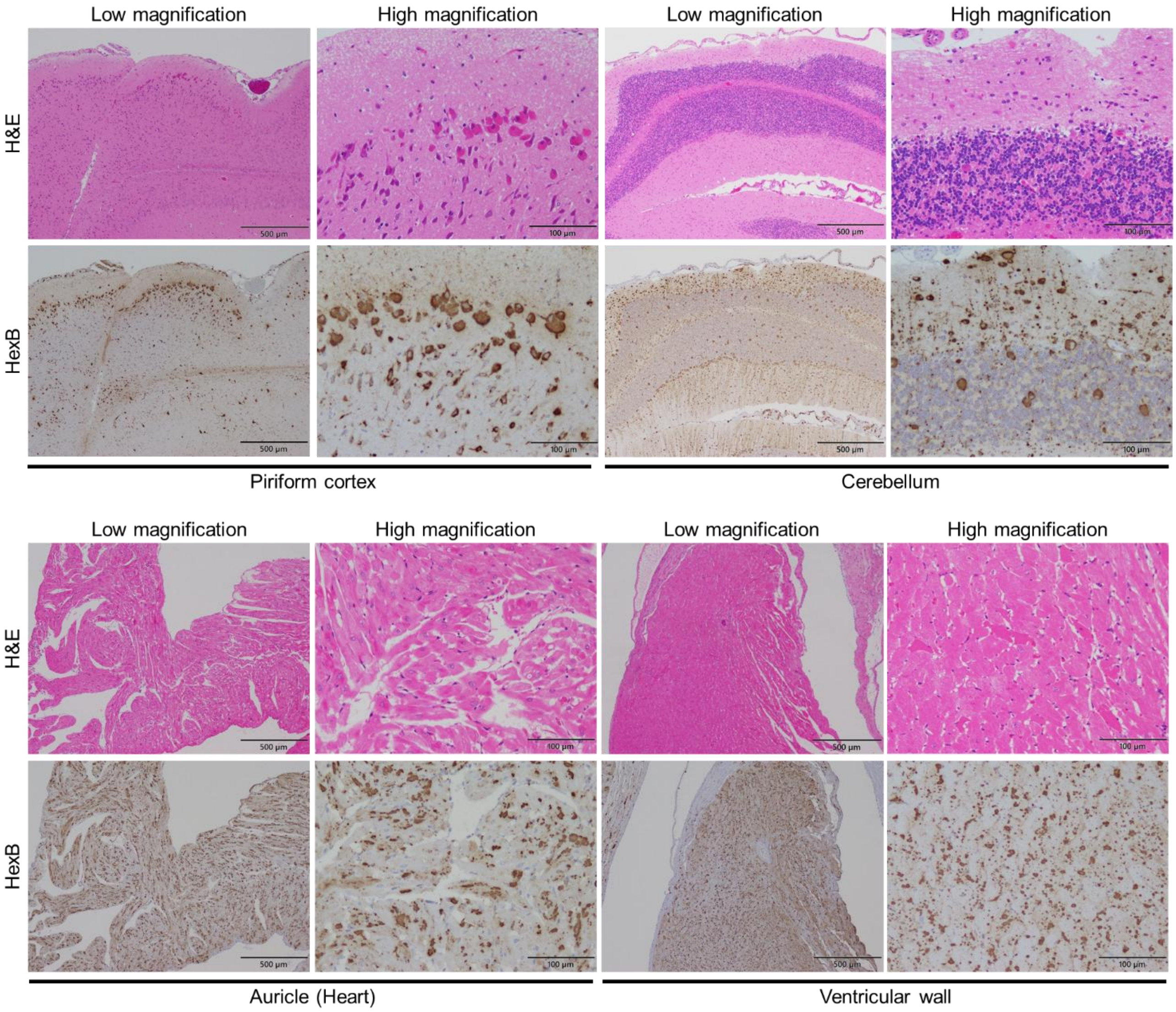

Fig. S6. H&E staining and immunohistochemical analysis of wild-type rat tissues following *i.t.* administration of the AAV9/3-*modHEXB*. Representative images of the high-dose group (9.9×10^{11} vg/g brain) are shown. Scale bars indicate 500 μm (low magnification) and 100 μm (high magnification), respectively.

Table S1. Quantification of fluorescent signal intensity

		WT	SD	SD + *modHEXB*
CD68/Nuclei	Cerebrum	1.87	16.03	3.59
	Hippocampus	0.18	5.52	4.44
	Cerebellum	0.10	5.73	0.78
NeuN/TUNEL/Nuclei	Cerebrum	0.39	13.79	0.61
	Hippocampus	0.10	4.56	1.18
	Cerebellum	0.99	6.44	0.04

CD68-positive and NeuN/TUNEL-positive areas were quantified and normalized to the nuclear area, and the resulting values (%) are shown.

Table S3. Hematology and blood chemistry of non-human primates

	Day0<br>n = 5	Day28<br>n = 5	Day84<br>n = 5	Ref data<br>(Lab Anim Res 2019)	Ref data<br>(Animals 2023)
RBC (10^4/μL)	594 ± 15	595 ± 16	592 ± 14	416–732	459–660
WBC (10^2/μL)	113.8 ± 19.1	132.0 ± 16.6	118.4 ± 18.0	30.4–184.0	62.7–254.8
Ht (%)	40.0 ± 1.1	40.8 ± 1.2	41.2 ± 1.0	31.6–57.2	33–42
Hb (g/dL)	11.7 ± 0.4	11.7 ± 0.3	11.5 ± 0.3	9.1–17.0	
MCH (pg)	19.6 ± 0.3	19.6 ± 0.3	19.4 ± 0.5	17.1–24.2	
MCV (fL)	67.3 ± 0.6	68.7 ± 1.0	69.6 ± 0.9 *	63.8–82.9	59.2–76.4
MCHC (g/dL)	29.2 ± 0.2	28.6 ± 0.1 *	27.8 ± 0.4 **	23.6–33.9	
Plt (10^4/μL)	32.3 ± 4.6	29.4 ± 2.7	27.7 ± 2.1	28.1–67.2	20.8–58.4
W-SCR (%)	39.5 ± 7.8	44.3 ± 6.6	44.2 ± 7.7		
W-LCR (%)	60.5 ± 7.8	55.7± 6.6	55.8 ± 7.7		
W-SCC (10^2/μL)	41.0 ± 9.0	58.4 ± 11.5 **	51.6 ± 12.1		
W-LCC (10^2/μL)	72.8 ± 22.2	73.6 ± 12.6	66.8 ± 14.5		
RDW-SD (fL)	24.0 ± 0.5	26.0 ± 0.8 *	25.7 ± 0.7 *		
PDW (fL)	11.4 ± 0.9	11.1 ± 0.6	11.2 ± 0.6		10.7–18.0
MPV (fL)	10.4 ± 0.5	10.2 ± 0.3	10.1 ± 0.4	7.6–16.3	9.6–13.4
P-LCR (%)	28.0 ± 4.3	26.9 ± 3.0	25.9 ± 3.2		22.4–50.2
AST (U/L)	59.8 ± 2.3	74.4 ± 14.8	72.0 ± 26.2	13–106	23.9–91.4
ALT (U/L)	62.6 ± 6.1	112.6 ± 36.5	100.0 ± 37.7	1.0–56.0	11.8–77.8
ALP (U/L)	1809 ± 191	1903 ± 190	2110 ± 264	158–741	185–1327
CPK (U/L)	248 ± 32	395 ± 227	574 ± 202		
LDH (U/L)	1285 ± 153	1123 ± 122	714 ± 211	31–467	327–1173
γ-GTP (U/L)	98 ± 20	102 ± 23	103 ± 24	1–199	38–157
Glu. (mg/dL)	36 ± 7	44 ± 9	48 ± 8	26–130	
T.Cho. (mg/dL)	105 ± 13	108 ± 12	102 ± 10	37–203	48–217
F.Cho. (mg/dL)	23.6 ± 2.9	24.6 ± 2.5	24.6 ± 2.4		
TG (mg/dL)	36 ± 5	46 ± 6 *	66 ± 11 **	9–213	
TP (g/dL)	6.9 ± 0.1	7.0 ± 0.1	7.1 ± 0.1	4.6–8.1	5.5–7.1
Alb. (g/dL)	4.4 ± 0.1	4.5 ± 0.1	4.4 ± 0.2	2.2–4.8	2.9–4.7
A/G	1.80 ± 0.08	1.75 ± 0.12	1.64 ± 0.12		
BUN (mg/dL)	15.9 ± 1.6	18.1 ± 0.9	16.3 ± 0.8	7.5–28.0	13.7–30.8
Fe (μg/dL)	201 ± 12	211 ± 9	176 ± 29		50–198
CRP (mg/dL)	0.0 ± 0.0	0.1 ± 0.0 **	0.3 ± 0.2		
Crea. (mg/dL)	0.58 ± 0.02	0.56 ± 0.05	0.50 ± 0.00 *	0.10–1.10	0.41–0.94
T.Bil. (mg/dL)	0.12 ± 0.02	0.14 ± 0.02	0.14 ± 0.04	0.10–2.30	0.01–0.22
D.Bil. (mg/dL)	0.00 ± 0.00	0.00 ± 0.00	0.00 ± 0.00	0.10–0.80	
I.Bil. (mg/dL)	0.12 ± 0.02	0.12 ± 0.02	0.14 ± 0.04		
Na (mEq/L)	146.6 ± 1.5	146.6 ± 0.4	146.0 ± 1.6		138.5–148.6
K (mEq/L)	4.7 ± 0.3	3.7 ± 0.1 *	3.7 ± 0.1 *		3.1–4.4
Cl (mEq/L)	108.2 ± 0.7	107.0 ± 0.5	112.0 ± 1.4		100.9–110.9
P (mg/dL)	5.1 ± 0.7	5.1 ± 0.5	4.9 ± 0.4	1.1–6.6	2.6–7.6
Ca (mg/dL)	9.5 ± 0.1	9.5 ± 0.1	9.7 ± 0.1	6.2–9.9	8.6–10.8

Values are the mean ± S.E.M.,

Paired t-test was performed to compare day 0 with day 28 and 84 [* p <0.05, ** p <0.01, *** p <0.001].

Table S5. Histopathological findings in non-human primates

Organ / Tissue	Subject No.				
	#1	#2	#3	#4	#5
Cerebrum	+[(a)]	–	+[(a)]	–	–
Brainstem	–	–	–	–	–
Cerebellum	–	–	–	–	–
Cervical spinal cord	–	–	–	–	–
Thoracic spinal cord	–	–	–	–	–
Lumbar spinal cord	–	–	–	–	–
Heart	–	+[(b)]	+[(c), (d)]	–	+[(d)]
Lung	+[(e)]	+[(f)]	–	–	+[(g), (f)]
Liver	–	–	–	–	–
Gallbladder	–	–	–	–	Not examined
Kidney	–	–	–	–	–
Urinary bladder	–	–	–	–	–
Spleen	+[(h)]	–	+[(h)]	–	+[(h)]
Pancreas	–	–	–	–	–
Adrenal gland	–	–	–	–	–
Stomach	+[(i)]	–	–	+[(i)]	–
Small intestine	–	–	–	–	–
Large intestine	+[(i)]	–	–	+[(i)]	+[(i)]
Submandibular lymph node	–	+[(h)]	+[(h), (j)]	+[(h)]	–
Tonsil	–	–	–	–	–
Sciatic nerve	–	–	–	–	–
Uterus	–	–	–	–	–
Ovary	+[(k)]	+[(k)]	–	+[(k)]	–
Skin (dorsal)	–	–	–	–	–
Skeletal muscle (biceps brachii)	–	–	–	–	–
Skeletal muscle (femoral)	–	–	–	–	–
Eyeball	–	–	–	–	–
Inner ear	–	–	–	–	–

－: no abnormality observed, +: mild, ++: moderate, +++: severe

Not examined: tissue not present on the histological section

(a) Gliosis, characterized by diffuse cortical microgliosis, accompanied by an increased frequency of neuronophagia and an increased number of eosinophilic neurons.

(b) Cardiomyocyte contraction band necrosis, focal, located in the interventricular septum and right ventricle adjacent to the coronary artery, without progression to cardiomyocyte liquefactive necrosis.

(c) Cardiomyocyte contraction band necrosis, focal and localized in the left ventricular wall, without progression to cardiomyocyte liquefactive necrosis.

(d) Mononuclear cell infiltration, consisting of multifocal minute lesions with focal cardiomyocyte degeneration/fragmentation.

(e) Foamy macrophage aggregation, multifocal and intra-alveolar.

(f) Mononuclear cell infiltration, multifocal, minute, and interstitial.

(g) Foamy macrophage aggregation, intra-alveolar.

(h) Lymphoid follicular hyperplasia, diffuse in distribution.

(i) Lymphoid follicular hyperplasia, localized within the mucosa.

(j) Extramedullary hematopoiesis, of granulocytic lineage.

(k) Mineral deposition, multifocal and focally distributed, scattered in the ovarian cortex.

Table S7. Organ weights

	Male			Female		
	Saline	AAV9/3-*modHEXB*, Low-dose	AAV9/3-*modHEXB*, High-dose	Saline	AAV9/3-*modHEXB*, Low-dose	AAV9/3-*modHEXB*, High-dose
	n = 10	n = 10	n = 9	n = 9	n = 10	n = 10
Absolute weight						
Brain (g)	2.17±0.10	2.12 ± 0.09	2.23 ± 0.09	1.96 ± 0.09	1.98 ± 0.08	1.95 ± 0.14
Pituitary gland (mg)	12.4 ± 1.1	11.2 ± 1.5	12.2 ± 2.4	17.6 ± 5.1	17.0 ± 4.1	16.4 ± 3.8
Thyroid glands (mg) 1)	27.1 ± 3.7	23.7 ± 3.7	26.1 ± 2.3	20.2 ± 4.0	21.3 ± 5.0	20.5 ± 4.7
Adrenal glands (mg)	57 ± 8	51 ± 5 *	65 ± 13	62 ± 8	69 ± 14	62 ± 8
Heart (mg)	1670 ± 165	1611 ± 259	1688 ± 233	956 ± 120	1023 ± 61	998 ± 131
Lung (g) 2)	1.66 ± 0.12	1.66 ± 0.15	1.72 ± 0.16	1.14 ± 0.11	1.21 ± 0.09	1.18 ± 0.11
Thymus (mg)	169 ± 38	193 ± 40	170 ± 49	143 ± 46	171 ± 28	165 ± 32
Spleen (mg)	917 ± 195	937 ± 194	964 ± 226	604 ± 94	627 ± 123	533 ± 132
Submandibular glands (mg) 3)	591 ± 76	642 ± 93	658 ± 137	392 ± 43	448 ± 70	380 ± 72
Liver (g)	18.12 ± 3.15	15.35 ± 4.21	16.14 ± 2.49	7.68 ± 1.06	8.43 ± 1.34	8.30 ± 1.40
Kidneys (g)	3.67 ± 0.40	3.34 ± 0.55	3.66 ± 0.43	1.94 ± 0.18	2.15 ± 0.28	2.05 ± 0.26
Testes (g)	3.60 ± 0.37	3.46 ± 0.22	3.53 ± 0.20	-	-	-
Epididymides (mg)	1409 ± 124	1412 ± 157	1331 ± 127	-	-	-
Seminal vesicle (mg)	1942 ± 357	1994 ± 230	2060 ± 273	-	-	-
Prostate (mg)	958 ± 176	888 ± 289	1114 ± 164	-	-	-
Ovary (mg)	-	-	-	105 ± 22	120 ± 15	127 ± 26
Uterus (mg)	-	-	-	694 ± 162	748 ± 164	690 ± 228
Relative weight						
Brain (g%)	0.308 ± 0.027	0.338 ± 0.042	0.331 ± 0.039	0.611 ± 0.074	0.588 ± 0.080	0.575 ± 0.056
Pituitary gland (mg%)	1.74 ± 0.15	1.77 ± 0.18	1.81 ± 0.37	5.42 ± 1.48	5.04 ± 1.29	4.78 ± 0.91
Thyroid glands (mg%) 1)	3.82 ± 0.55	3.75 ± 0.67	3.87 ± 0.54	6.31 ± 1.62	6.30 ± 1.64	5.95 ± 1.07
Adrenal glands (mg%)	8.02 ± 1.11	8.06 ± 1.42	9.56 ± 1.92	19.26 ± 3.55	20.34 ± 4.55	18.26 ± 2.40
Heart (mg%)	235 ± 13	253 ± 20	248 ± 23	296 ± 34	303 ± 39	292 ± 28
Lung (g%) 2)	0.235 ± 0.025	0.262 ± 0.018	0.255 ± 0.028	0.356 ± 0.047	0.358 ± 0.055	0.347 ± 0.026
Thymus (mg%)	23.6 ± 4.2	30.6 ± 6.6 *	24.9 ± 5.7	45.6 ± 18.4	51.0 ± 11.2	48.5 ± 9.8
Spleen (mg%)	129 ± 25	146 ± 14	143 ± 35	187 ± 28	186 ± 44	155 ± 32
Submandibular glands (mg%) 3)	84 ± 14	102 ± 17 *	97 ± 19	122 ± 20	132 ± 23	111 ± 12
Liver (g%)	2.54 ± 0.28	2.38 ± 0.36	2.36 ± 0.15	2.37 ± 0.18	2.46 ± 0.20	2.42 ± 0.23
Kidneys (g%)	0.516 ± 0.036	0.523 ± 0.040	0.539 ± 0.045	0.603 ± 0.067	0.632 ± 0.070	0.597 ± 0.035
Testes (g%)	0.506 ± 0.029	0.551 ± 0.073	0.522 ± 0.044	-	-	-
Epididymides (mg%)	199 ± 23	223 ± 23 *	197 ± 23	-	-	-
Seminal vesicle (mg%)	276 ± 61	315 ± 25	304 ± 42	-	-	-
Prostate (mg%)	136 ± 30	140 ± 50	166 ± 32	-	-	-
Ovary (mg%)	-	-	-	32.8 ± 8.0	35.4 ± 5.5	37.2 ± 6.0
Uterus (mg%)	-	-	-	217 ± 63	224 ± 68	201 ± 56
Body weight (g%)	711 ± 61	638 ± 93	680 ± 71	324 ± 37	343 ± 52	344 ± 50

Values are the mean ± S.D.

1) with parathyroid glands, 2) with bronchus, 3) with sublingual glands

Dunnett's/Steel's test was performed to compare the Control (Saline) group with each AAV9/3-*modHEXB* group [* p <0.05].

Table S8. Ophthalmologic examination

	Male			Female		
	Saline<br>n = 5	AAV9/3-*modHEXB*, Low-dose<br>n = 5	AAV9/3-*modHEXB*, High-dose<br>n = 5	Saline<br>n = 5	AAV9/3-*modHEXB*, Low-dose<br>n = 5	AAV9/3-*modHEXB*, High-dose<br>n = 5
Appearance	0	0	0	0	0	0
Eyelid	0	0	0	0	0	0
Conjunctiva	0	0	0	0	0	0
Cornea	0	0	0	0	0	0
Sclera	0	0	0	0	0	0
Anterior chamber	0	0	0	0	0	0
Iris	0	0	0	0	0	0
Lens	0	0	0	0	0	0
Vitreous	0	0	0	0	0	0
Fundus: optic disc, retina, choroid	0	0	0	0	0	0

Numerals represent the number of animals with the findings.

Table S9. Hematology

	Male			Female		
	Saline<br>n = 10	AAV9/3-*modHEXB*, Low-dose<br>n = 10	AAV9/3-*modHEXB*, High-dose<br>n = 9	Saline<br>n = 9	AAV9/3-*modHEXB*, Low-dose<br>n = 10	AAV9/3-*modHEXB*, High-dose<br>n = 10
RBC (10^4/μL)	899 ± 22	908 ± 56	871 ± 77	794 ± 80	808 ± 67	807 ± 32
WBC (10^2/μL)	90.1 ± 21.7	118.2 ± 37.2	91.4 ± 36.1	66.8 ± 34.4	61.0 ± 28.4	56.8 ± 16.1
Ht (%)	42.6 ± 1.8	41.8 ± 2.0	41.5 ± 3.5	41.7 ± 2.9	40.9 ± 1.5	41.5 ± 1.4
Hb (g/dL)	15.7 ± 0.7	15.6 ± 0.8	15.3 ± 1.7	15.2 ± 1.4	15.1 ± 0.7	15.4 ± 0.5
MCH (pg)	17.5 ± 0.7	17.2 ± 0.7	17.5 ± 0.6	19.2 ± 0.6	18.7 ± 0.9	19.1 ± 0.4
MCV (fL)	47.4 ± 1.8	46.2 ± 2.6	47.7 ± 1.2	52.7 ± 2.5	50.9 ± 3.6	51.4 ± 1.6
MCHC (g/dL)	36.9 ± 0.5	37.3 ± 0.7	36.6 ± 1.6	36.4 ± 1.0	36.8 ± 0.9	37.1 ± 0.5
Ret (%)	3.79 ± 0.55	3.41 ± 1.13	4.96 ± 3.79	4.62 ± 4.12	3.93 ± 1.54	3.29 ± 0.51
Ret (10^4/μL)	34.01 ± 4.46	30.58 ± 8.36	40.73 ± 23.09	34.12 ± 23.25	30.96 ± 9.18	26.46 ± 3.54
Plt (10^4/μL)	126.0 ± 10.9	144.0 ± 16.1 *	138.5 ± 14.3	113.0 ± 15.1	116.1 ± 6.3	111.9 ± 13.6
PT (s)	14.7 ± 1.3	16.1 ± 1.7	14.7 ± 1.5	12.2 ± 0.6	12.5 ± 0.6	12.2 ± 0.3
APTT (s)	20.4 ± 2.2	21.8 ± 2.3	20.6 ± 1.9	17.1 ± 1.0	17.1 ± 0.6	17.0 ± 0.6
Differential leukocyte counts						
Baso (%)	0.0 ± 0.1	0.1 ± 0.0	0.1 ± 0.1	0.0 ± 0.0	0.0 ± 0.1	0.0 ± 0.0
Eosi (%)	1.8 ± 0.5	1.8 ± 0.6	1.9 ± 0.5	1.6 ± 0.4	1.7 ± 1.3	1.8 ± 0.7
Neut (%)	26.5 ± 10.3	29.8 ± 12.8	28.4 ± 4.8	26.0 ± 9.5	25.3 ± 10.9	22.7 ± 4.3
Lymp (%)	66.1 ± 10.5	63.6 ± 12.9	63.8 ± 5.5	68.4 ± 10.3	68.6 ± 11.2	71.2 ± 4.0
Mono (%)	5.6 ± 0.8	4.8 ± 1.4	5.8 ± 1.3	3.9 ± 1.3	4.3 ± 1.5	4.4 ± 0.9
Baso (10^2/μL)	0.0 ± 0.1	0.1 ± 0.0	0.1 ± 0.1	0.0 ± 0.0	0.0 ± 0.1	0.0 ± 0.0
Eosi (10^2/μL)	1.6 ± 0.5	2.1 ± 0.6	1.8 ± 1.0	1.0 ± 0.3	1.0 ± 0.6	1.0 ± 0.5
Neut (10^2/μL)	25.2 ± 15.7	37.8 ± 27.8	27.1 ± 15.9	19.7 ± 17.4	16.3 ± 10.5	12.9 ± 4.6
Lymp (10^2/μL)	58.3 ± 11.7	72.7 ± 18.4	57.1 ± 18.0	43.3 ± 16.3	41.1 ± 20.0	40.4 ± 11.6
Mono (10^2/μL)	5.0 ± 1.3	5.6 ± 2.1	5.3 ± 2.4	2.7 ± 1.7	2.6 ± 1.1	2.4 ± 0.5

Values are the mean ± S.D.

Dunnett's/Steel's test was performed to compare the Control (Saline) group with each AAV9/3-*modHEXB* group [* p <0.05].

Table S10. Blood chemistry

	Male			Female		
	Saline	AAV9/3-*modHEXB*, Low-dose	AAV9/3-*modHEXB*, High-dose	Saline	AAV9/3-*modHEXB*, Low-dose	AAV9/3-*modHEXB*, High-dose
	n = 10	n = 10	n = 9	n = 9	n = 10	n = 10
AST (U/L)	70.8 ± 16.2	71.3 ± 7.0	83.2 ± 9.7	171.4 ± 288.7	102.7 ± 26.3	138.6 ± 86.7
ALT (U/L)	27.9 ± 5.7	25.1 ± 4.5	26.8 ± 4.5	73.8 ± 137.4	30.6 ± 7.5	56.4 ± 47.8
ALP (U/L)	71 ± 16	67 ± 14	63 ± 16	28 ± 9	30 ± 13	25 ± 6
LDH (U/L)	76 ± 22	63 ± 14	70 ± 9	90 ± 73	88 ± 32	102 ± 61
γ-GTP (U/L)	0.5 ± 0.2	0.3 ± 0.1	0.4 ± 0.2	0.5 ± 0.2	0.6 ± 0.3	0.6 ± 0.3
Glu. (mg/dL)	179 ± 30	158 ± 27	153 ± 18	135 ± 16	138 ± 21	146 ± 14
T.Cho. (mg/dL)	81 ± 15	75 ± 21	76 ± 17	89 ± 22	79 ± 19	85 ± 9
TG (mg/dL)	74 ± 26	72 ± 44	73 ± 52	33 ± 25	32 ± 20	34 ± 20
PL (mg/dL)	114 ± 17	111 ± 27	112 ± 24	168 ± 43	148 ± 35	161 ± 14
TP (g/dL)	6.3 ± 0.4	6.3 ± 0.4	6.3 ± 0.3	6.8 ± 0.4	6.7 ± 0.5	6.8 ± 0.3
Alb. (g/dL)	2.2 ± 0.1	2.2 ± 0.2	2.1 ± 0.2	2.8 ± 0.3	2.5 ± 0.5	2.8 ± 0.2
A/G	0.55 ± 0.07	0.53 ± 0.06	0.51 ± 0.06	0.73 ± 0.08	0.61 ± 0.13 *	0.72 ± 0.07
BUN (mg/dL)	12 ± 2	13 ± 2	15 ± 3	14 ± 1	15 ± 3	14 ± 3
Crea. (mg/dL)	0.28 ± 0.04	0.29 ± 0.04	0.29 ± 0.05	0.32 ± 0.04	0.31 ± 0.04	0.32 ± 0.06
T.Bil. (mg/dL)	0.07 ± 0.01	0.06 ± 0.01	0.06 ± 0.02	0.09 ± 0.05	0.07 ± 0.02	0.09 ± 0.02
Na (mEq/L)	143 ± 1	144 ± 1	144 ± 1	142 ± 1	142 ± 1	142 ± 1
K (mEq/L)	4.6 ± 0.3	4.5 ± 0.3	4.7 ± 0.3	3.9 ± 0.3	3.9 ± 0.3	3.8 ± 0.3
Cl (mEq/L)	108 ± 1	108 ± 2	109 ± 2	110 ± 1	109 ± 3	109 ± 1
P (mg/dL)	5.6 ± 0.5	5.5 ± 0.7	5.7 ± 0.6	4.4 ± 0.7	4.9 ± 0.6	4.6 ± 0.7
Ca (mg/dL)	10.1 ± 0.4	9.8 ± 0.4	9.8 ± 0.6	9.9 ± 0.4	10.0 ± 0.4	10.1 ± 0.2

Values are the mean ± S.D.

Dunnett's/Steel's test was performed to compare the Control (Saline) group with each AAV9/3-*modHEXB* group [* p <0.05].

Table S11. Urinalysis

		Male			Female		
		Saline n = 5	AAV9/3-*modHEXB*, Low-dose n = 5	AAV9/3-*modHEXB*, High-dose n = 5	Saline n = 5	AAV9/3-*modHEXB*, Low-dose n = 5	AAV9/3-*modHEXB*, High-dose n = 5
Color	Yellow	0	2	0	2	0	0
	Pale yellow	5	3	3	3	5	5
	Achromatic urine	0	0	2	0	0	0
pH	6.5	0	0	0	0	1	0
	7.5	0	0	0	0	0	1
	8.0	2	1	1	1	2	1
	8.5	3	4	4	4	2	3
Protein	Negative	0	2	1	3	4	4
	Trace	4	1	2	1	1	1
	30 mg/dL	1	2	1	1	0	0
	100 mg/dL	0	0	1	0	0	0
Glucose	Negative	5	5	5	5	5	5
Ketone	Negative	2	2	2	5	4	5
	5 mg/dL	2	2	2	0	1	0
	15 mg/dL	1	1	1	0	0	0
Urobilinogen	0.1–1 Ehrlich/dL	5	5	5	5	5	5
Bilirubin	Negative	5	5	5	5	5	5
Occult blood	Negative	5	5	3	4	4	4
	Trace	0	0	2	0	1	1
	Medium	0	0	0	1	0	0
Urinary sediments							
RBC	0 cell/field	5	5	5	4	5	5
	<5 cells/field	0	0	0	1	0	0
WBC	0 cell/field	2	3	3	2	5	3
	<6 cells/field	3	2	2	3	0	2
Epithelial cell	<6 (>0) cells/field	5	5	5	5	5	5
Cast	Negative	5	5	5	5	5	5
Crystal	Negative	3	3	3	3	3	5
	AMP	2	2	2	2	2	0
Others	Negative	5	5	5	5	5	5
Volume (mL)		15.8 ± 5.9	13.4 ± 4.7	28.4 ± 17.5	14.1 ± 4.9	20.2 ± 5.9	17.1 ± 5.2
Specific gravity		1.047 ± 0.010	1.051 ± 0.013	1.042 ± 0.022	1.040 ± 0.007	1.033 ± 0.008	1.039 ± 0.012
Electrolyte concentration							
Na (mEq/L)		68 ± 26	81 ± 34	80 ± 52	60 ± 23	63 ± 11	75 ± 28
K (mEq/L)		202.3 ± 44.3	218.9 ± 60.5	198.2 ± 111.0	179.9 ± 44.7	151.6 ± 34.7	187.2 ± 60.5
Cl (mEq/L)		109± 35	121± 39	120± 80	108 ± 33	92 ± 16	111 ± 38
Total excretion							
Na (mg/day)		25 ± 13	25 ± 12	36 ± 11	19 ± 7	29 ± 6 *	27 ± 3
K (mg/day)		121 ± 43	108 ± 29	160 ± 36	93 ± 18	114 ± 11	116 ± 10 *
Cl (mg/day)		61 ± 28	55 ± 21	82 ± 25	51 ± 14	64 ± 11	62 ± 3

Numerals represent the number of animals with the findings. Values are the mean ± S.D.

AMP: Ammonium magnesium phosphate

Dunnett's/Steel's test was performed to compare the Control (Saline) group with each AAV9/3-*modHEXB* group [* p <0.05].

Table S12. Gross pathological findings

	Male			Female		
	Saline<br>n = 10	AAV9/3-*modHEXB*, Low-dose<br>n = 10	AAV9/3-*modHEXB*, High-dose<br>n = 9	Saline<br>n = 9	AAV9/3-*modHEXB*, Low-dose<br>n = 10	AAV9/3-*modHEXB*, High-dose<br>n = 10
Brain (Ventricular dilatation)	0	1	0	0	0	0
Lung (Mottled grayish-white color, scattered, all lobules)	1	0	0	0	0	0
Hind limb (Lost, left, all fingers)	0	0	1	0	0	0
Spleen (Large size)	0	0	1	0	0	0

Numerals represent the number of animals with the findings.

Table S14. Anti-AAV9/3 antibody analysis

Group	Sex	Absorbance	Judgement
Saline (n = 5)	Male	0.032	Negative
		0.030	Negative
		0.028	Negative
		0.040	Negative
		0.029	Negative
	Female	0.049	Negative
		0.025	Negative
		0.027	Negative
		0.043	Negative
		0.027	Negative
AAV9/3-*modHEXB*, Low-dose (n = 5)	Male	3.754	Positive
		3.741	Positive
		3.803	Positive
		3.864	Positive
		3.728	Positive
	Female	3.152	Positive
		1.450	Positive
		3.775	Positive
		3.777	Positive
		3.790	Positive
AAV9/3-*modHEXB*, High-dose (n = 5)	Male	2.793	Positive
		3.808	Positive
		3.657	Positive
		3.072	Positive
		3.763	Positive
	Female	3.781	Positive
		3.671	Positive
		3.749	Positive
		3.753	Positive
		3.806	Positive

Absorbance of Negative Control sample: 0.025

Absorbance of Positive Control sample: 0.499

Cut point: 0.058

Supplemental references

[S1] Koo BS, Lee DH, Kang P, Jeong KJ, Lee S, Kim K, Lee Y, Huh JW, Kim YH, Park SJ, Jin YB, Kim SU, Kim JS, Son Y, Lee SR. Reference values of hematological and biochemical parameters in young-adult cynomolgus monkey (*Macaca fascicularis*) and rhesus monkey (*Macaca mulatta*) anesthetized with ketamine hydrochloride. *Lab. Anim. Res.* **35**, 7 (2019). https://doi.org/10.1186/s42826-019-0006-0

[S2] Bakker J, Maaskant A, Wegman M, Zijlmans DGM, Hage P, Langermans JAM, Remarque EJ. Reference intervals and percentiles for hematologic and serum biochemical values in captive bred rhesus (*Macaca mulatta*) and cynomolgus macaques (*Macaca fascicularis*). *Animals (Basel)*. **13**, 445 (2023). https://doi.org/10.3390/ani13030445
